# Integrated catalysis opens new arylation pathways via regiodivergent enzymatic C–H activation

**DOI:** 10.1038/ncomms11873

**Published:** 2016-06-10

**Authors:** Jonathan Latham, Jean-Marc Henry, Humera H. Sharif, Binuraj R. K. Menon, Sarah A. Shepherd, Michael F. Greaney, Jason Micklefield

**Affiliations:** 1 School of Chemistry, The University of Manchester, Oxford Road, Manchester M13 9PL, UK; 2 Manchester Institute of Biotechnology, The University of Manchester, 131 Princess Street, Manchester M1 7DN, UK

## Abstract

Despite major recent advances in C–H activation, discrimination between two similar, unactivated C–H positions is beyond the scope of current chemocatalytic methods. Here we demonstrate that integration of regioselective halogenase enzymes with Pd-catalysed cross-coupling chemistry, in one-pot reactions, successfully addresses this problem for the indole heterocycle. The resultant ‘chemobio-transformation' delivers a range of functionally diverse arylated products that are impossible to access using separate enzymatic or chemocatalytic C–H activation, under mild, aqueous conditions. This use of different biocatalysts to select different C–H positions contrasts with the prevailing substrate-control approach to the area, and presents opportunities for new pathways in C–H activation chemistry. The issues of enzyme and transition metal compatibility are overcome through membrane compartmentalization, with the optimized process requiring no intermediate work-up or purification steps.

## Abstract (web summary)

Biocatalysis and metal catalysis often provide complimentary reactivities and selectivities. Here, the authors exploit the regioselectivity of an enzymatic C–H activation followed by palladium catalysed carbon-carbon bond formation in one pot, with membrane compartmentalization used to isolate the two chemistries.

## Graphical abstract

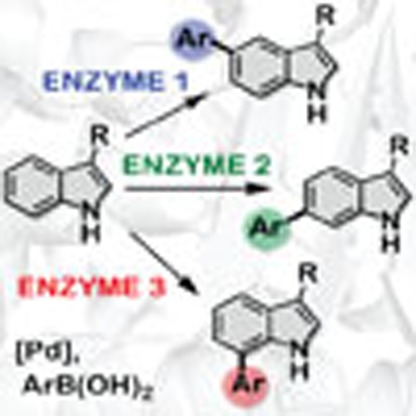

Selective activation of C–H positions has transformed synthesis in recent years. Using transition metal catalysis, a plethora of direct, fundamental bond formations are now achievable under mild, catalytic conditions, changing the way chemists make molecules^1^. The field is currently defined by substrate control, with a relatively small number of C–H motifs undergoing predictable and reliable metalation by a transition metal catalyst; for example, electron-rich heteroarenes, electron-poor fluoroarenes, activation ortho (and more recently, meta), to a directing group via cyclometalation all being dominant examples (Fig. 1a)^2^,^3^,^4^,^5^,^6^,^7^,^8^,^9^. Effective catalyst control, where choice of catalyst can discriminate between neighbouring C–H bonds in the absence of strong substrate-directing effects, is very rare and remains an outstanding challenge in the field^10^,^11^,^12^,^13^. Progress in this area will profoundly enhance the scope of C–H activation, as the majority of C–H bonds do not fit the current substrate-control criteria for chemocatalytic activation chemistry.

We were interested in addressing this problem using a combination of bio- and chemo-catalysis. The complex coordination sphere of an enzyme active site enables exquisite selectivities that cannot be achieved by simple metal coordination complexes. Correspondingly, the manifold power of noble metal catalysis in C–C bond formation presents synthesis pathways that are not possible through biocatalysis. Their integration offers a complementarity that could create new C–H activation strategies, whereby C–H positions beyond the scope of chemocatalysis alone can be targeted for C–C, C–N, C–Hal and other fundamental bond-forming steps. In addition, the merging of bio- and chemo-catalysis into single process, one-pot operations creates a step-change in efficiency through enhanced space-time yields, better energy consumption and elimination of auxiliary chemicals^14^,^15^.

Despite the potential advantages of combining biocatalysts with chemocatalysts, their successful integration has been largely restricted to organic solvents^15^,^16^,^17^, which are incompatible with many enzymes and cofactors—therefore limiting the number of biocatalysts that can be utilized. In contrast, integration of chemocatalysts with enzymes in their preferred aqueous media is relatively rare^14^. Indeed, the inherent differences in operating conditions of enzymes and chemocatalysts present serious challenges, often requiring compartmentalization or immobilization of at least one of the catalysts^18^,^19^,^20^,^21^,^22^,^23^.

Our plans for a catalyst-controlled, integrated C–H activation system involve the merging of flavin-dependent halogenase (Fl-Hal) enzymes with palladium-catalysed cross-coupling. The pivotal role played by aryl halide functionality in metal-catalysed transformations suggested to us that a regioselective, biohalogenation would have great versatility, with the incipient Ar-X group being adaptable to a multitude of Pd-catalysed chemistries. We chose to integrate Fl-Hals and Suzuki–Miyaura coupling (SMC) with boronic acids in the first instance, given the critical role of SMC in producing biaryl products for pharmaceutical, agrochemical and materials science applications.

The most widely studied Fl-Hals are tryptophan halogenases, which are responsible for chlorinating different positions of the indole ring of tryptophan *en route* to various bacterial natural products^24^,^25^,^26^,^27^,^28^,^29^. Several fungal Fl-Hals have also been reported that chlorinate phenolic natural products^30^,^31^,^32^. Together this suite of enzymes have the potential to regioselectively halogenate a range of aromatic scaffolds. With appropriate cofactor-recycling systems (Fig. 1b) Fl-Hals can afford halogenation reactions using benign inorganic halide (typically MgCl_2_ or NaBr), oxygen (from air), and glucose or isopropanol as the only stoichiometric reagents^33^,^34^. Studies on broadening the substrate scope of the tryptophan halogenases RebH, PrnA and PyrH^33^,^34^,^35^,^36^, as well as improving their productivity and scaleability^37^, have meant that Fl-Hals are becoming increasingly viable as biocatalysts for aromatic halogenation. In addition, Fl-Hals have been used to halogenate natural products and synthetic substrates, which have been isolated or extracted and then derivatized in separate cross-coupling reactions under standard synthetic operating procedures^38^,^39^,^40^. Biohalogenation is also attractive, given that traditional aromatic halogenation chemistry utilizes deleterious reagents, lacks regiocontrol and can afford mixtures, including halogenated by-products that can be toxic and persistent in the environment.

We anticipated that the unique regiocontrol inherent to Fl-Hal enzymatic halogenation could set-up an overall catalyst-controlled C–H arylation system, if a working SMC could be successfully integrated under aqueous conditions. The indole heterocycle has been central to the development of contemporary C–H activation chemistry, driving the discovery of new chemocatalytic methods for functionalizing the C2 and C3 positions (the ‘innate' positions for functionalization with an electrophile)^41^,^42^,^43^,^44^,^45^,^46^. Direct C–C bond formation at the arene C_6_–H and C_7_–H positions, however, is not currently possible using chemocatalysis—something we hoped to achieve with the integrated approach.

## Results

### Biocatalytic halogenation

**Figure 1:**
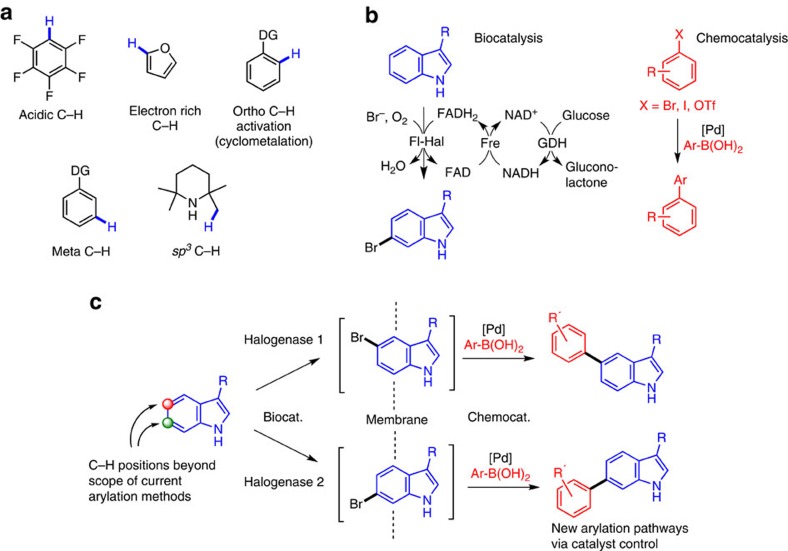
Previous work on C–H activation and proposed scheme of catalyst control using biohalogenation and Pd-catalysed cross-couplings. (**a**) Previous work on C–H activation utilizing substrate control of regioselectivity. (**b**) Catalytic cycle of halogenase biocatalysis using a Fl-Hal, along with flavin reductase (Fre) and glucose dehydrogenase (GDH) enzymes for cofactor recycling. (**c**) Proposed scheme of regioselective C–H activation using catalyst control by integration of a Fl-Hal and palladium-catalysed SMC. Biocat., biocatalysis; chemocat., chemocatalysis.

**Figure 2:**
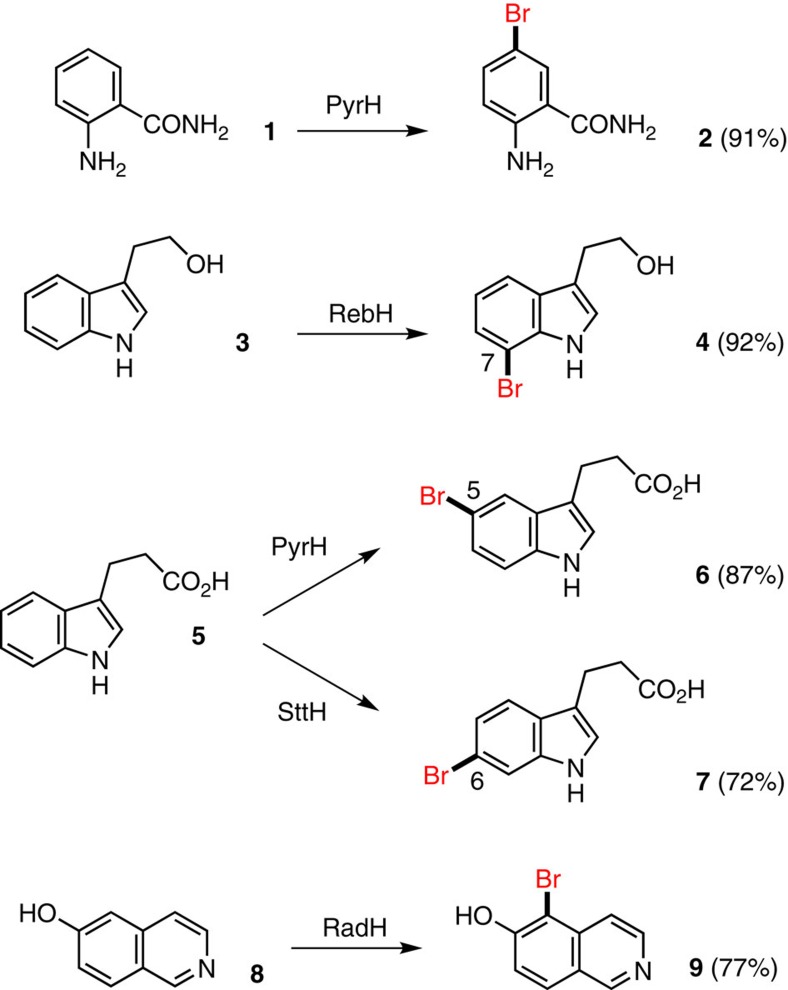
The regioselective and regio-divergent bromination of a range of aromatic scaffolds. Regioselective halogenation of anthranilamide (**1**), tryptophol (**3**), 3-indole propionate (**5**) and 6-hydroxy isoquinoline (**8**) using a range of Fl-Hals to access different regiochemistries.

To effect one-pot Fl-Hal-SMC reactions (Fig. 1c), we required enzymes and substrates that afford high yields of structurally diverse brominated products. First, we exploited our previous findings^33^ that the tryptophan 5-halogenase PyrH can accept anthranilamide (**1**) to develop conditions for effective enzymatic synthesis of 5-bromoanthranilamide (**2**) (Fig. 2). Given the exquisite natural regio-complementarity of the flavin-dependent tryptophan halogenases we then set out to identify potential indole substrates for biocatalyst-controlled bromination and subsequent arylation. We found that tryptophol (**3**) could be efficiently brominated by RebH (a tryptophan 7-halogenase) to afford 7-bromotryptophol (**4**), after optimizing the conditions (Supplementary Fig. 1) that had been previously reported for chlorination of this substrate^34^. Initial attempts to brominate the 5-position of tryptophol (**3**) using PyrH in place of RebH gave very low conversion. The larger substrate 3-indolepropionic acid (**5**) however, was transformed by PyrH to give 5-bromo-3-indolepropionate (**6**) as a single regioisomer in good yield. In addition, the 6-bromo-3-indolepropionate (**7**) could be obtained in good regioisomeric excess and yield by using the tryptophan 6-halogenase SttH^27^. Collectively, these three halogenase enzymes established a clean, regiodivergent synthesis of C_5_, C_6_ and C_7_ bromoindoles.

Finally, to extend the substrate diversity further we overproduced the radicicol halogenase RadH enzyme from the fungus *Chaetomium chiversii*^30^. RadH is similar in sequence to Rdc2—a related fungal halogenase that had been shown previously to chlorinate 6-hydroxy-isoquinoline (**8**)^31^. Accordingly conditions were developed whereby RadH could be used to prepare 5-bromo-6-hydroxyisoquinoline (**9**) in good yields.

### Cross-coupling optimization

**Figure 3:**
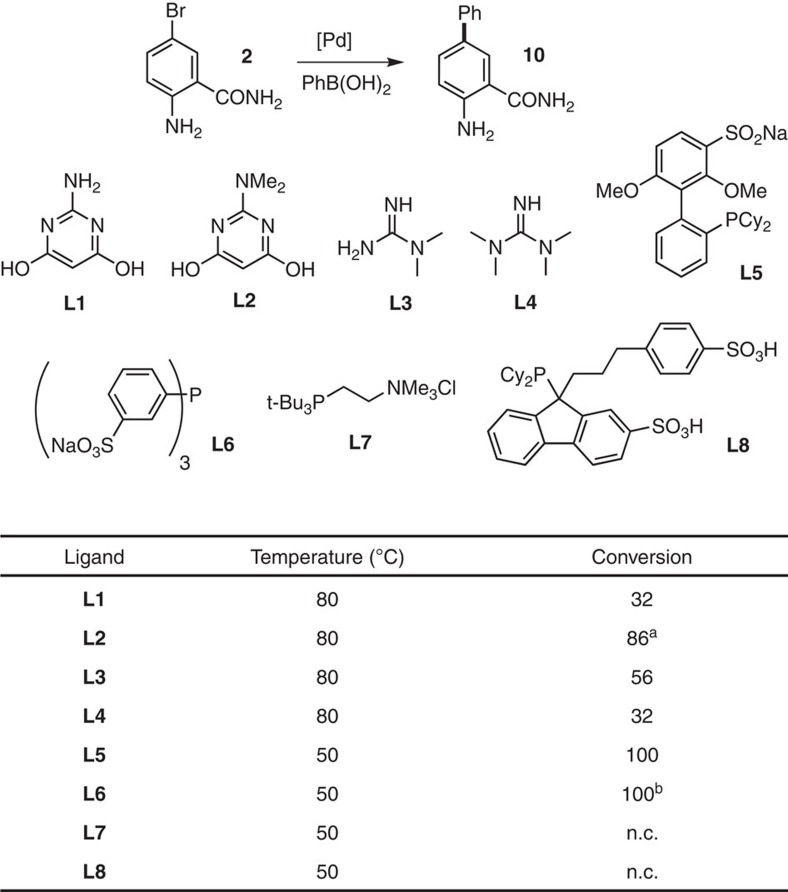
Ligand screening for the SMC of **2** with phenyl boronic acid. Pd(OAc)_2_ was used as the palladium source in each case in a 2:1 ligand:Pd ratio. Conversions determined by analytical high-performance liquid chromatography (HPLC) or liquid chromatography mass spectrometry (LC-MS). n.c., no conversion. ^a^81% isolated yield; ^b^80% isolated yield. Further optimization results are available in the Supplementary Material (Supplementary Fig. 2).

We next sought conditions whereby the SMC could be integrated with the biocatalysts, using the coupling of 5-bromoanthranilamide (**2**) with phenyl boronic acid as a test reaction (Fig. 3). On the basis of previous studies of SMCs in aqueous media with an open atmosphere^47^,^48^, a number of pyrimidine and guanidine ligands were selected for screening (**L1**–**L4**). Of these, **L2** afforded highest conversion to 5-phenylanthranilamide (**10**) at 2.0 mM substrate (**2**) concentration, 2.5 mol% Pd loading, in potassium phosphate buffer in the absence of any cofactors or enzymes. In addition, a number of water-soluble phosphine ligands were also screened (**L5**–**L8**). Of these, tppts (**L6**) and sulfonated SPhos (**L5**) gave full conversion to the desired product (**10**). These two ligands were therefore carried forward in a base and palladium source screen (Supplementary Fig. 2). The reaction was found to be tolerant of many bases, including DIPEA, whilst the lack of any base afforded no product. In addition, conducting the reaction using **L5** and **L6** under air as opposed to inert atmosphere afforded no conversion, whilst the same reaction using **L2** under open air afforded good conversion.

The tolerance of the selected Pd catalysts towards the biocatalytic halogenation conditions was then probed. As NAD^+^/NADH and FAD/FADH_2_ are rich in heteroatoms and redox active they could possibly interfere with Pd-catalysis either through redox chemistry or coordination to metal species. In addition, it has been previously reported that soft donor atoms (for example, sulfur) on the surface of proteins can coordinate to transition metals potentially inhibiting their catalytic activity. Pd^II^ is also known to react with glucose under certain conditions (utilized to recycle NADH). The test reaction (Fig. 3) was therefore run in the presence of each of these components to determine their effect on SMC conversion. This revealed a somewhat deleterious effect from the presence of the cofactors and a major reduction in cross-coupling yields in the presence of proteins (Supplementary Fig. 3). Increasing the loading of palladium catalyst was generally advantageous in terms of conversion in the presence of NADH, FAD and glucose, but did not enhance cross-coupling when either the reductase or dehydrogenase proteins were present (Supplementary Fig. 4).

### Halogenase SMC integration

**Figure 4:**
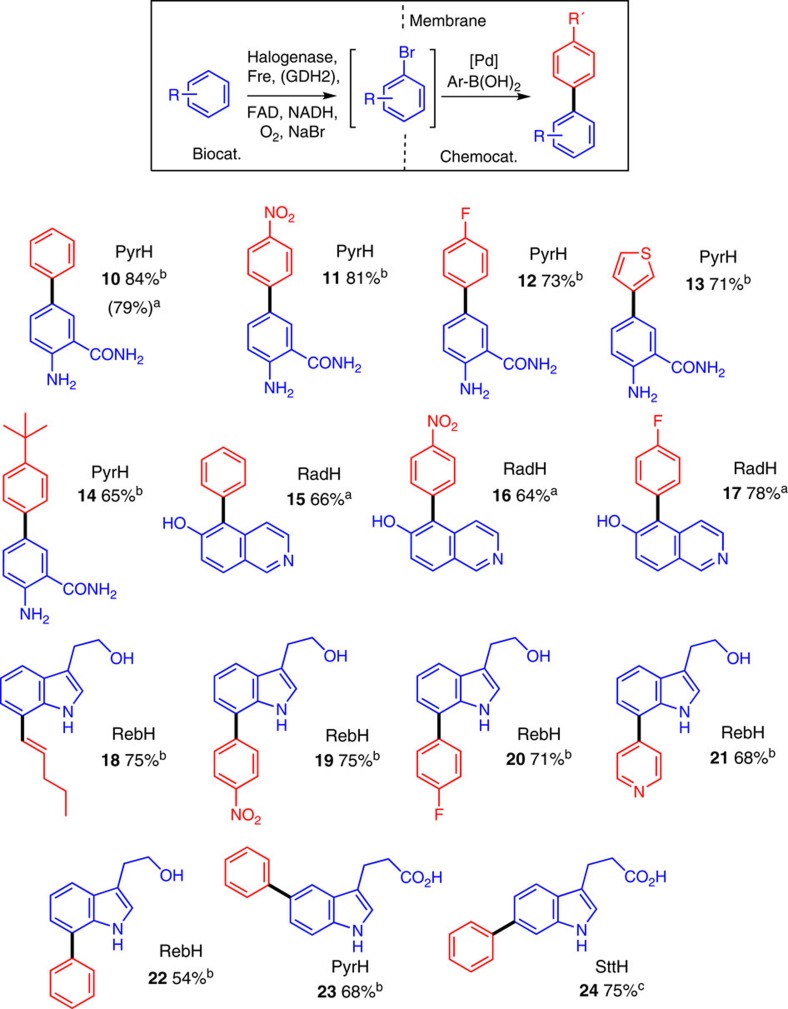
Isolated yield of the regioselective and regiodivergent arylation. ^a^Obtained using purified enzymes and a MWCO membrane (method A). ^b^Obtained using a CLEA of the appropriate Fl-Hal and 10 mol% Pd-catalyst loading (method B). ^c^Obtained from three successive cycles of halogenation using the same Fl-Hal. Biocat., biocatalysis; chemocat., chemocatalysis.

Given that the presence of proteins caused the greatest reduction in cross-coupling yields, we reasoned that the enzymes would need to be compartmentalized or removed before cross-coupling, at least in the first instance. Pleasingly, we found that passing the PyrH biotransformation of anthranilamide (**1**) through a 10-kDa molecular-weight cut-off (MWCO) cellulose membrane to remove protein before cross-coupling was effective in this regard, affording very good conversion to arylated product (**10**). Following further optimization around this process (Supplementary Fig. 5), we could achieve an overall isolated yield of 79% of arylated product (**10**) using tppts ligand **L6**, Pd(OAc)_2_ (50 mol%), and an excess of phenyl boronic acid (Fig. 4). Moreover, we showed that the enzymes that had been removed using the membrane filter could be recycled affording intermediate bromide (**2**) in 62% and 42% in the second and third cycles, respectively. Subsequent arylation of the combined biotransformation filtrates afforded arylated product (**10**) in 54% overall yield; a ca. twofold increase in the amount of material that would be obtained from the same amount of enzyme in a single arylation reaction. After determining the scope of this method with respect to boronic acids (Supplementary Fig. 6) and finding the SMC compatible with the conditions required for RadH-catalysed bromination of **8** (Supplementary Fig. 7), we then extended this membrane filtration method (method A) to the selective 5-arylation of 6-hydroxyisoquinoline (**8**), using RadH to produce **15**–**17** in good yields.

With a workable integrated arylation process in hand, we looked at ways to improve our method, principally with a view to increasing scale and reducing the high Pd content in the SMC reaction. The requirement for high catalyst loadings appeared to arise from a combination of dilute reaction volumes and inhibitory effect of cofactors present in the reaction medium. Indeed, other attempts to conduct palladium-catalysed chemistry in biological media and in the presence of proteins have required very high loading or suffered from poor yields^47^,^48^. Enhancing enzyme efficiency would mitigate these two issues, by affording higher concentrations of aryl bromide for cross-coupling. The group of Sewald have recently reported improvements in RebH productivity through the use of crosslinked enzyme aggregates (CLEAs)^37^. The individual subunits of RebH are crosslinked with each other, as well as the partner reductase and dehydrogenase proteins, via surface lysines using glutaraldehyde forming a RebH-reductase-dehydrogenase CLEA that is more efficient and tractable to handle^37^. Accordingly, we successfully prepared these materials as active heterogeneous biocatalysts containing either PyrH, SttH or RebH. Although active CLEAs of the phenolic halogenase RadH were also obtained, they did not prove to be significantly more efficient than purified enzyme.

Initial screening of the PyrH-CLEA with anthranilamide (**1**) demonstrated improved efficiency, affording up to 3.0 mM aryl bromide. This enabled one-pot halogenase-SMC reactions to proceed to reasonable conversion (ca. 50%) without the need to remove or compartmentalize the CLEA from the Pd catalyst and other reagents, presumably as many heteroatoms of the protein had been effectively protected during the crosslinking process (Supplementary Fig. 8). We also found that the arylation of tryptophol (**3**) could be carried out in the same one-pot manner, with the RebH-CLEA and the Pd catalyst both present throughout (Supplementary Fig. 8). However, highest conversions were obtained when the CLEA was removed, by centrifugation or filtration, before cross-coupling of the supernatant from biocatalytic halogenation (Supplementary Figs 8 and 9). The scope of this method (method B) was found to be broad with respect to boronic acids (Supplementary Fig. 10), allowing the efficient arylation of anthranilamide (**1**) with both electron-rich, electon-poor and heterocyclic boronic acids using PyrH-CLEA with reduced palladium loading (10 mol%), affording the 5-arylated compounds in very good yield (**10**–**14**, Fig. 4). Tryptophol (**3**) was also efficiently coupled using electron-rich, electron-poor, heterocyclic and alkenyl boronic acids to afford 7-substituted products **18** to **22** in good yield with RebH-CLEA at lower Pd loading. 3-Indole propionate (**5**) was likewise effectively arylated to biaryl products **23** and **24**, with PyrH- and SttH-CLEAs, respectively. Along with the tryptophol examples (**18**–**22**), this demonstrates the efficient and highly selective manipulation of each of the 5-, 6- and 7- C–H positions of the indole nucleus, under catalyst control, which is unattainable using existing synthetic methodology. In addition, the heterogeneous nature of the CLEA biocatalyst allowed it to be recycled over repeated cycles of bromination, with little loss in efficiency (Supplementary Fig. 11). In the case of the 6-arylation of 3-indolepropionic acid (**5**), this allowed an overall yield of 75% of **24** over three cycles with the SttH-CLEA. The increased stability and ease of preparation of the heterogeneous biocatalyst allowed this methodology to be scaled up to a 1.0-mmol scale to afford reasonable yields of **10** (>100 mg, 52%).

### Polydimethylsiloxane compartmentalization

To further enhance the scope and efficiency of the halogenase-SMC process, we looked for alternative ways of separating the proteins from the palladium catalyst. While membrane filtration worked well, it did introduce an additional operation in the middle of the process and necessitated a deoxygenation step before SMC. To better streamline the process and develop a more efficient one-pot procedure, we examined polydimethylsiloxane (PDMS) thimbles as a compartmentalization strategy. This approach had been reported for a number of reactions that require the separation of incompatible reagents^19^,^49^, including the combination of a Pd-catalyzed oxidation with a biocatalytic reduction^19^.

**Figure 5:**
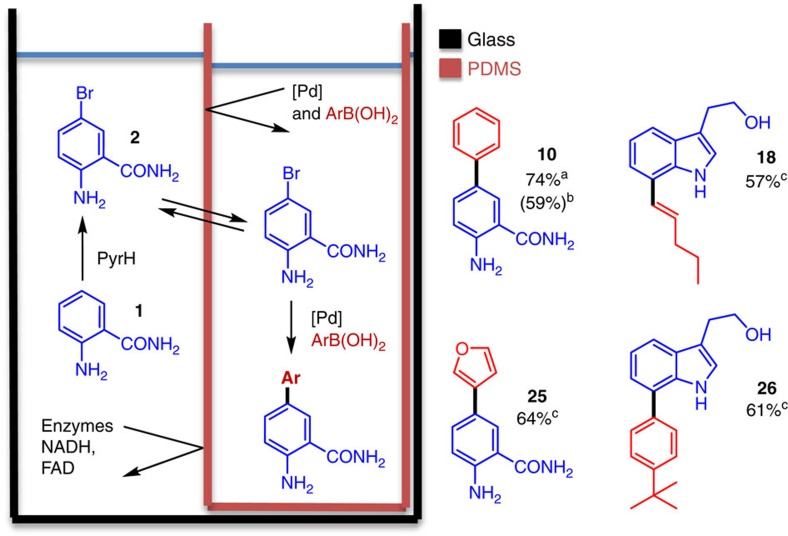
PDMS compartmentalization of the regioselective halogenation cross-coupling cascade. ^a^Obtained using purified enzyme and 10 mol% of **L2**.Pd(OAc)_2_. ^b^Obtained using halogenase CLEA and 2 mol% of **L2**.Pd(OAc)_2_. ^c^Obtained using halogenase CLEA and 10 mol% of **L2**.Pd(OAc)_2_.

Typically, one of the reactions is assembled inside a small thimble made of PDMS, which is then placed inside the larger bulk reaction vessel. As PDMS is a hydrophobic polymer, non-polar compounds (that is, substrates and intermediates in the cascade) should dissolve sufficiently well to pass through the PDMS walls, whilst charged reagents (Pd catalysts, enzymes and cofactors) would be unable to diffuse through PDMS and might therefore be compartmentalized. Initial tests showed that 5-bromoanthranilamide (**2**) would flux through PDMS well while glucose, NADH, FAD and the **L2**:Pd(OAc)_2_ complex were all unable to effectively penetrate PDMS (Supplementary Fig. 12). SMC conditions using **L2**:Pd(OAc)_2_ were therefore chosen to test the PDMS compartmentalization approach, as this catalyst functions well under open air and because alternative phosphine ligands have been shown to promote flux through PDMS. Accordingly conditions were optimized, which allowed the PyrH-halogenation of anthranilamide (**2**) using pure protein to be carried out with the cross-coupling reagents within the PDMS thimble (Fig. 5), resulting in a ‘one-pot' reaction to give 5-phenylanthranilamide (**10**) in 74% yield. The immediate advantages of this approach (method C) are that protein removal and deoxygenation of the reaction buffer are not required. Moreover a lower catalyst loading (10 mol%) is achievable under this regime, compared with the removal of pure protein using filtration (50 mol%), due to the compartmentalization of cofactors and enzymes from the Pd catalyst. By using a PyrH-CLEA to afford higher concentration of aryl bromide in conjunction with PDMS compartmentalization (method D), the Pd loading was further reduced to 2 mol% to afford **10** in good yield (59%). This combination also allowed the coupling of deactivated, electron-rich, boronic acids such as 3-furyl boronic acid to give **25** in good yield (64%), a reaction that was unsuccessful using a cellulose membrane (MWCO filter). Finally, this method was also extended to include coupling of the 7-position of tryptophol (**3**), using RebH-CLEA, to give 7-pent-1-enyl tryptophol (**18**) and 4′-tertbutyl-7-phenyl tryptophol (**26**) in good yields.

## Discussion

In summary, we have combined four regioselective flavin-dependent halogenase enzymes with a palladium-catalysed Suzuki–Miyaura cross-coupling to afford the regio-controlled arylation of a number of aromatic scaffolds (benzamides, isoquinolines and indoles). In each case, the regioselective C–H arylation transformations were previously inaccessible using stand-alone chemocatalysis or biocatalysis methods. Specifically, we report the selective C–H arylation of the 5-, 6- and 7- positions of the indole nucleus under catalyst control.

To overcome issues of catalyst incompatibility, we initially developed a filtration method using a 10-kDa MWCO filter (method A). This allowed efficient biocatalyst recycling for a number of cycles, affording approximately double the overall productivity that the same batch would in a single cycle. Biocatalyst efficiency was further improved by using CLEAs, which afforded higher aryl bromide concentration—enabling lower palladium loading to be employed (method B). We also developed a PDMS thimble system to compartmentalize the chemo- and bio-catalysts. This method afforded similar yields of regioselectively arylated product as the MWCO filter system, but without the need for protein removal or solution deoxygenation before the cross-coupling chemistry (method C). Finally, the implementation of CLEAs with PDMS compartmentalization led to a further reduction in the required Pd loading down to 2 mol% (method D). Combined with directed evolution and other research aimed at improving the productivity and scalability of halogenase enzymes^37^,^50^, the integrated method described here sets out a pathway to new C–H activation transformations that cannot be achieved through stand-alone biocatalysis or chemocatalysis. Research in this area is ongoing in our laboratories.

## Methods

### Method A: regioselective arylation 6-hydroxyisoquinoline

6-hydroxyisoquinoline (**8**) (0.5 mM), NaBr (10 mM), FAD (1 μM), Fre (4 μM), RadH (25 μM) and NADH (2.5 mM) were added to 10 mM potassium phosphate buffer (pH 7.2) containing 1% v/v EtOH (40 ml) and incubated with shaking overnight (30 °C) before filtration through a 10-kDa MWCO filter (Vivaspin 20). CsF (4.8 eq) and boronic acid (30 eq) were then added to the filtrate. Following freeze-thaw degassing and backfill with N_2_, tppts (0.5 mM) and Na_2_PdCl_4_ (0.25 mM) were added as deoxygenated stock solutions in water. The resultant solution was heated to 80 °C with stirring for 24 h. After cooling, pH was adjusted to 6 using HCl, and the reaction partitioned into CH_2_Cl_2_ before the removal of solvent *in vacuo* and purification.

### Method B: regioselective arylation using CLEAs

Substrate (3.0 mM), FAD (10 μM), NaBr (30 mM), NADH (100 μM) were dissolved in 15 mM sodium phosphate buffer with 5% v/v isopropanol (30 ml, pH 7.4). Halogenase CLEA from 1.5 l culture (prepared as described on page S46 of the Supplementary Methods) was then resuspended into the reaction buffer and the resultant suspension incubated at room temperature with orbital shaking overnight. After removal of CLEA by centrifugation (10,000 r.p.m., 4 °C, 30 min), K_3_PO_4_/K_2_CO_3_ (10 eq) and boronic acid (5 eq) were added to the supernatant before degassing of the solution with a stream of nitrogen. Tppts (20 mol%) and Na_2_PdCl_4_ (10 mol%) were then added and the solution was stirred at 80 °C overnight. On cooling, the reaction was partitioned into EtOAc/CH_2_Cl_2_ and concentrated before purification.

### Method C: regioselective arylation using PDMS thimbles

To a solution containing anthranilamide (2.0 mM), NaBr (100 mM), FAD (1 μM), glucose (20 mM), PyrH (20 μM), Fre (2 μM) and GDH2 (12 μM) in 10 mM potassium phosphate buffer (30 ml, pH 7.2) was added NADH (100 μM). A PDMS thimble containing **L2.**Pd(OAc)_2_ (10 mol%), CsF (10 eq) and PhB(OH)_2_ (5 eq) in water was then placed inside the Erlenmeyer flask containing the biotransformation. After incubation at room temperature overnight, the reaction was heated to 80 °C for 8 h. After cooling, the inner and outer chambers were combined and basified with 4 N NaOH. The thimble was then soaked in EtOAc to flux out any residual product, and the reaction mixture extracted into EtOAc before concentration and purification.

### Method D: regioselective arylation using CLEAs and PDMS

Halogenase CLEA from 3 l of culture (prepared as described on page S46 of the Supplementary Methods) was suspended into 30 ml of reaction buffer containing anthranilamide (**1**) or tryptophol (**3**) (5.0 mM), NADH (100 μM), FAD (10 μM) and NaBr (30 mM) in 15 mM sodium phosphate buffer with 5% v/v isopropanol (30 ml, pH 7.4). To this suspension was added a PDMS thimble containing **L2.**Pd(OAc)_2_ (10 mol%), CsF (10 eq) and ArB(OH)_2_ (5 eq). After incubation with shaking at room temperature overnight, the reaction was heated to 80 °C for a further 24 h. Reactions were then worked up as previously described. To facilitate the recycling of the biocatalysts, the CLEA can be removed using centrifugation (10,000 r.p.m., 30 min., 4 °C) after overnight incubation at room temperature before addition of the PDMS thimble containing the SMC components.

### Data availability

The authors declare that the data supporting the findings of this study are available within the article and its Supplementary Information Files.

## Additional information

**How to cite this article:** Latham, J. *et al*. Integrated catalysis opens new arylation pathways via regiodivergent enzymatic C–H activation. *Nat. Commun.* 7:11873 doi: 10.1038/ncomms11873 (2016).

## Supplementary Material

Supplementary Information Supplementary Figures 1-50, Supplementary Methods and Supplementary References
