# Supplementary material for: Integrated catalysis opens new arylation pathways via regiodivergent enzymatic C–H activation
Source: Nat Commun. 2016 Jun 10;7:11873. doi: 10.1038/ncomms11873 (PMC4906404; doi:10.1038/ncomms11873)
Supplement: Supplementary Information — Supplementary Figures 1-50, Supplementary Methods and Supplementary References [file ncomms11873-s1.pdf]

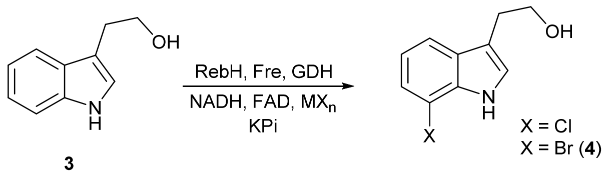

Halide	[FAD], µM	[MXn], mM	[RebH], µM	% X-Ar[a]
$MgCl_2$	100	10	25	51
NaBr	100	10	25	<5%
NaBr	100	100	25	12
NaBr	100	1000	25	NC
$MgCl_2$	100	10	50	98
NaBr	100	100	50	99[b]

Supplementary Figure 1: Optimisation of the bromination of tryptophol (**3**) by RebH. **Conditions:** Substrate (0.5 mM), NADH (100 µM), Fre (2.5 µM), GDH2 (5 µM), glucose (20 mM), KPi (10 mM) pH 7.2, isopropanol (4 %, v/v), 20 °C, overnight. [a] Conversion based on analytical HPLC. [b] isolated = 92%.

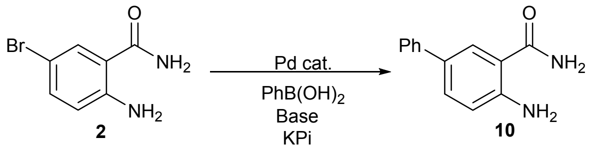

Pd Source	Base	Solvent	Atmosphere	Conversion[a]
$Pd(OAc)_2$	K_3PO_4	KPi	N_2	100
$Pd(OAc)_2$	K_3PO_4	KPi:ACN	N_2	82
$Pd(OAc)_2$	DIPEA	KPi	N_2	76
$Pd(OAc)_2$	DIPEA	KPi:ACN	N_2	64
$Pd(OAc)_2$	K_2CO_3	KPi:ACN	N_2	100[b]
$Pd(OAc)_2$	K_2CO_3	KPi	Air	NC
$Pd(OAc)_2$	K_2CO_3	KPi:ACN	Air	NC
$Pd(OAc)_2$	None	KPi	N_2	NC
$Pd(OAc)_2$	None	KPi:ACN	N_2	NC
$Pd(OAc)_2$	None	KPi	Air	NC
$Na_2[PdCl_4]$	K_3PO_4	KPi:ACN	N_2	100

Supplementary Figure 2: Base and palladium source screen for the Suzuki-Miyaura coupling of 5-bromo anthranilamide (**2**) with phenyl boronic acid using tppts (**L6**) as ligand. **Conditions:** 5-bromo anthranilamide (2.0 mM), Pd salt (2.5 mol %), tppts (6.25 mol %), boronic acid (1.2 eq), base (2.0 eq), 10 mM potassium phosphate buffer to total volume of 10 mL. 50 °C, overnight. [a] conversion based on LC-MS. [b] isolated = 80 %.

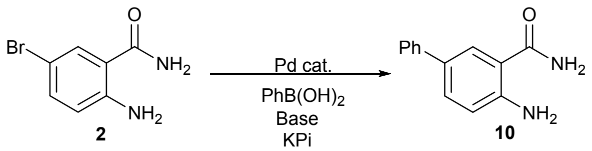

Catalyst	Additive (Concentration)	Conversion (%)[a]
**L2.**Pd(OAc)$_2$	None	29.5
	NADH (0.1 mM)	14.5
	FAD (1 μM)	18.8
	Fre (1 μM)	25.2
	GDH2 (6 μM)	0.8
	Glucose (40 mM)	10.4
**L6.**Na$_2$PdCl$_4$	None	20.6
	NADH (0.1 mM)	9.0
	FAD (1 μM)	13.0
	Fre (1 μM)	10.4
	GDH2 (6 μM)	2.2
	Glucose (40 mM)	17.2

Supplementary Figure 3: Effect of co-factors and enzymes on of the Suzuki-Miyaura coupling of 5-bromoanthranilamide (**2**) with phenyl boronic acid using **L2**:Pd(OAc)$_2$ (conditions above) and tppts$_2$.Na$_2$PdCl$_4$.Conditions: 5-bromoanthranilamide (2.0 mM), Na$_2$PdCl$_4$ (2.5 mol %), tppts (5.0 mol %), K$_3$PO$_4$ (2.4 mM), PhB(OH)$_2$ (2.0 eq), potassium phosphate buffer (10 mM, pH = 7.2), 80 ^{o}C, overnight. [a] Conversion based on analytical HPLC and calibration curve of 5-phenylanthranilamide.

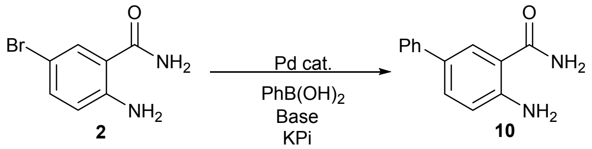

(A)

	Conversion% [a]			
**Additive**	**2.5**	**5**	**10**	**20 mol %**
None	9.1	61.7	93.1	78.2
NADH (0.1 mM)	2.9	13.5	16.6	14.3
FAD (1 μM)	12.3	18.8	54.4	90.2
Fre (1 μM)	4.7	10.8	4.0	0.5
GDH2 (6 μM)	0.4	1.8	8.0	1.8
Glucose (40 mM)	10.4	55.3	92.4	51.9

(B)

	Conversion% [a]			
**Additive**	**2.5**	**5**	**10**	**20 mol %**
None	20.6	40	62.3	9.2
NADH (0.1 mM)	9.0	25.5	29.8	2.6
FAD (1 μM)	13.0	33.7	38.3	21.1
Fre (1 μM)	10.4	38.9	39.0	14.7
GDH2 (6 μM)	2.2	3.5	3.0	1.6
Glucose (40 mM)	17.2	34.8	61.4	63.4

Supplementary Figure 4: Effect of increased Pd catalyst loading on tolerance of Suzuki-Miyaura reaction of 5-bromoanthranilamide (**2**) using (**A**) **L2**:Pd(OAc)$_2$ and (**B**) **L6**:Na$_2$PdCl$_4$. Conditions as above, expect (**A**) is left overnight also. [a] Conversion based on analytical HPLC and calibration curve of 5-phenylanthranilamide.

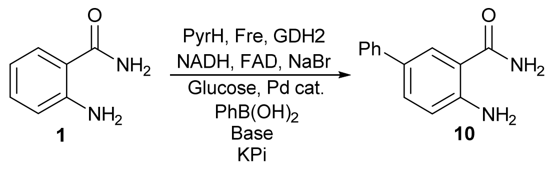

		Conversion(%) [a]	
**Loading (mol %)**	**Equiv $PhB(OH)_2$**	**L2**:$Pd(OAc)_2$	**L6**:Na_2PdCl_4
**40**	**20**	18.0	60.4
**40**	**30**	11.5	32.0
**50**	**30**	6.1	82.7

Supplementary Figure 5: Effect of increasing palladium catalyst loading and boronic acid equivalents on conversion of anthranilamide (**1**) to 5-phenyl anthranilamide (**10**) after using a celluose molecular-weight cut-off filter to remove biocatalysts from the biotransformation (Method A). [PyrH] = 20 μM, [Fre] = 2 μM, [GDH2] = 12 μM, [NADH] = 100 μM, [FAD] = 1 μM, [glucose] = 20 mM. [a] Conversion based on analytical HPLC and calibration curve of 5-phenylanthranilamide.

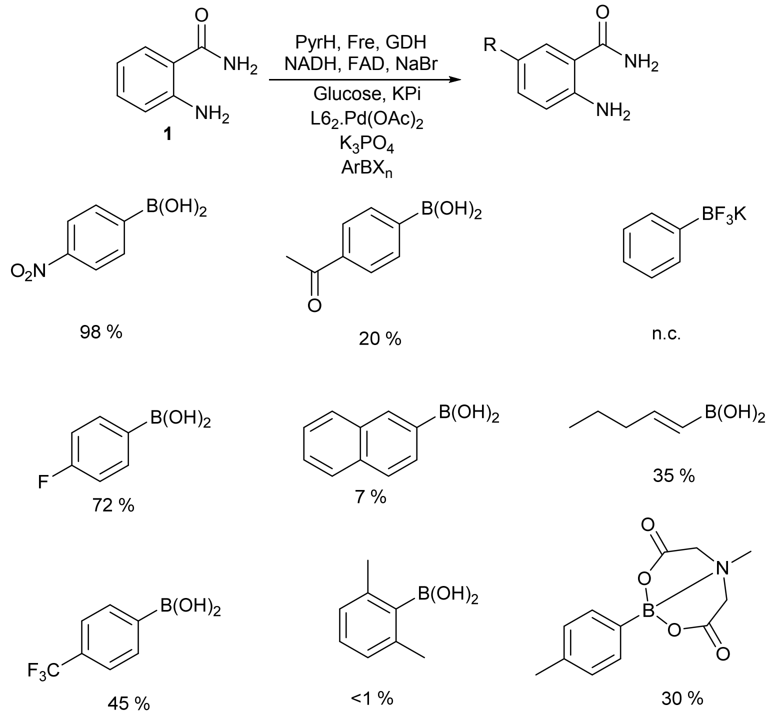

Supplementary Figure 6: Coupling partners tested in integrated reaction for arylation of anthranilamide using the integrated halogenase-Suzuki reaction with 10 kDa molecular weight cut-off filtration (method A). Conversion is based on 5-bromoanthranilamide consumption using analytical HPLC. Conditions as above using the tppts catalyst system.

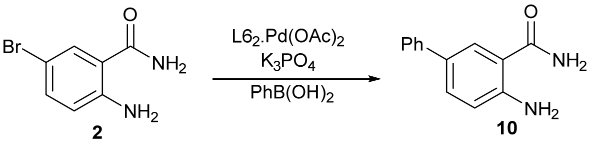

[NADH], mM	[FAD], µM	%Conversion[a]
0.1	1	92
5	1	96
0.1	100	93
5	100	87

Supplementary Figure 7: Effect of increased concentration of NADH and FAD on the Suzuki coupling of 5-bromo anthranilamide (**2**) with phenyl boronic acid. Conditions as prior using **L6**:Na_2PdCl_4. [a] Conversion based on analytical HPLC and calibration curve of 5-phenylanthranilamide.

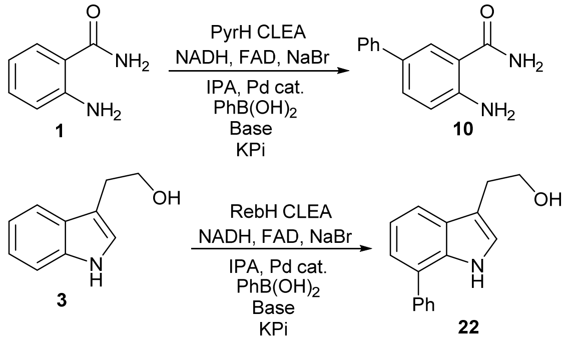

Loading (mol %)	Pd Cat.	CLEA Present	Conversion, 1→10 (%) [a]	Conversion, 3→22 (%) [b]
**10**	L6.Na_2PdCl_4	**Y**	12	27
**50**	L6.Na_2PdCl_4	**Y**	48	49
**10**	L6.Na_2PdCl_4	**N**	97	73
**50**	L6.Na_2PdCl_4	**N**	94	90
**10**	L2.Pd(OAc)$_2$	**Y**	NC	NC
**50**	L2.Pd(OAc)$_2$	**Y**	24	26
**10**	L2.Pd(OAc)$_2$	**N**	51	68
**50**	L2.Pd(OAc)$_2$	**N**	40	42

Supplementary Figure 8: Effect of reducing palladium catalyst loading and number of equivalents of boronic acid and removing heterogeneous biocatalyst (CLEA) from the reaction mixture on the integrated arylation (Method B) of anthranilamide (**1**) to 5-phenyl anthranilamide (**10**) and tryptophol (**3**) to 7-phenyl tryptophol (**16**). [Substrate] = 3 mM, [NADH] = 100 µM, [FAD] = 10 µM, [NaBr] = 30 mM, propan-2-ol (5 % v/v), sodium phosphate buffer (15 mM, pH = 7.2), halogenase CLEA from 1.5 L culture, [K_3PO_4] = 2.4 mM, [PhB(OH)$_2$] = 15 mM, 80 ^{o}C, overnight. [a] Conversion based on analytical HPLC and calibration curve of 5-phenylanthranilamide. [b] Conversion based consumption of 3 and intermediate bromide 4 by analytical HPLC and appropriate calibration curves. NC = no conversion.

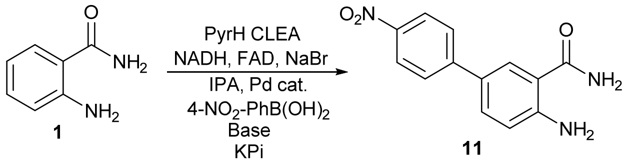

Loading (mol %)	Equiv 4-NO$_2$-PhB(OH)$_2$	Conversion(%) [a]
50	30	87
50	10	94
50	5	98
50	2	96
10	30	80
10	10	89
10	5	82
10	2	95

Supplementary Figure 9: Effect of reducing palladium loading and equivalents of boronic acid on the integrated arylation of anthranilamide (**1**) to 5-(4-nitrophenyl) anthranilamide (**11**) after removal of heterogeneous biocatalyst (Method B). Conditions as above. [a] Conversion based on analytical HPLC and calibration curve of 5-(4-nitrophenyl)anthranilamide.

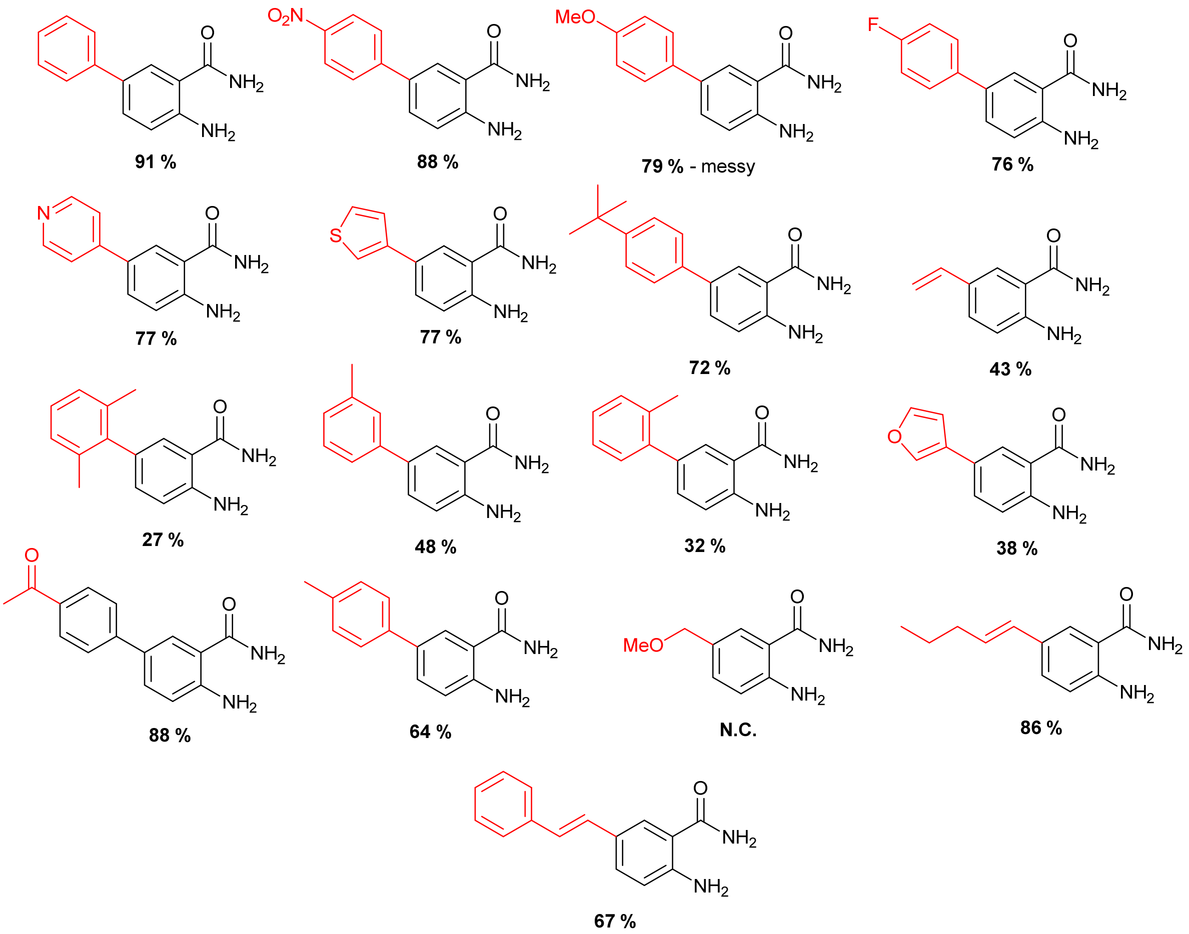

Supplementary Figure 10: Boronic acid coupling partners tested on an analytical scale for arylation of anthranilamide (**1**) using the integrated halogenase-Suzuki reaction with PyrH CLEAs and 10 mol% of Pd catalyst according to Method B in the experimental section of the main text. Conversions based on the consumption of anthranilamide (**1**) and the intermediate 5-bromoanthranilamide (**2**) by analytical HPLC and appropriate calibration curves. Products were confirmed by LC-MS and HRMS. Selected examples were repeated on a preparative scale for full product characterisation (see Figure 4 in the main text).

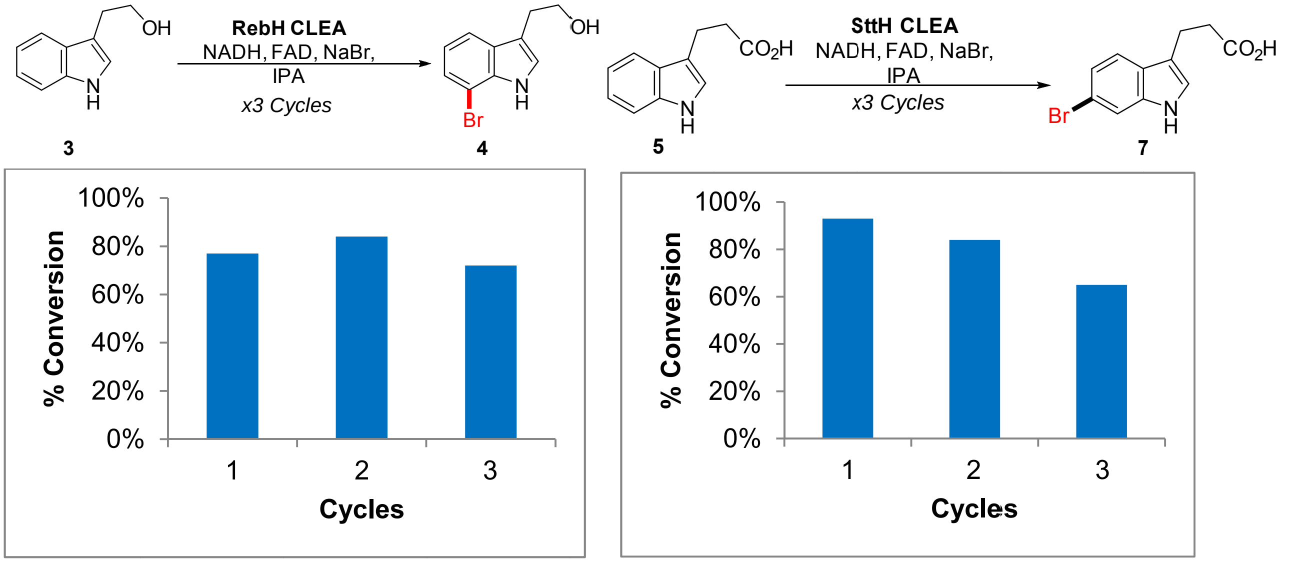

Supplementary Figure 11: Efficiency of RebH- and SttH-combi CLEAs over repeated cycles of bromination. Conversion based on analytical HPLC.

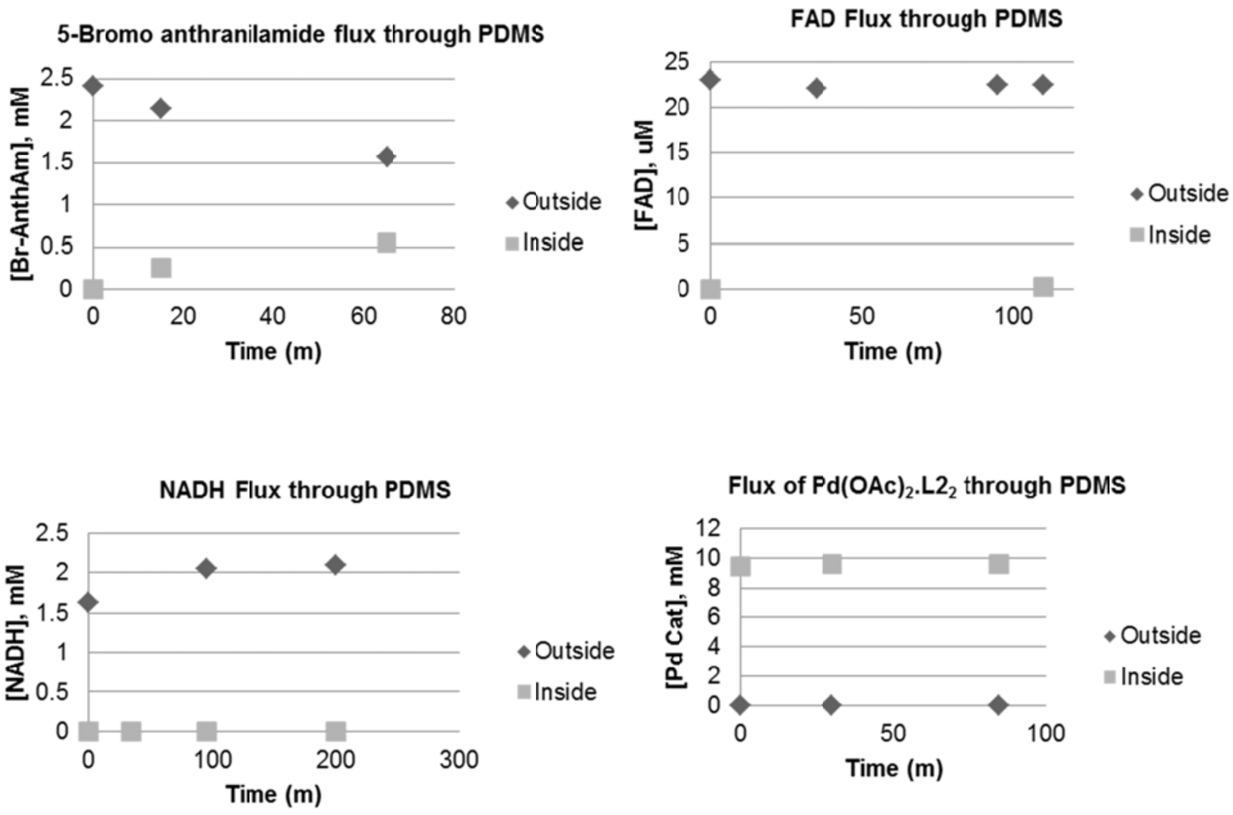

Supplementary Figure 12: Change in concentration of 5-bromoanthranilamide (**2**), FAD, NADH and the **L2**$_2$.Pd(OAc)$_2$ complex either side of a PDMS membrane over time.

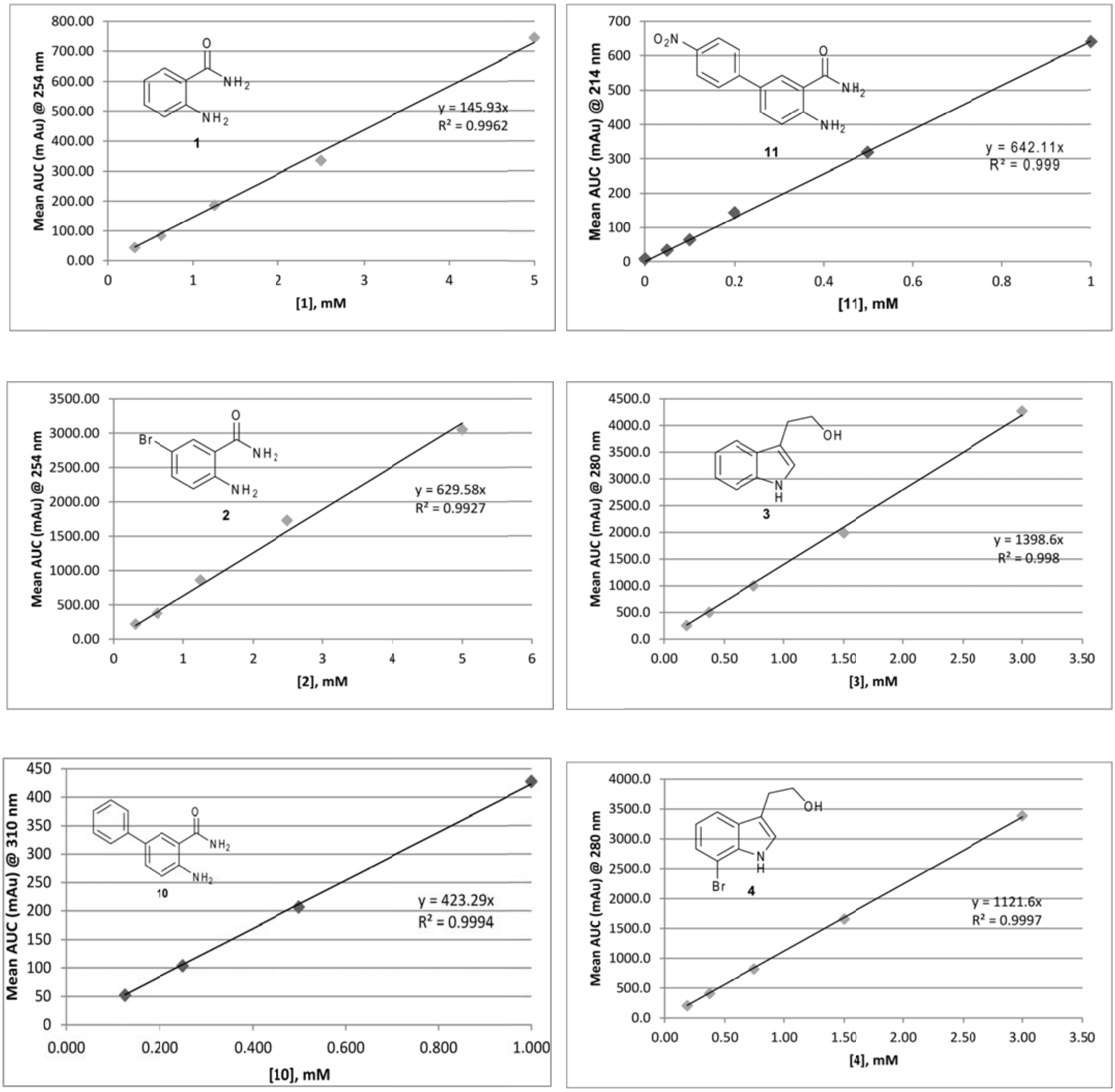

Supplementary Figure 13: Calibration curves of anthranilamide (**1**), 5-bromo anthranilamide (**2**), 5-phenyl anthranilamide (**10**), 5-(4'-nitro)-phenyl anthranilamide (**11**), trpytophol (**3**) and 7-bromo tryptophol (**4**) for analytical HPLC.

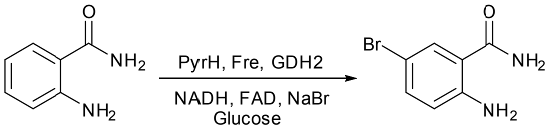

1 2

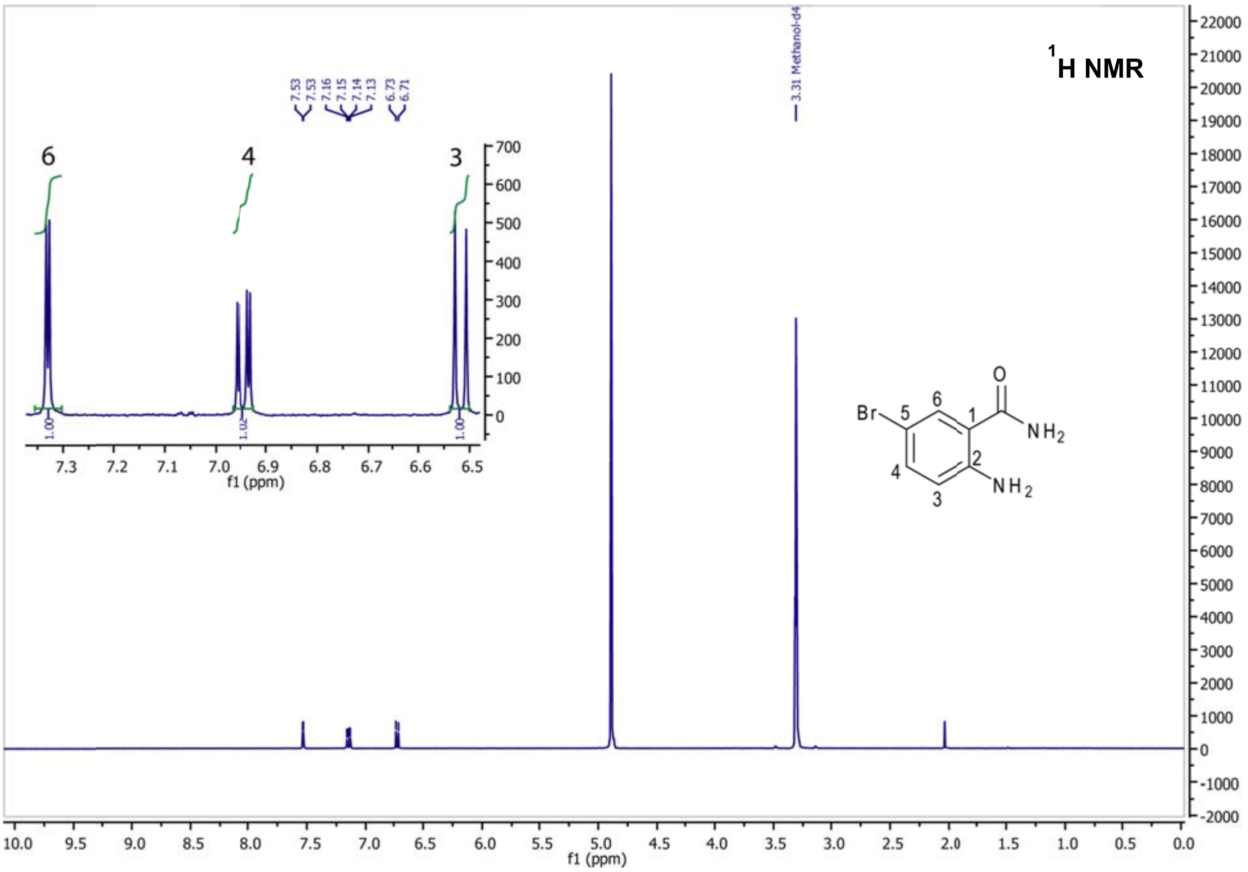

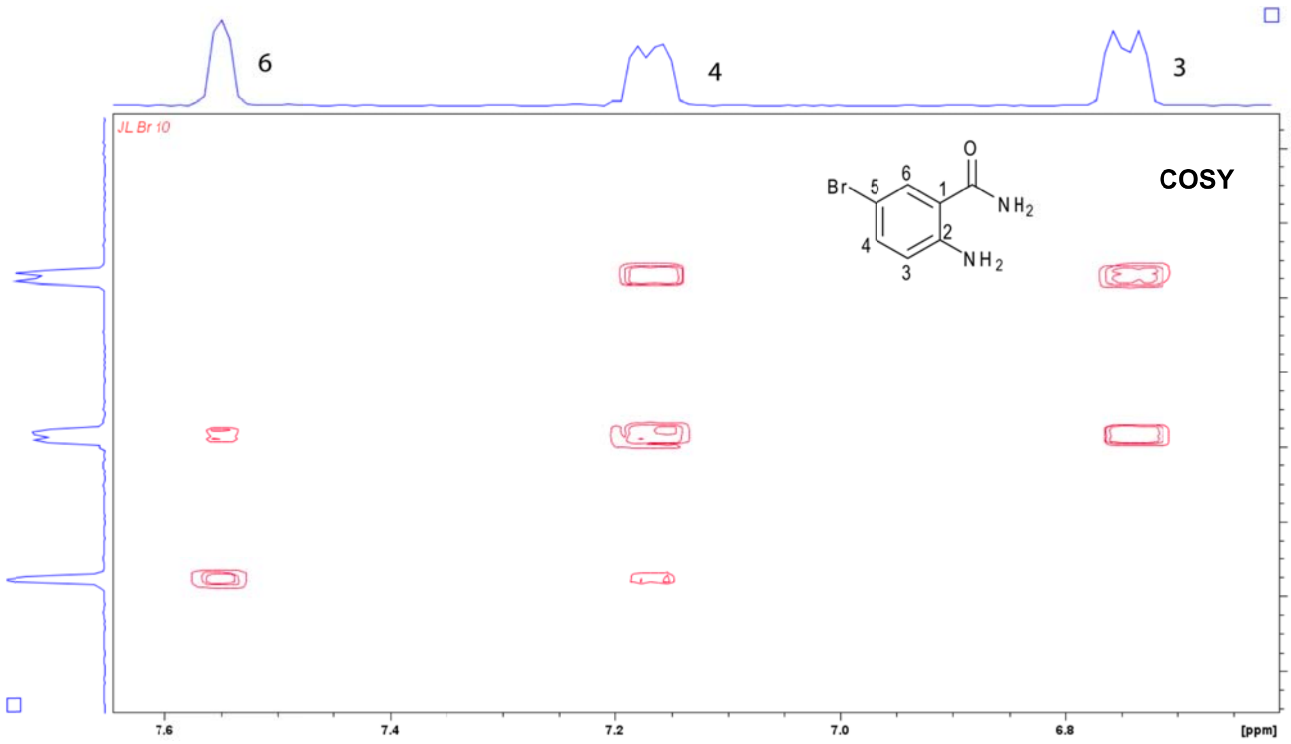

Supplementary Figure 14: ^{1}H NMR and COSY of 5-bromo anthranilamide (**2**).

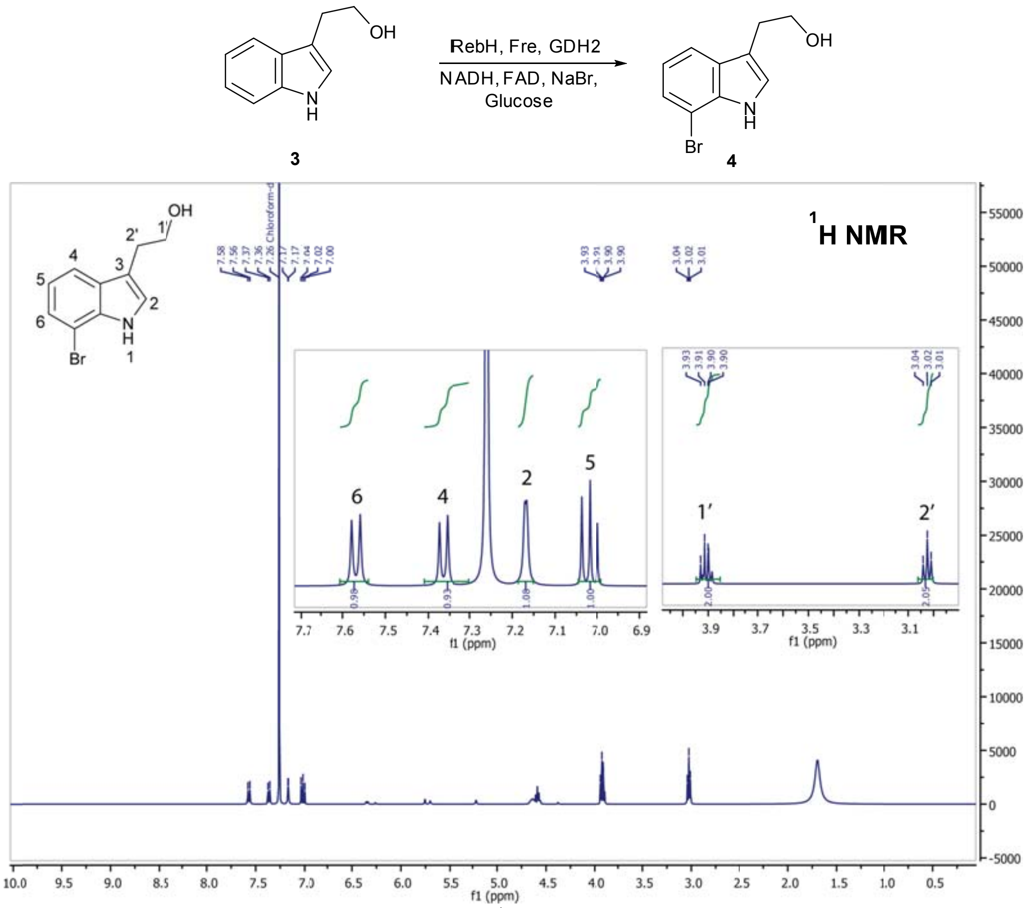

Supplementary Figure 15: ^{1}H NMR of 7-bromo tryptophol (**4**).

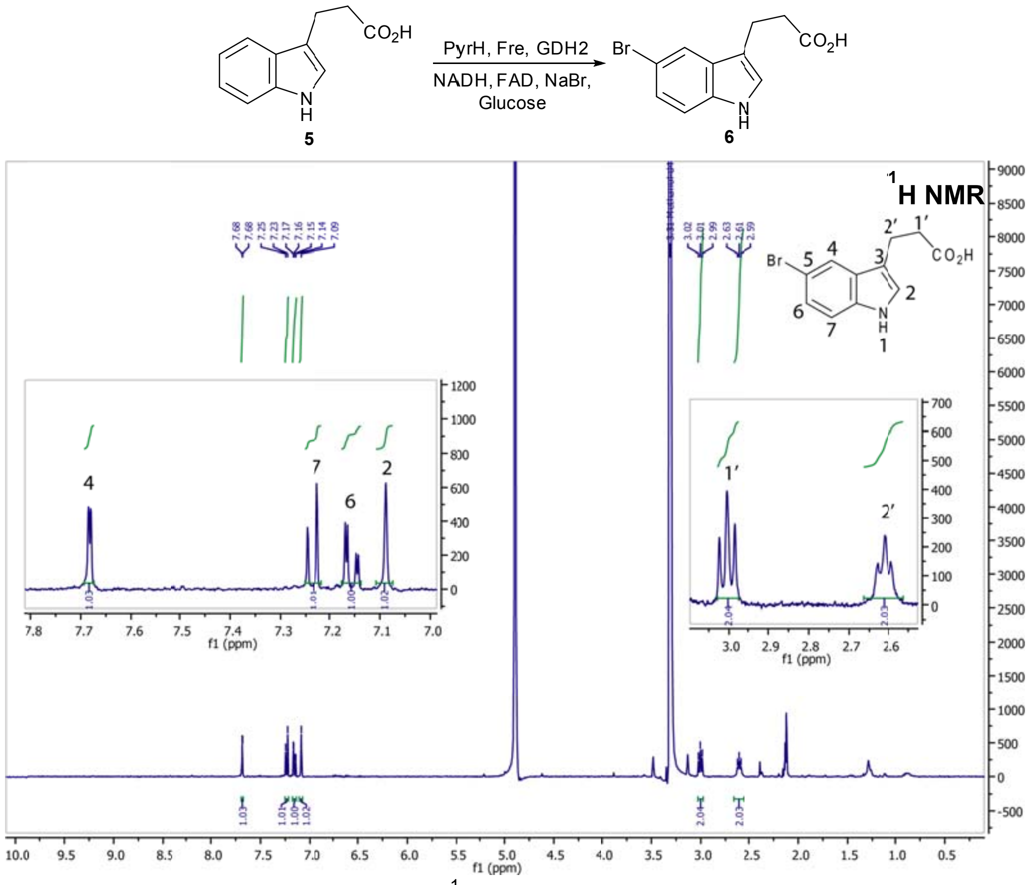

Supplementary Figure 16: ^{1}H NMR of 5-bromo indole-3-propinate (**6**).

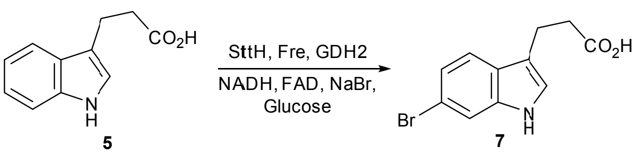

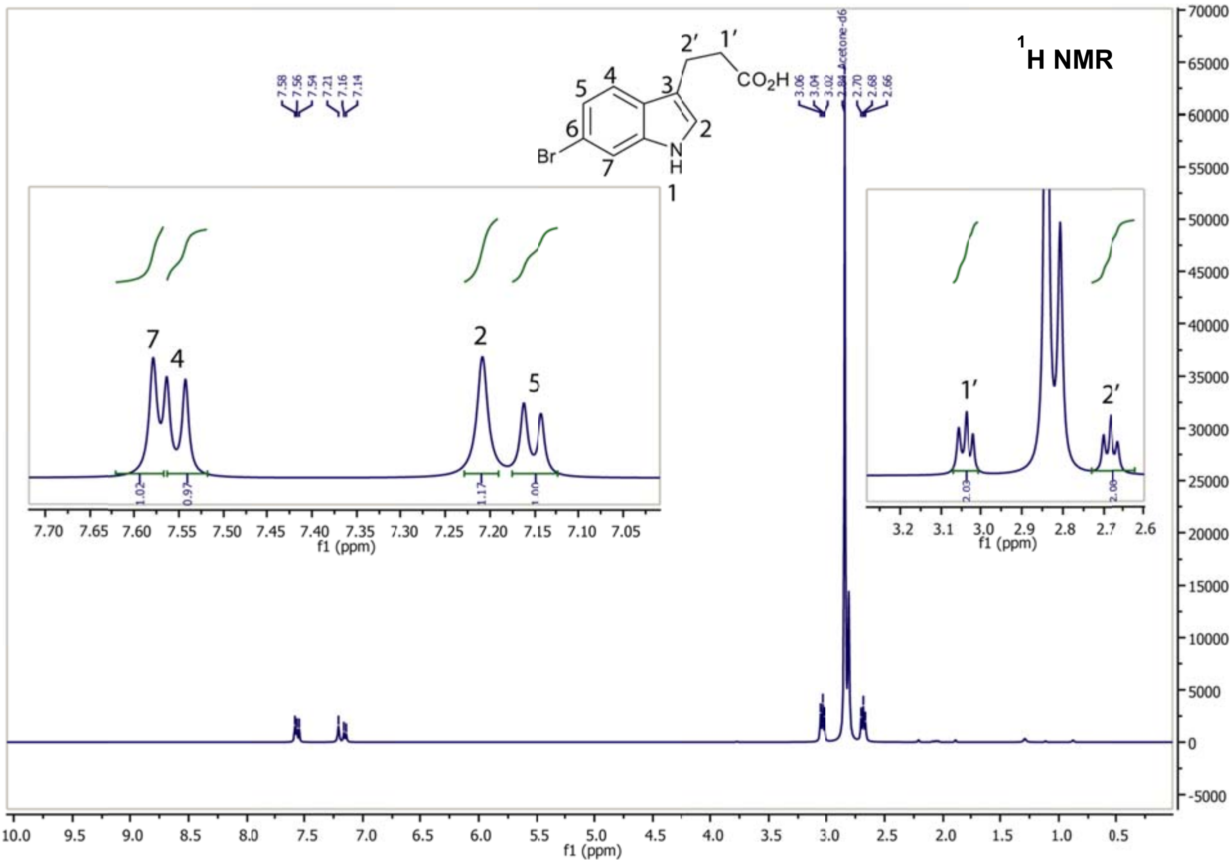

Supplementary Figure 17: ^{1}H NMR of 6-bromo indole-3-propinate (**7**).

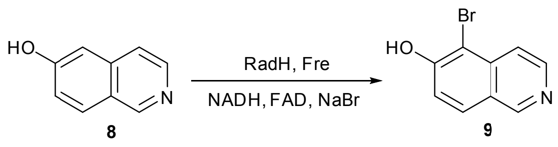

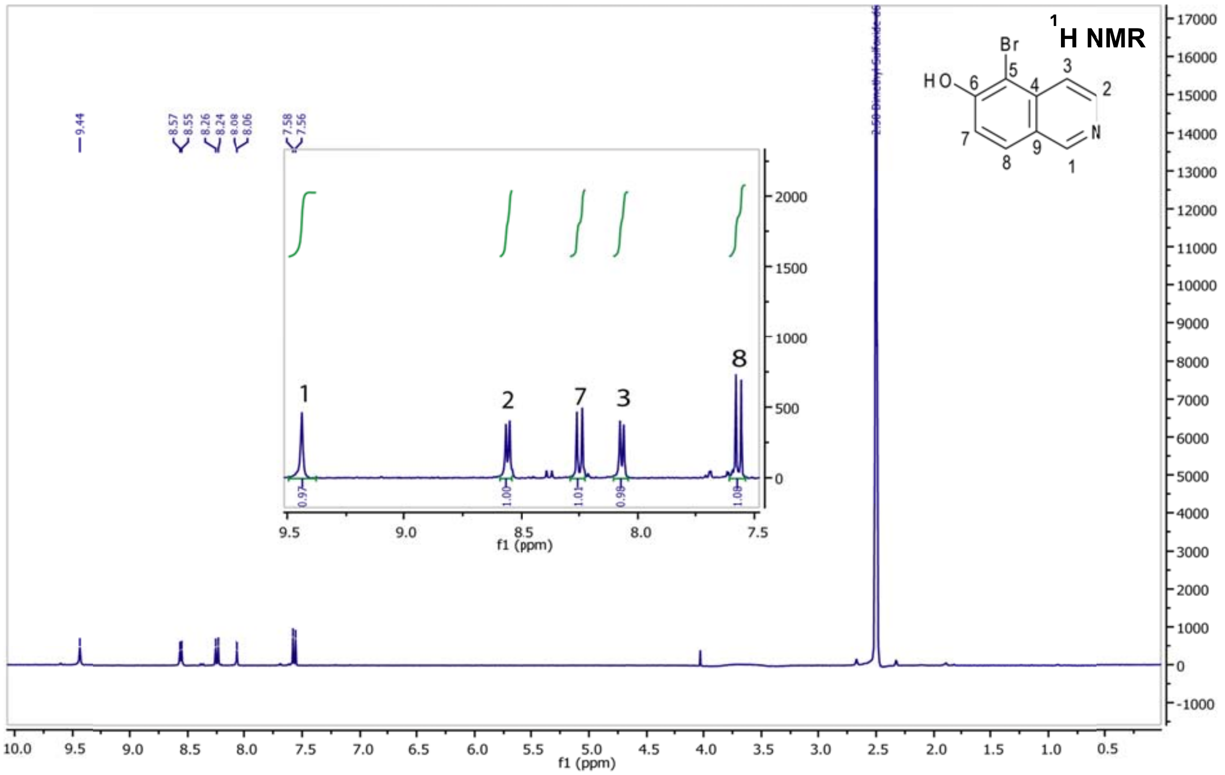

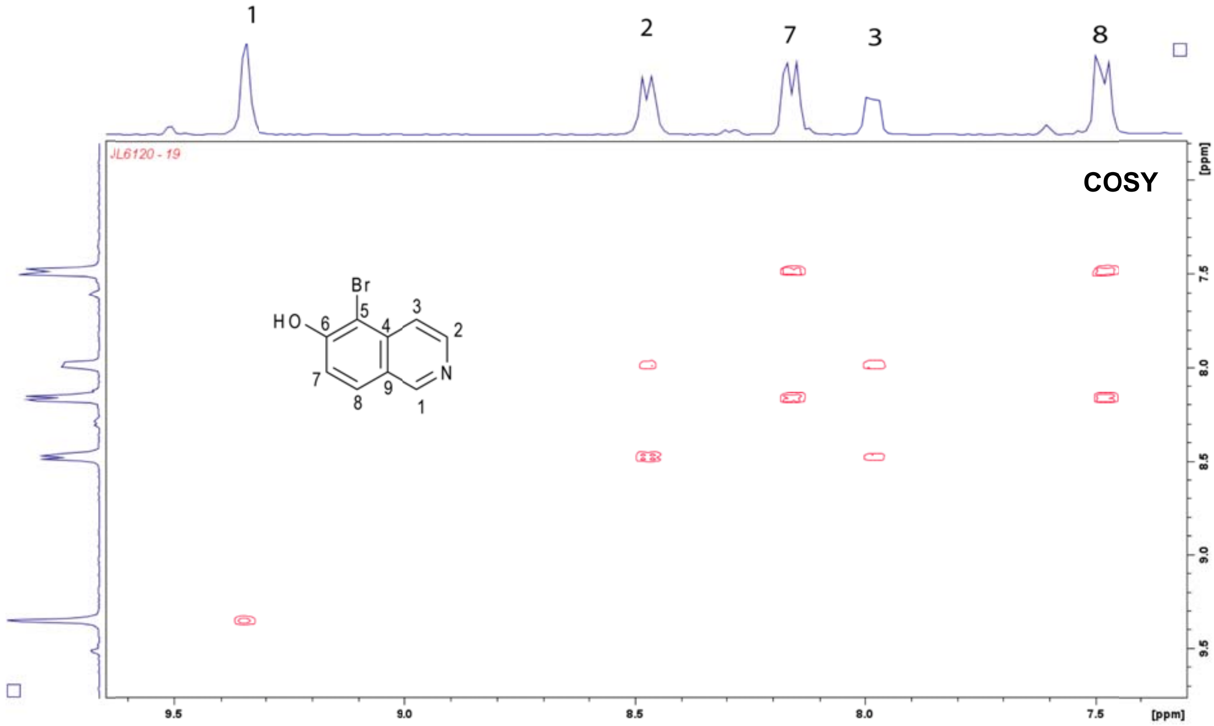

Supplementary Figure 18: ^{1}H NMR and COSY of 5-bromo-6-hydroxy isoquinoline (**9**).

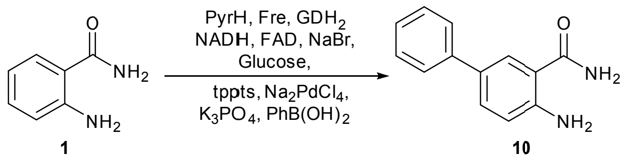

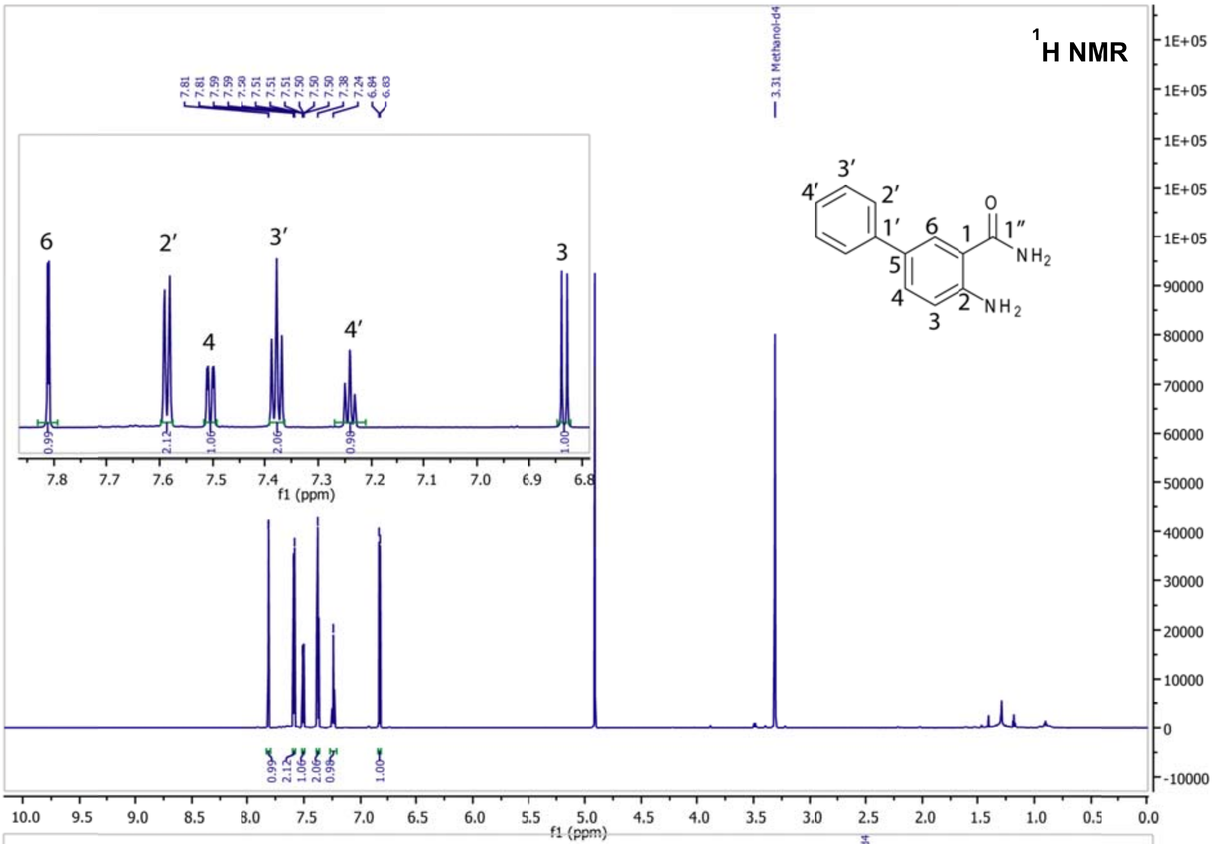

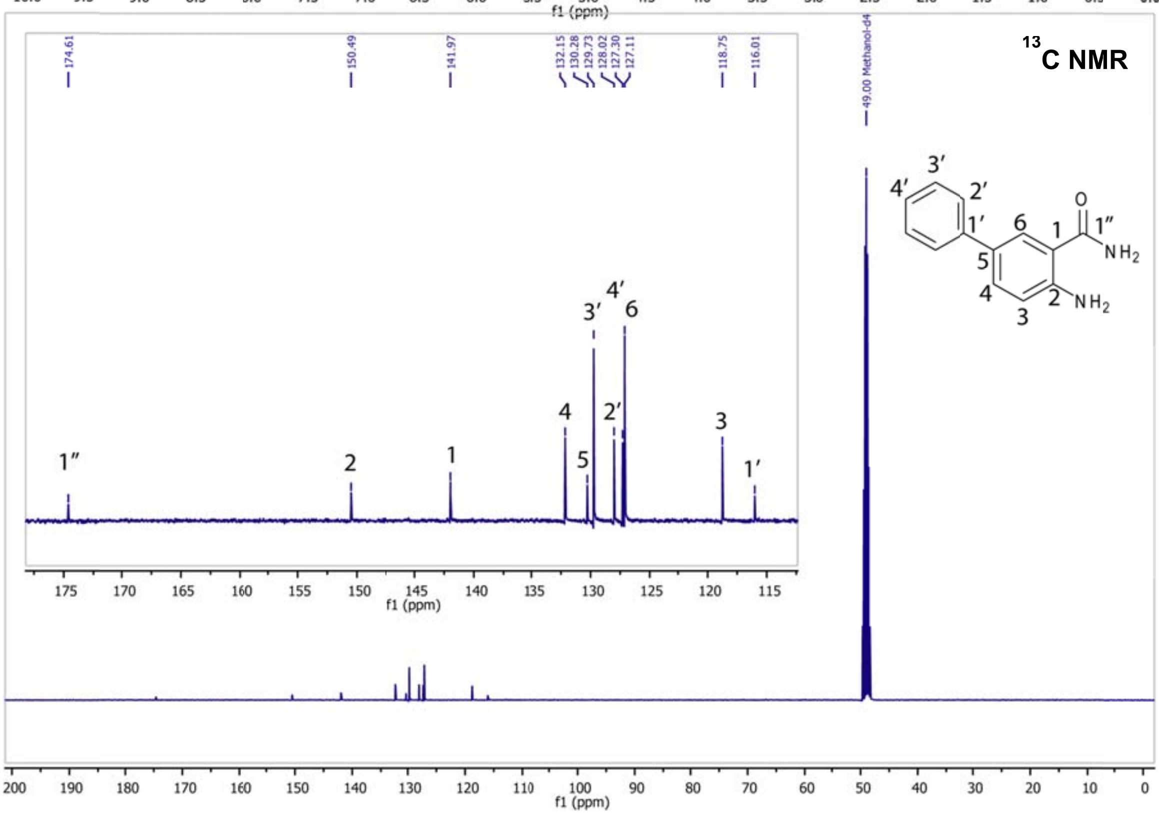

Supplementary Figure 19: ^{1}H NMR and ^{13}C NMR of 5-phenyl anthranilamide (**10**).

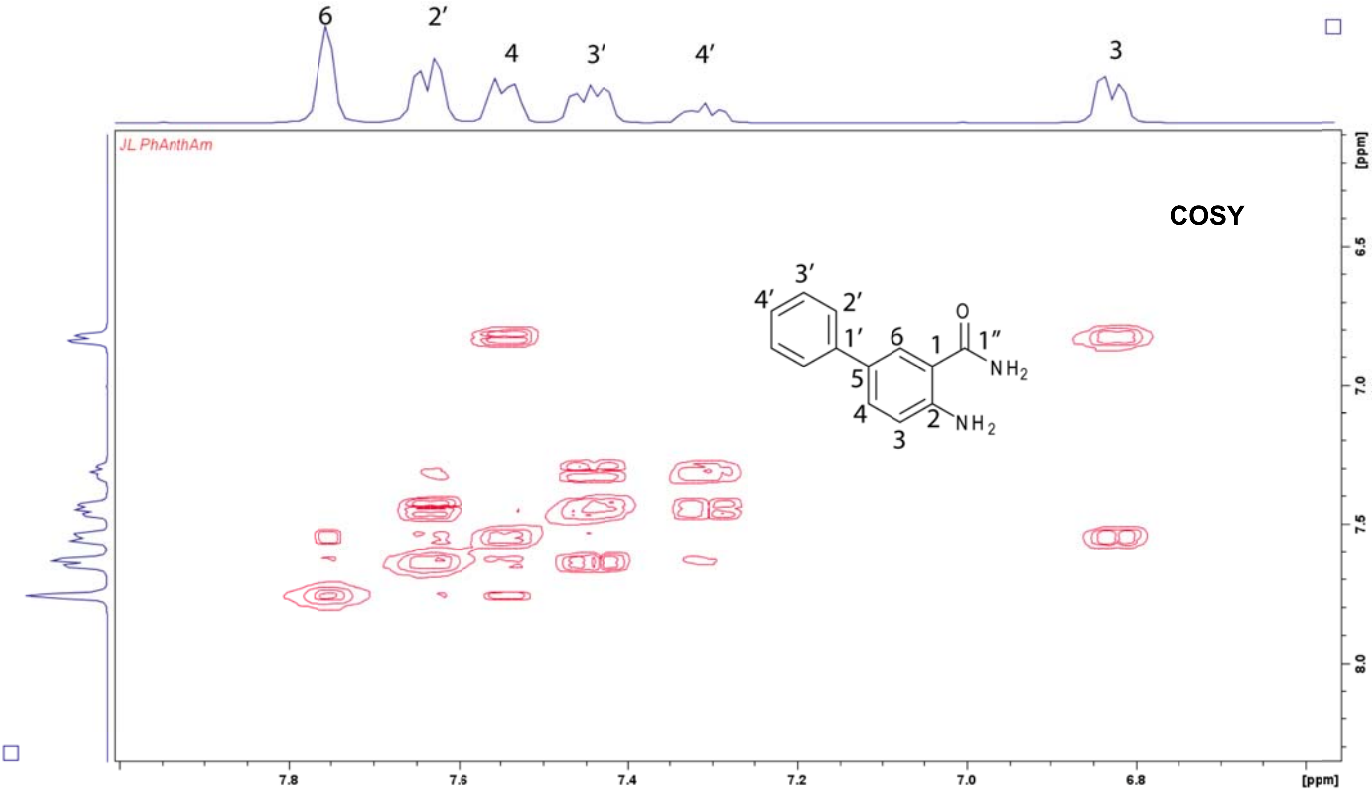

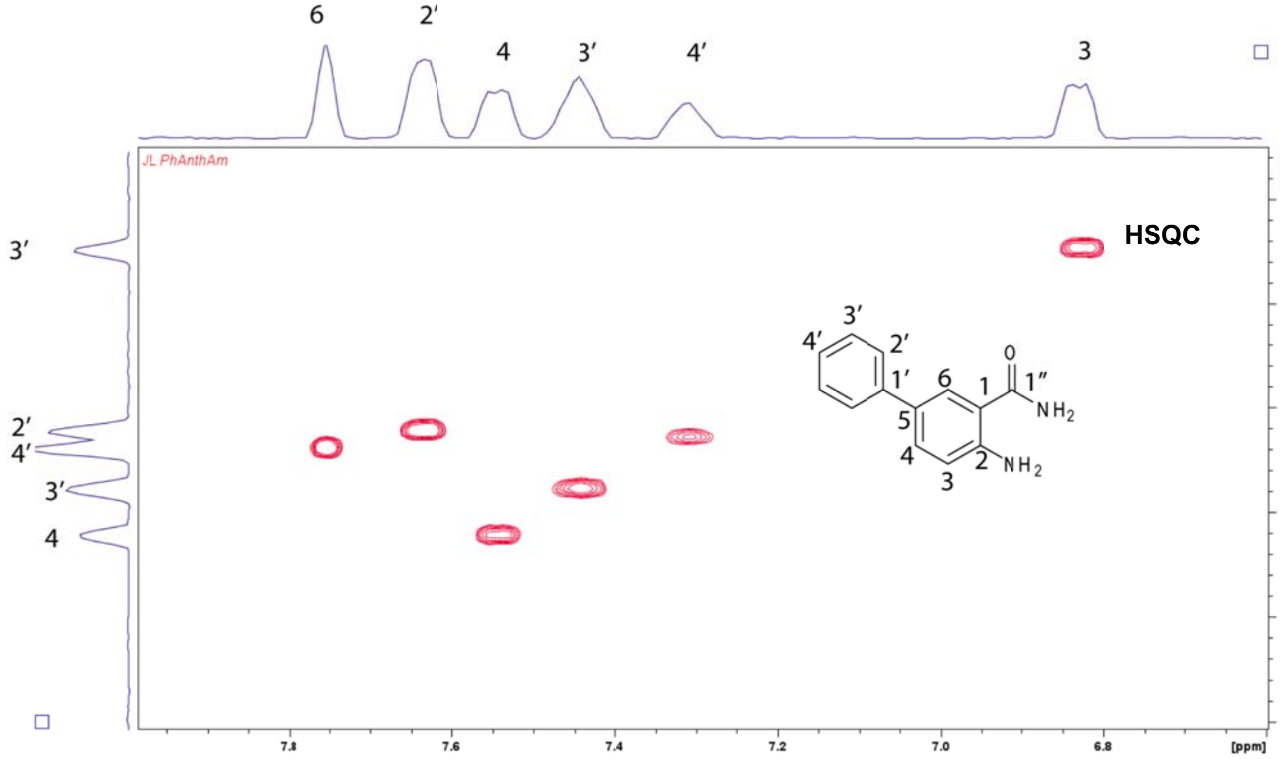

Supplementary Figure 20: COSY and HSQC of 5-phenyl anthranilamide (**10**).

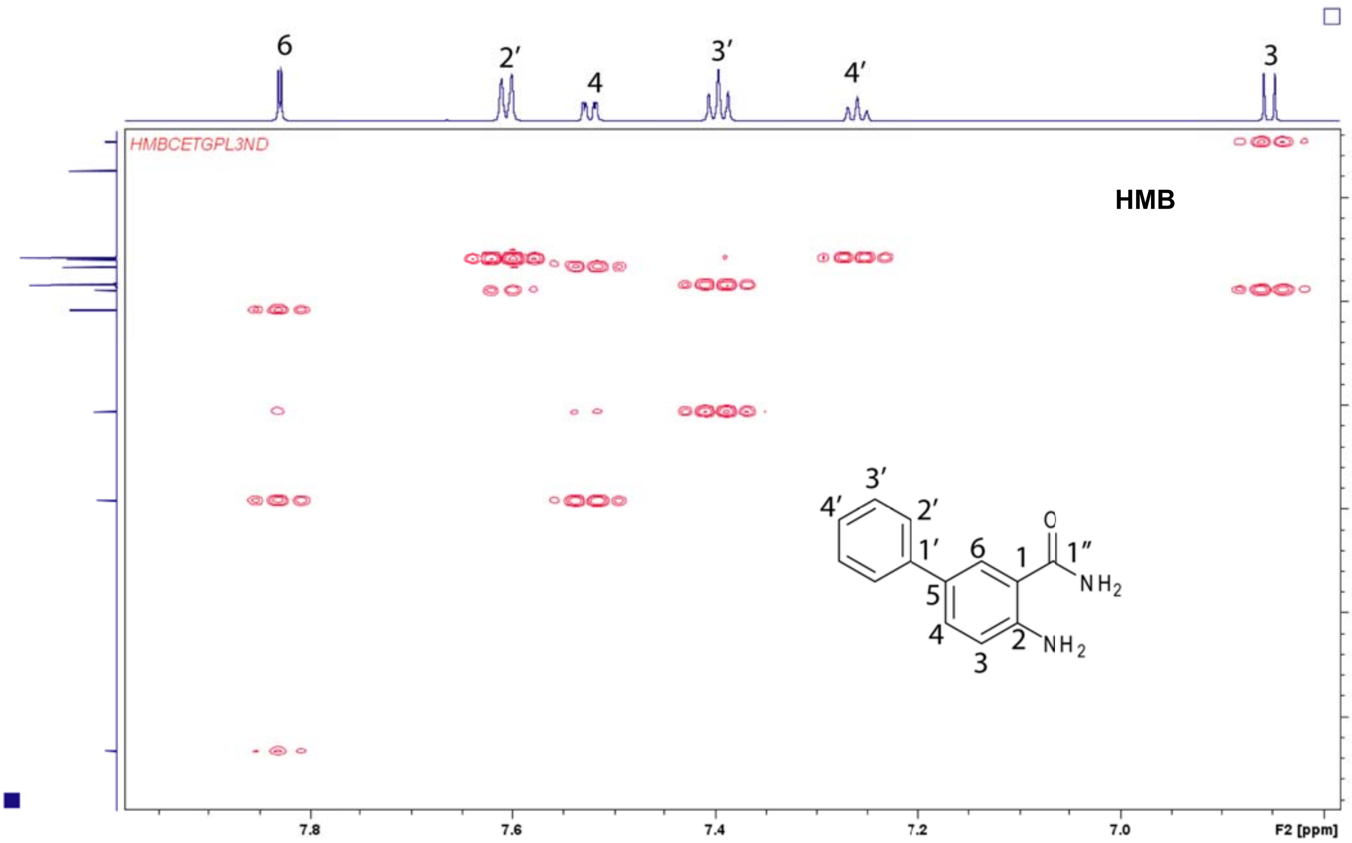

Supplementary Figure 21: HMBC of 5-phenyl anthranilamide (**10**).

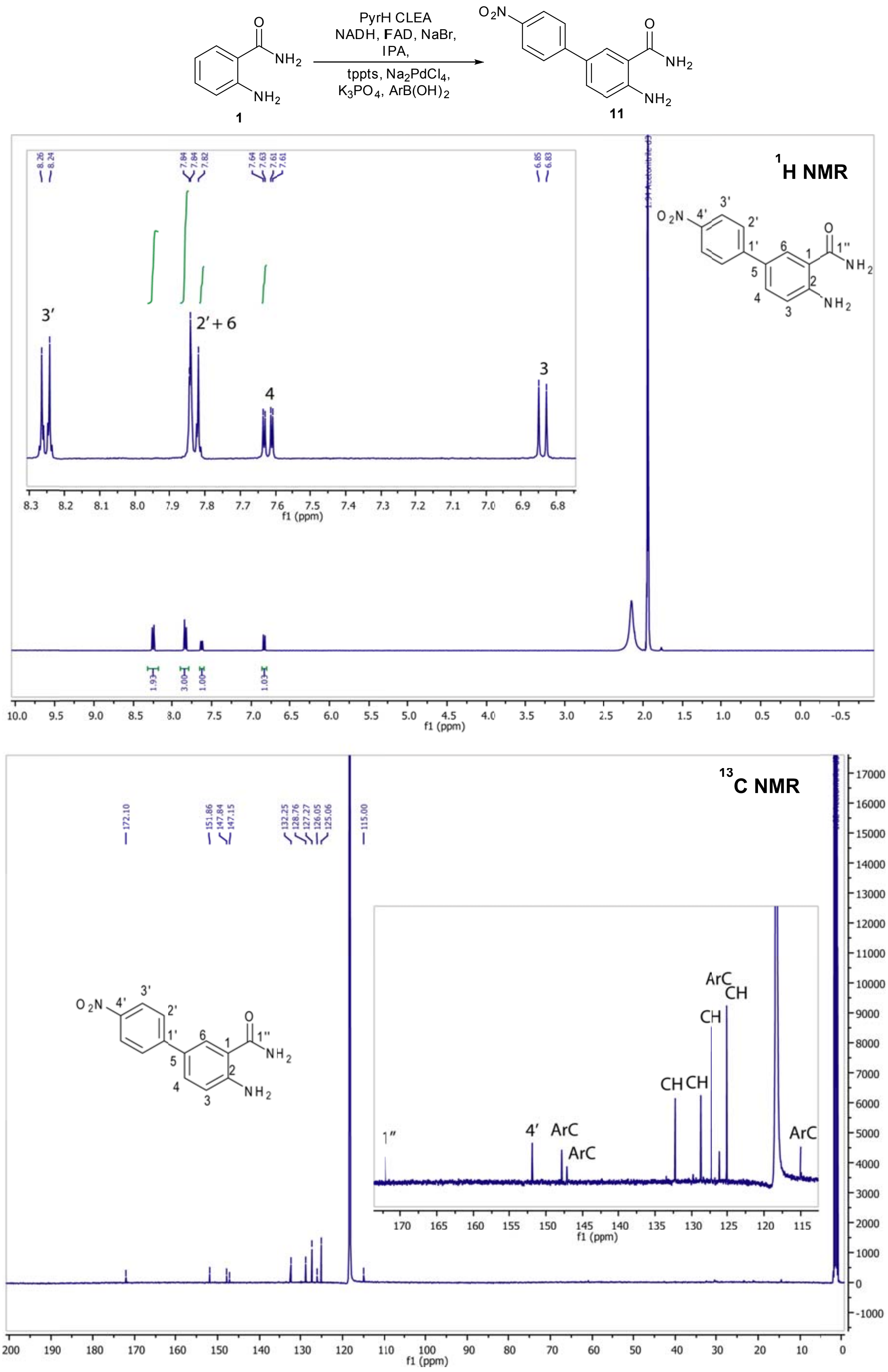

Supplementary Figure 22: ^{1}H NMR and ^{13}C NMR of 5-(4-nitrophenyl) anthranilamide (**11**).

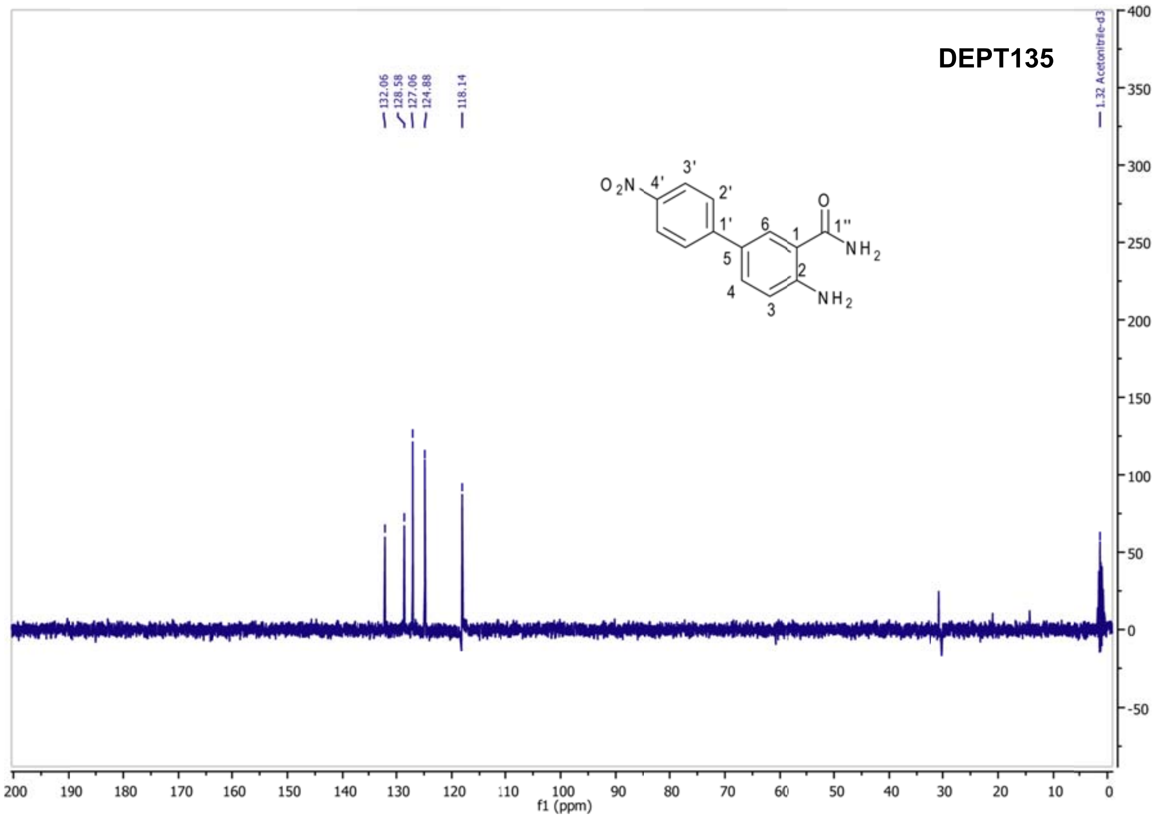

Supplementary Figure 23: DEPT135 of 5-(4-nitrophenyl) anthranilamide (**11**).

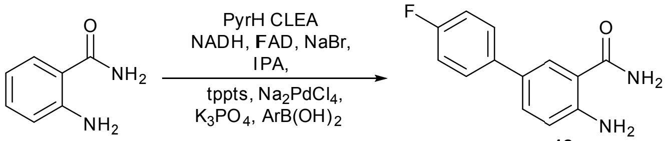

1 12

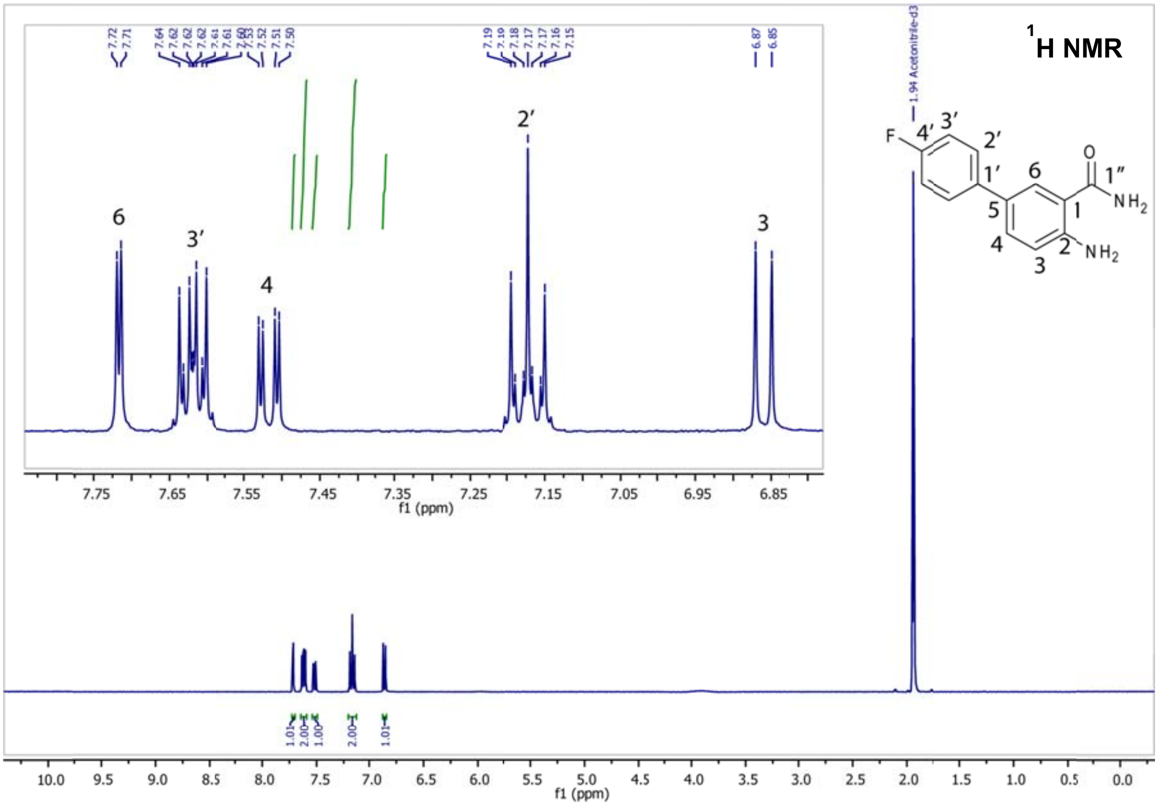

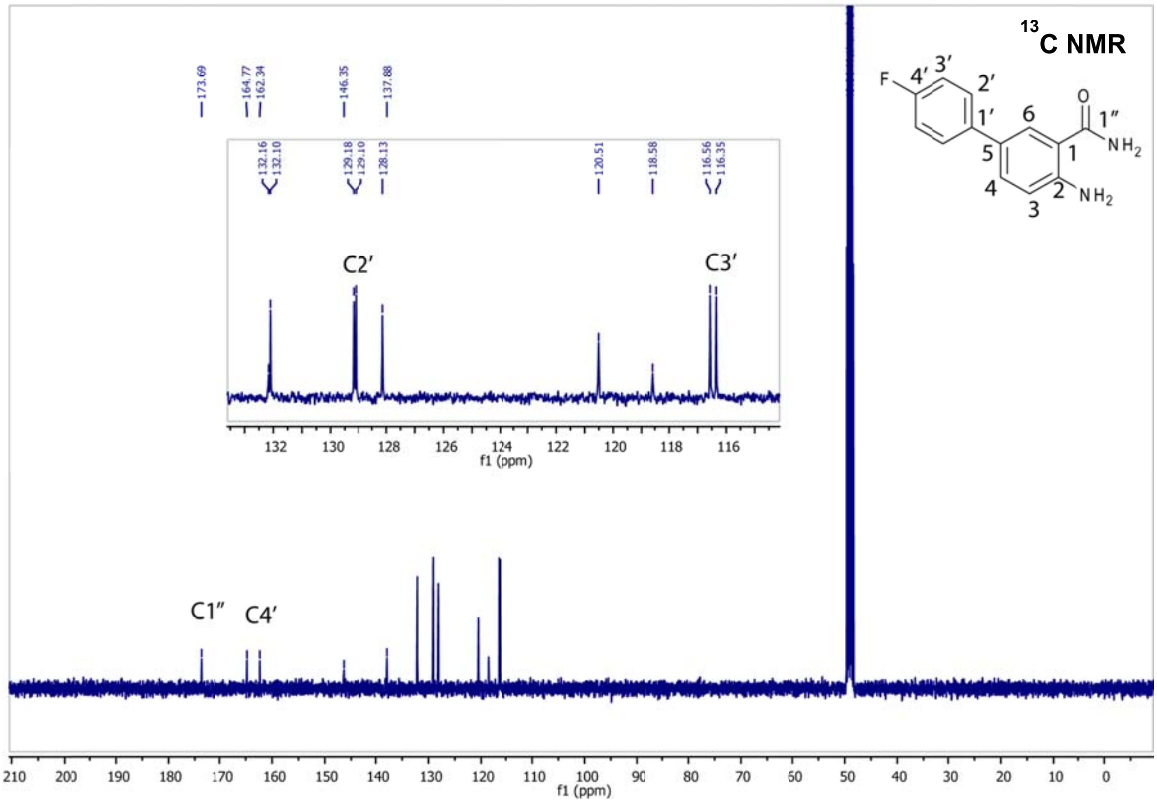

Supplementary Figure 24: ^{1}H NMR and ^{13}C NMR of 5-(4-fluorophenyl) anthranilamide (**12**).

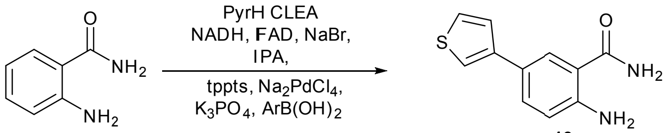

1 13

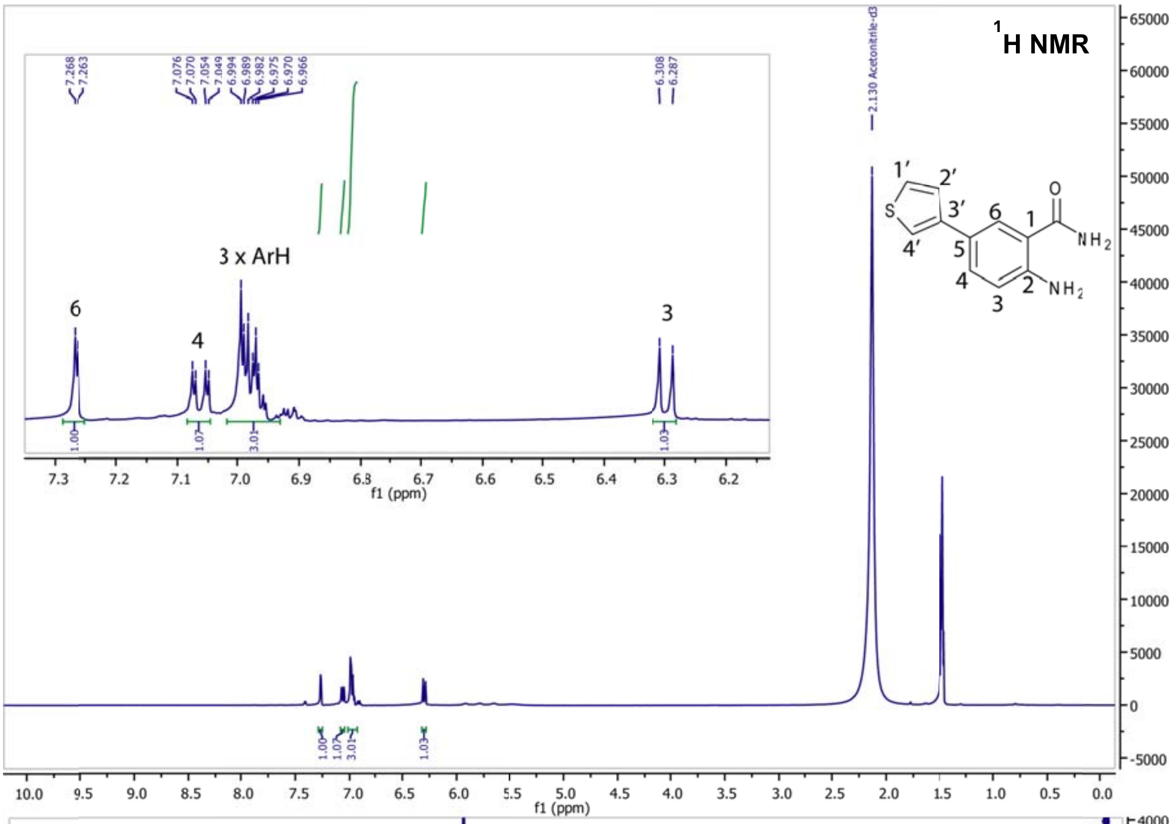

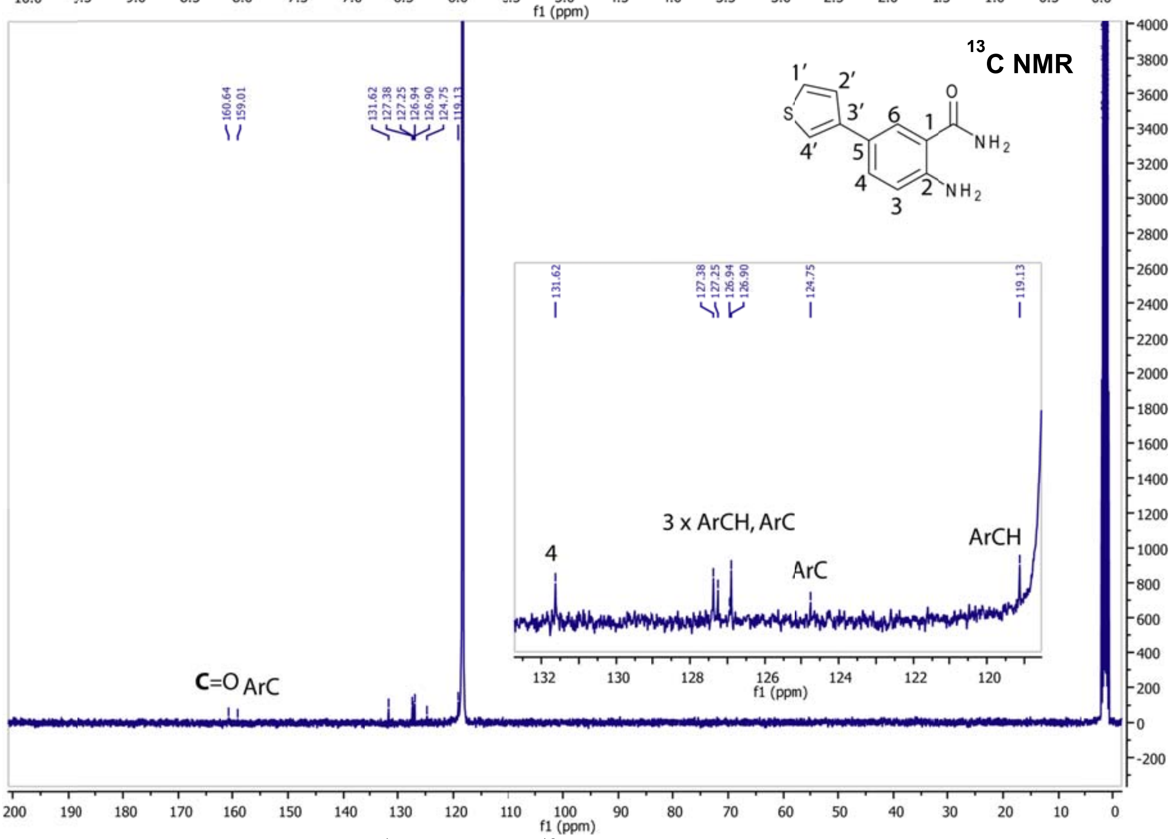

Supplementary Figure 25: ^{1}H NMR and ^{13}C NMR of 5-(thiophen-3-yl) anthranilamide (**13**).

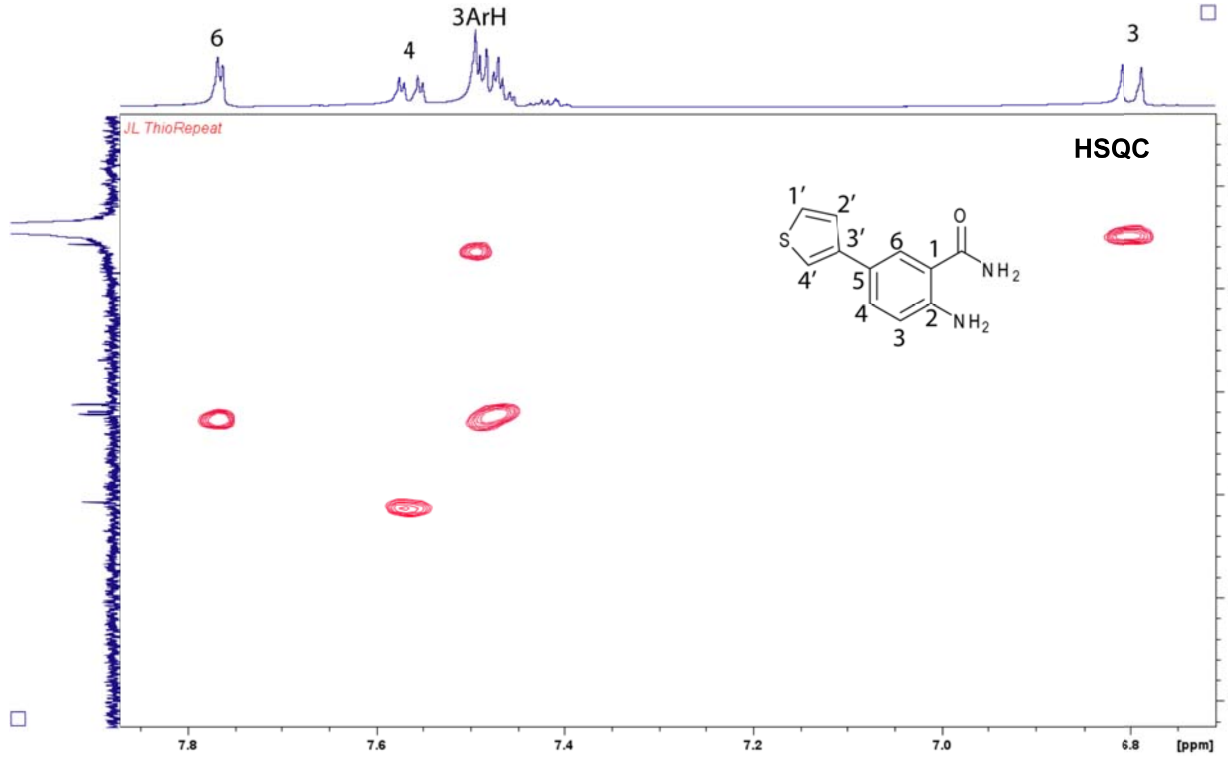

Supplementary Figure 26: HSQC of 5-(thiophen-3-yl) anthranilamide (**13**).

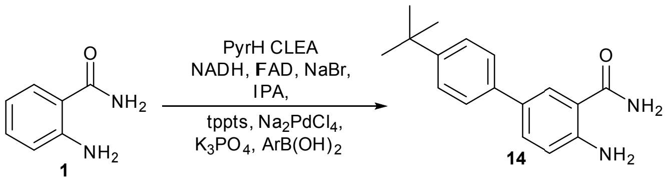

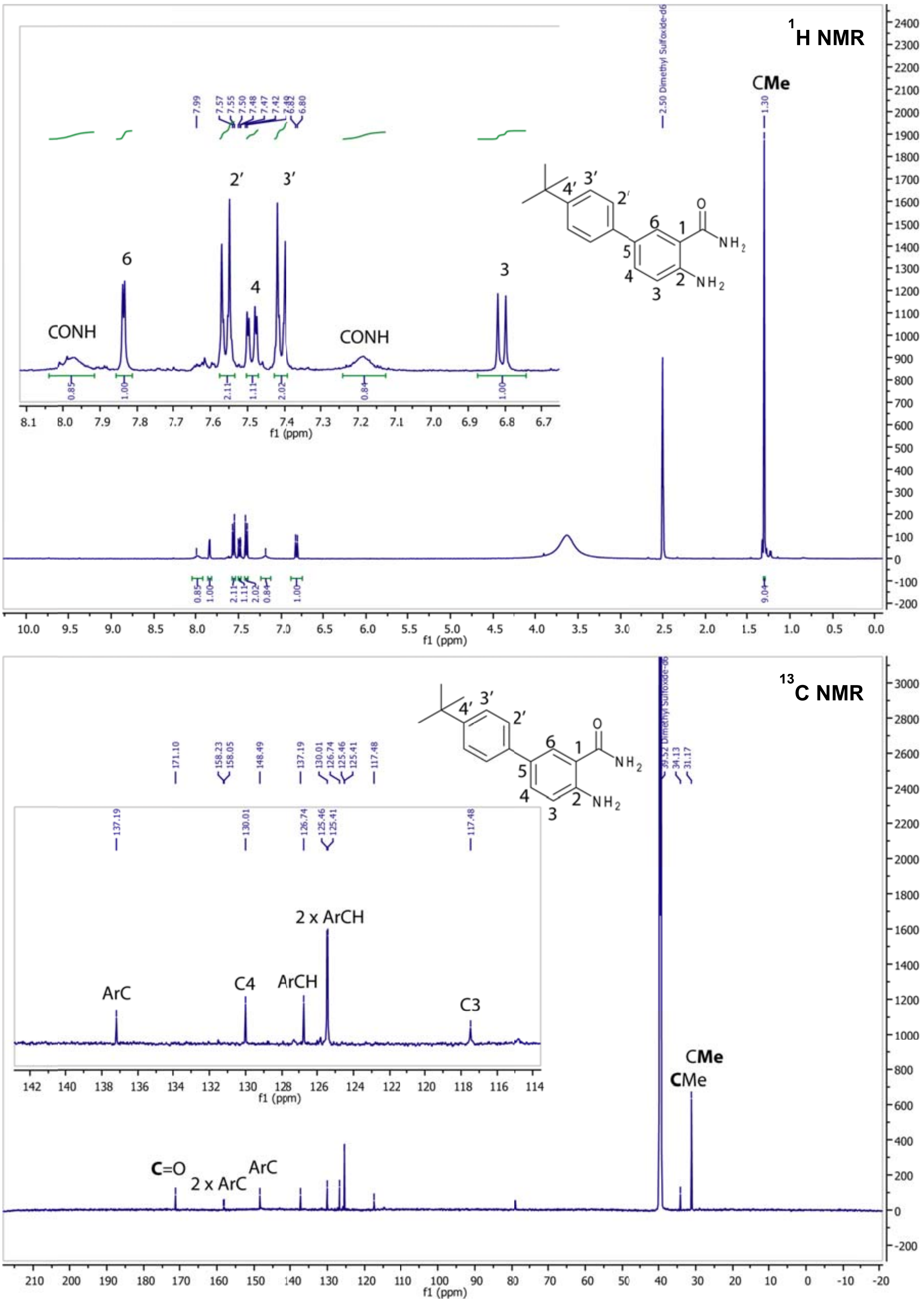

Supplementary Figure 27: ^{1}H and ^{13}C NMR of 5-(4-terbutyl) anthranilamide (**14**).

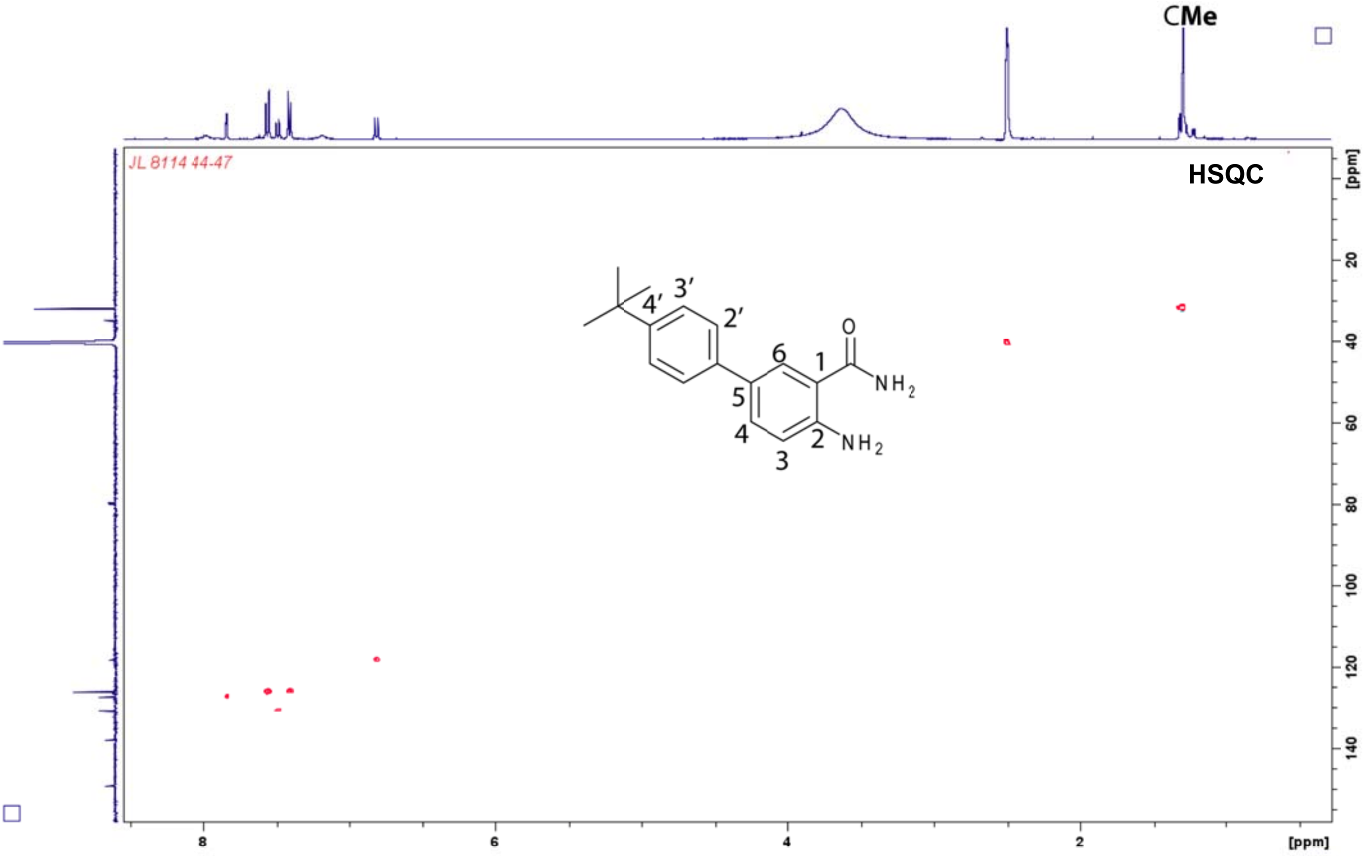

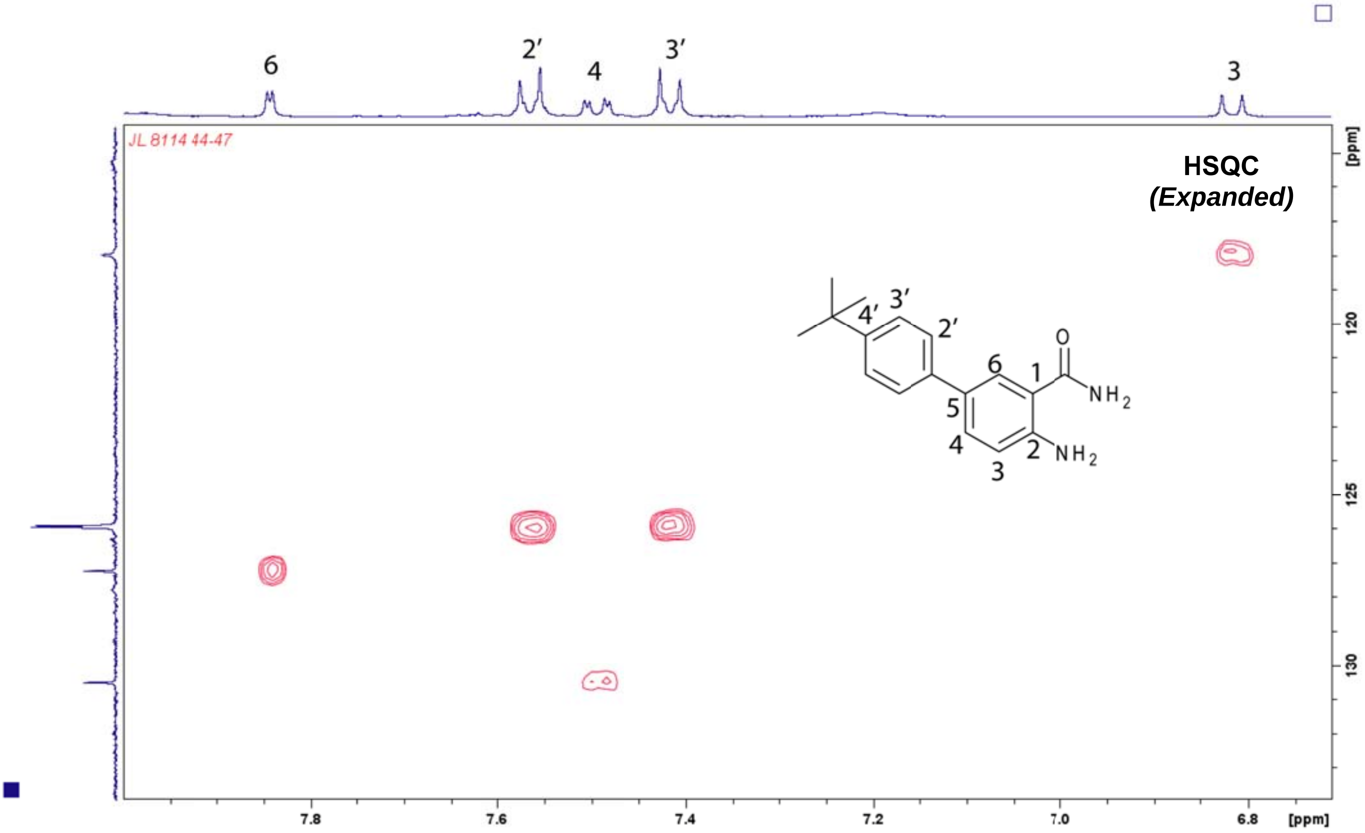

Supplementary Figure 28: HSQC of 5-(4-terbutyl) anthranilamide (**14**).

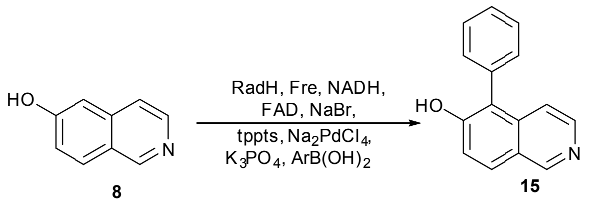

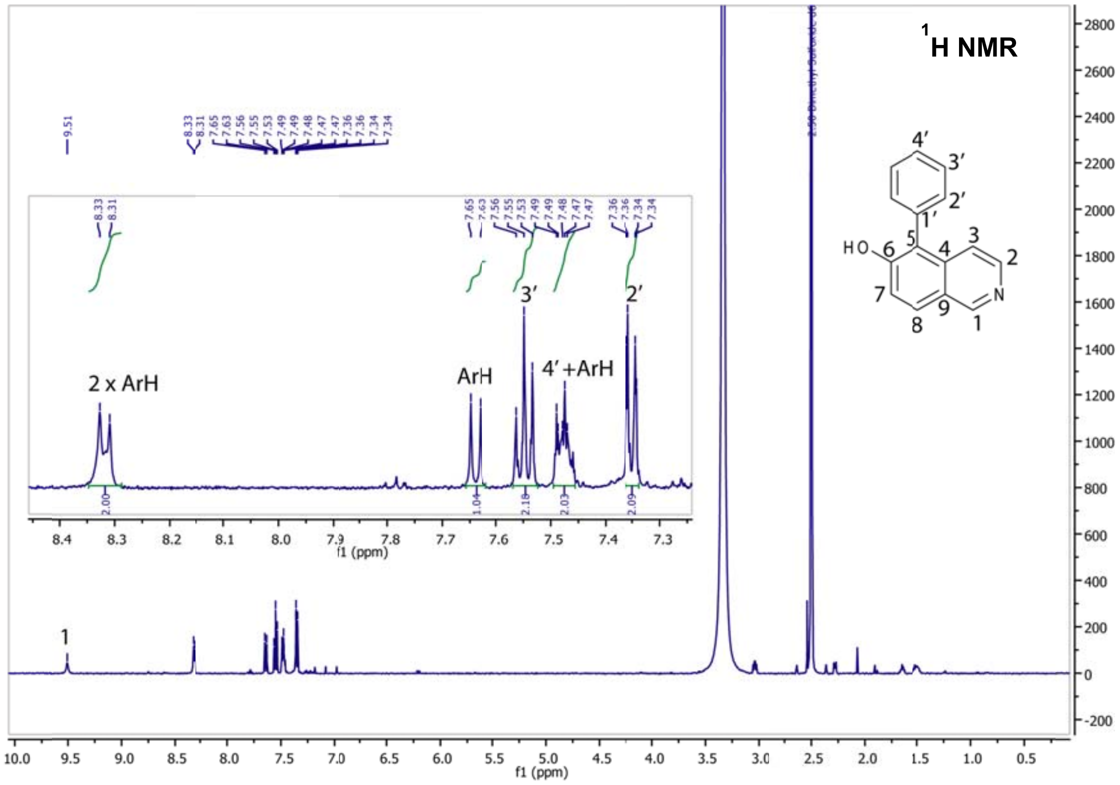

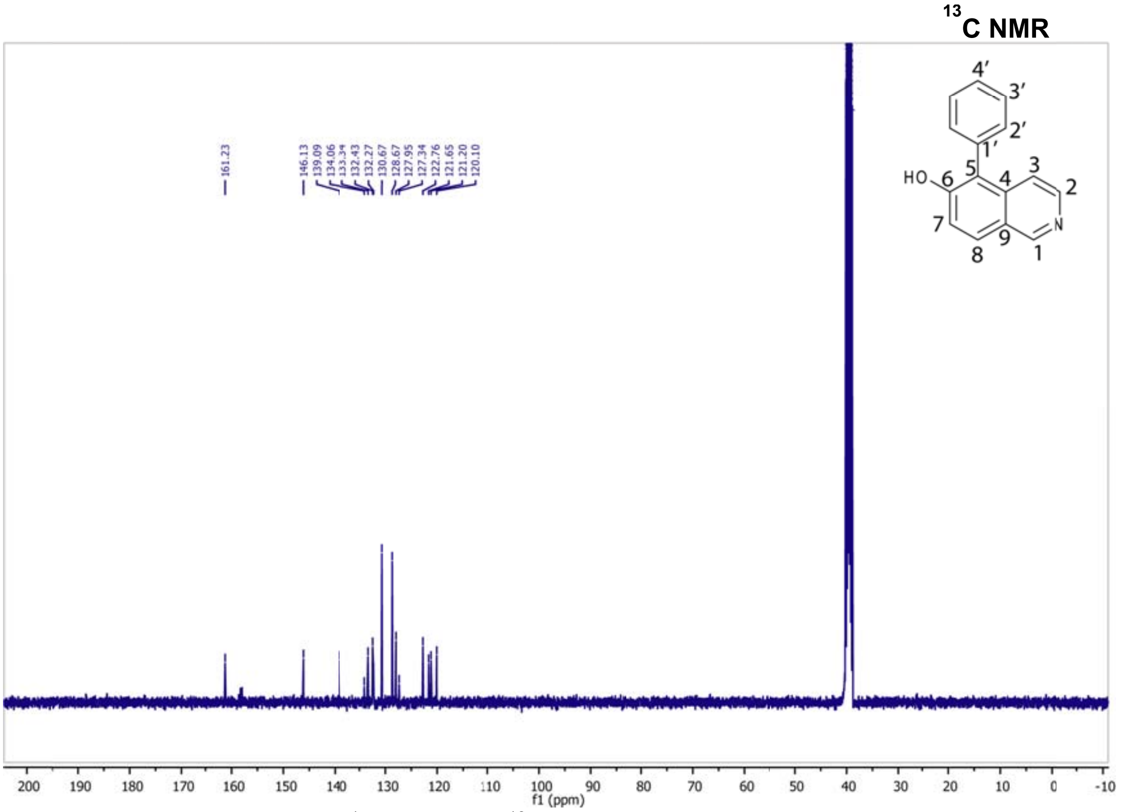

Supplementary Figure 29: ^{1}H NMR and ^{13}C NMR of 5-phenyl-6-hydroxy isoquinqoline (**15**).

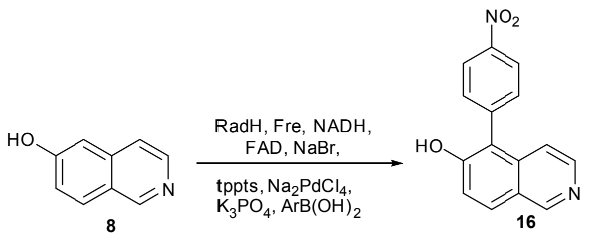

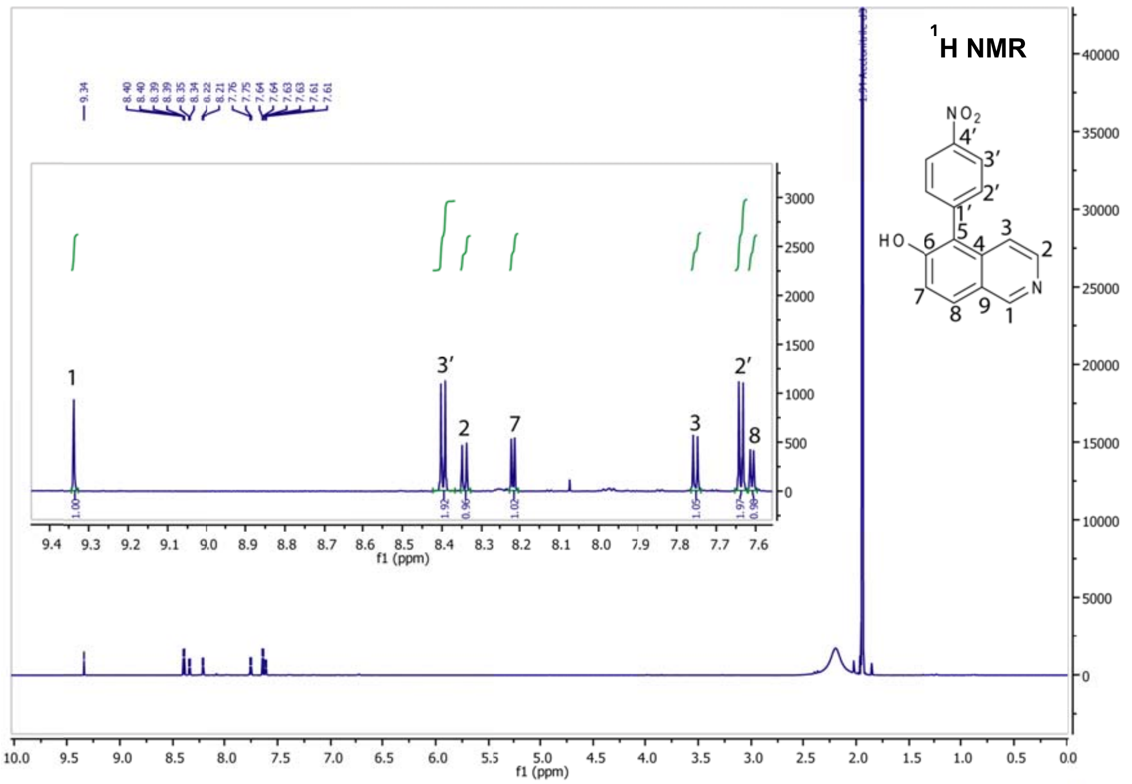

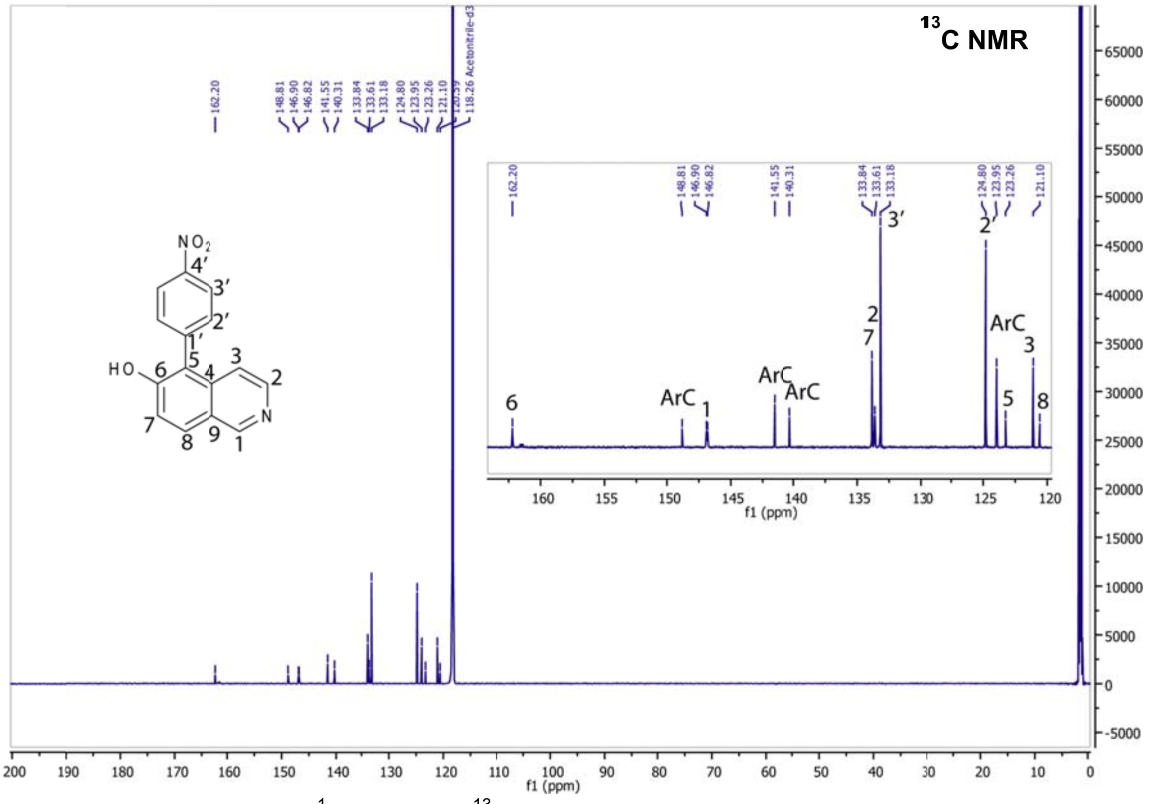

Supplementary Figure 30: ^{1}H NMR and ^{13}C NMR of 5-(4-nitrophenyl)-6-hydroxy isoquinqoline (**16**).

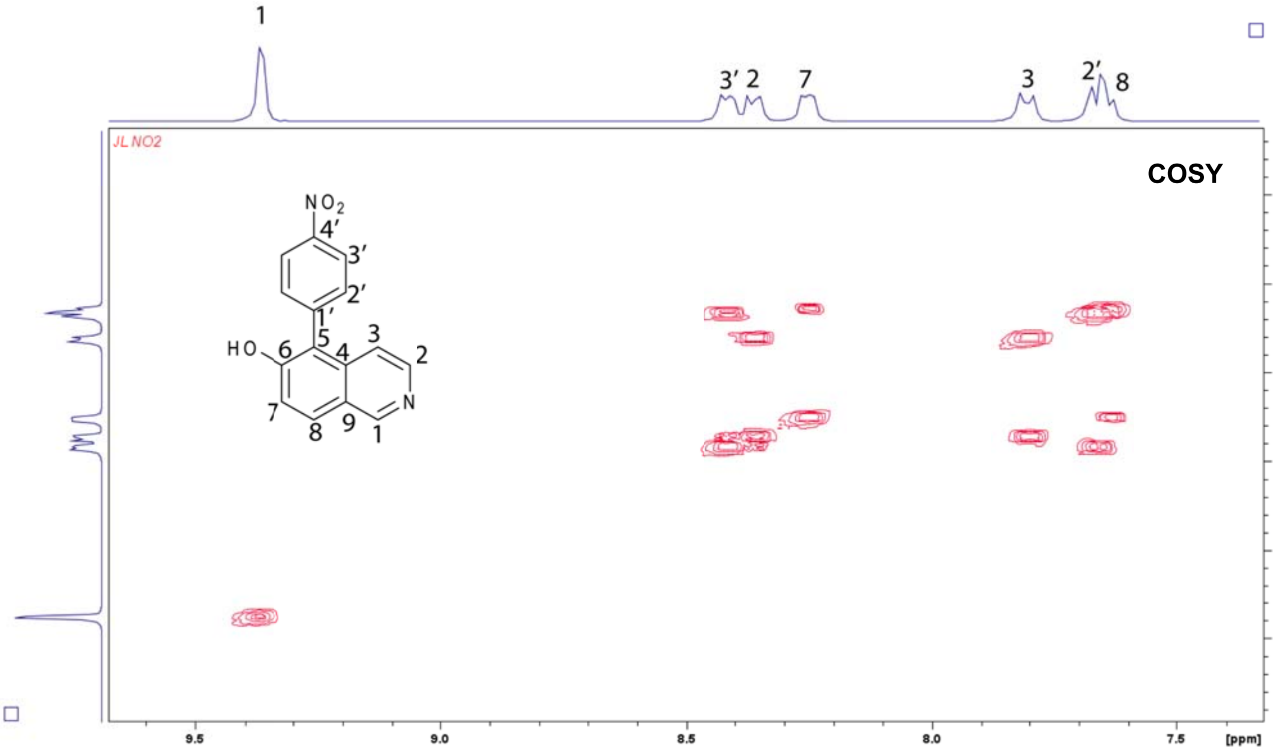

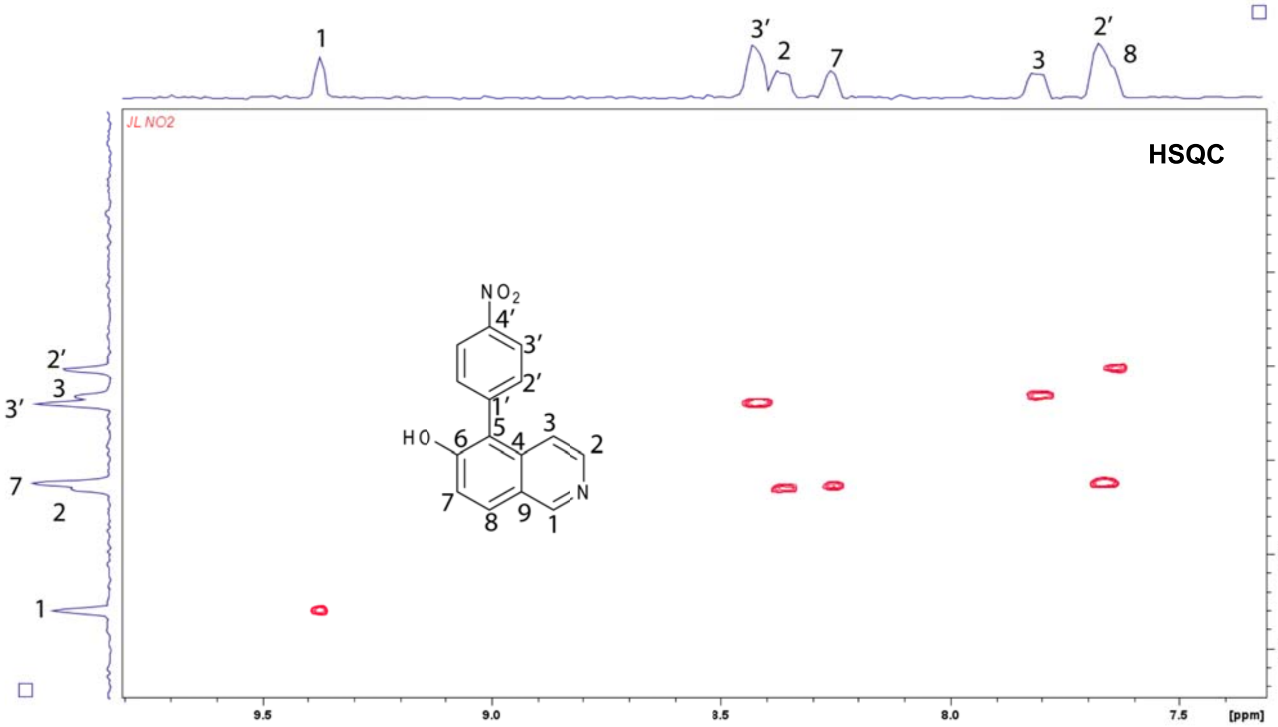

Supplementary Figure 31: COSY and HSQC of 5-(4-nitrophenyl)-6-hydroxy isoquinqoline (**16**).

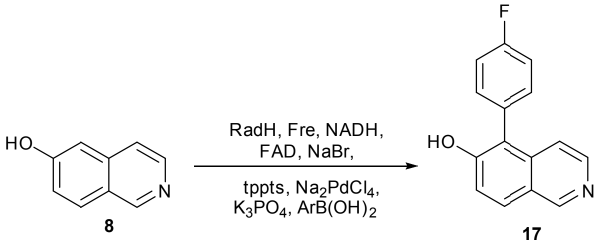

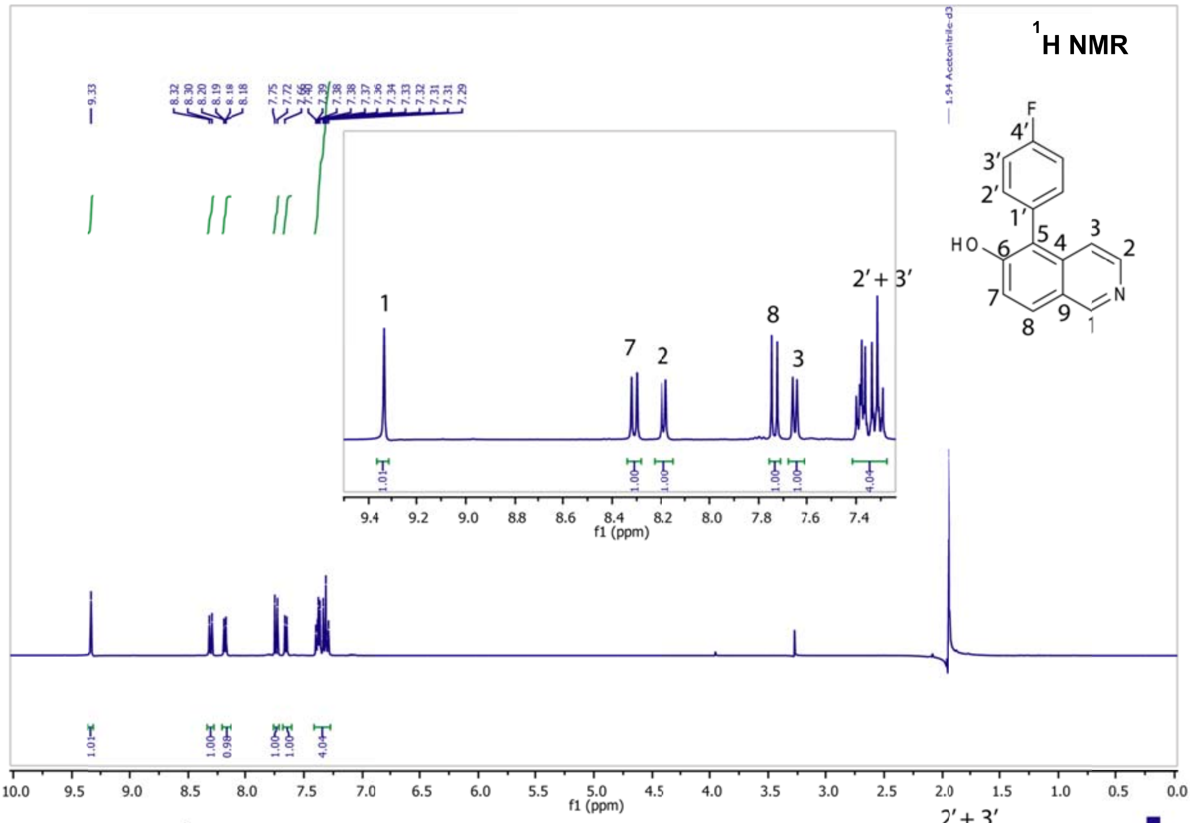

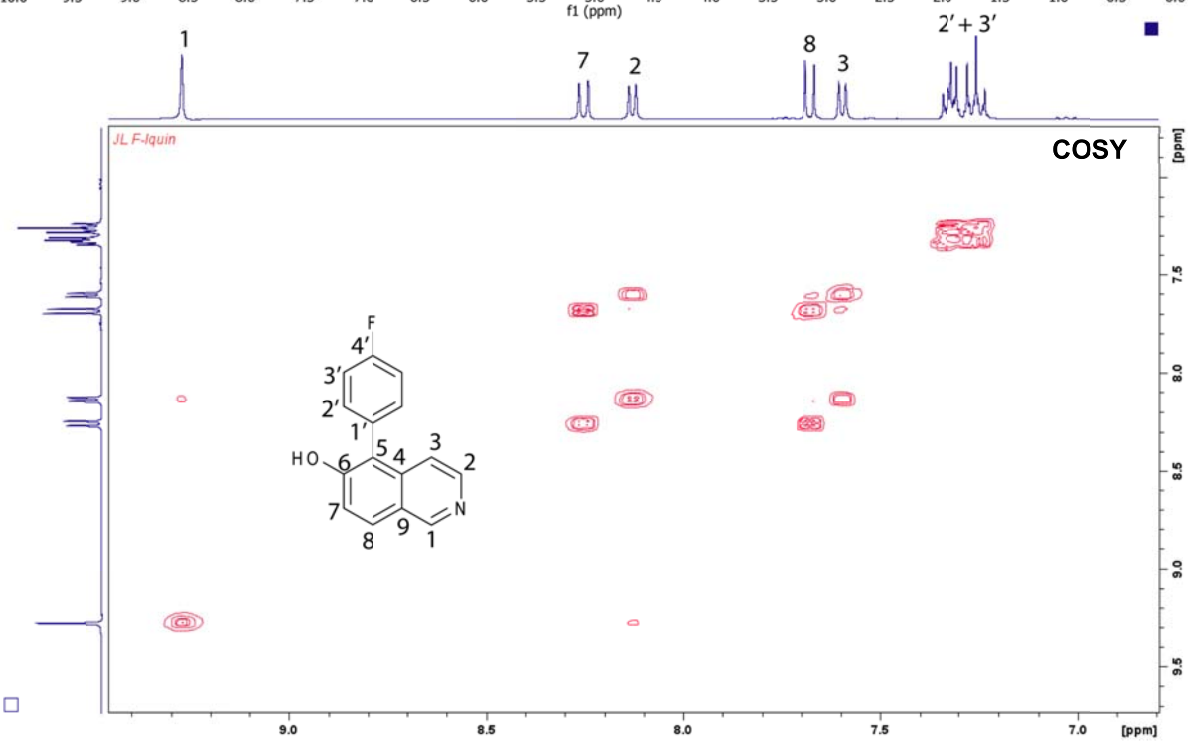

Supplementary Figure 32: ^{1}H NMR and COSY of 5-(4-flurophenyl)-6-hydroxy isoquinqoline (**17**).

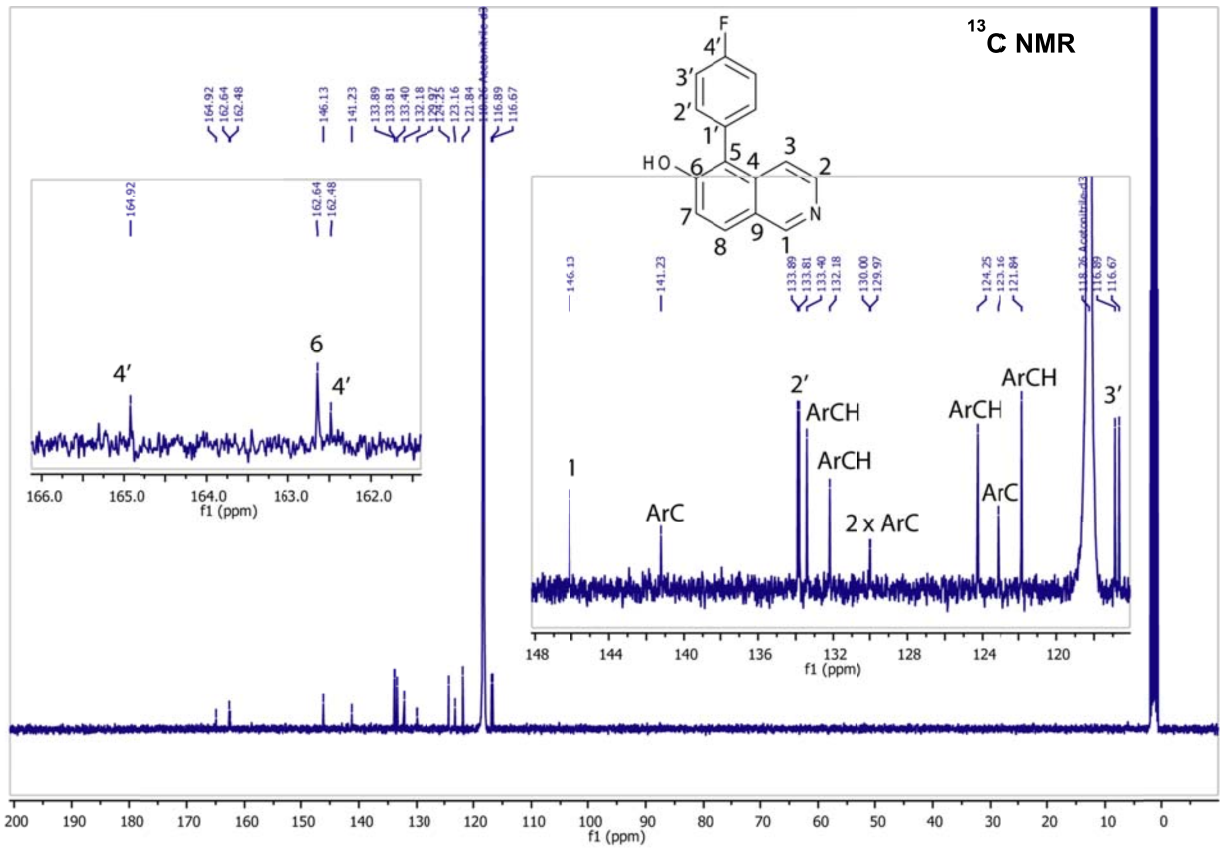

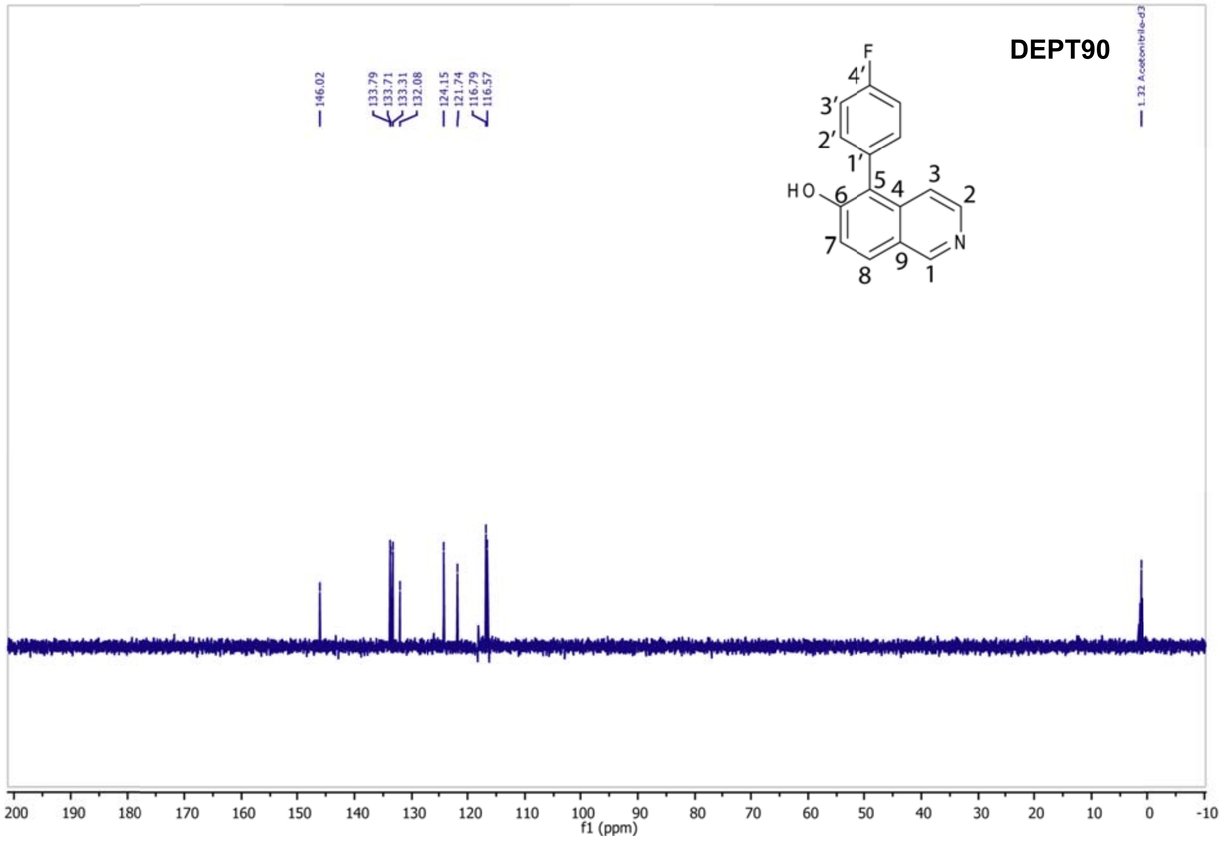

Supplementary Figure 33: ^{13}C NMR and DEPT90 of 5-(4-flurophenyl)-6-hydroxy isoquinqoline (**17**).

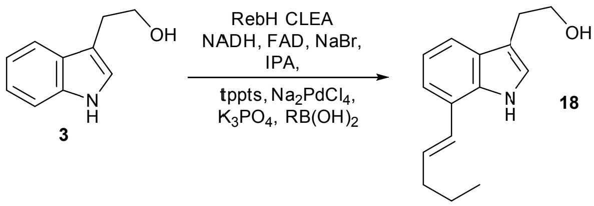

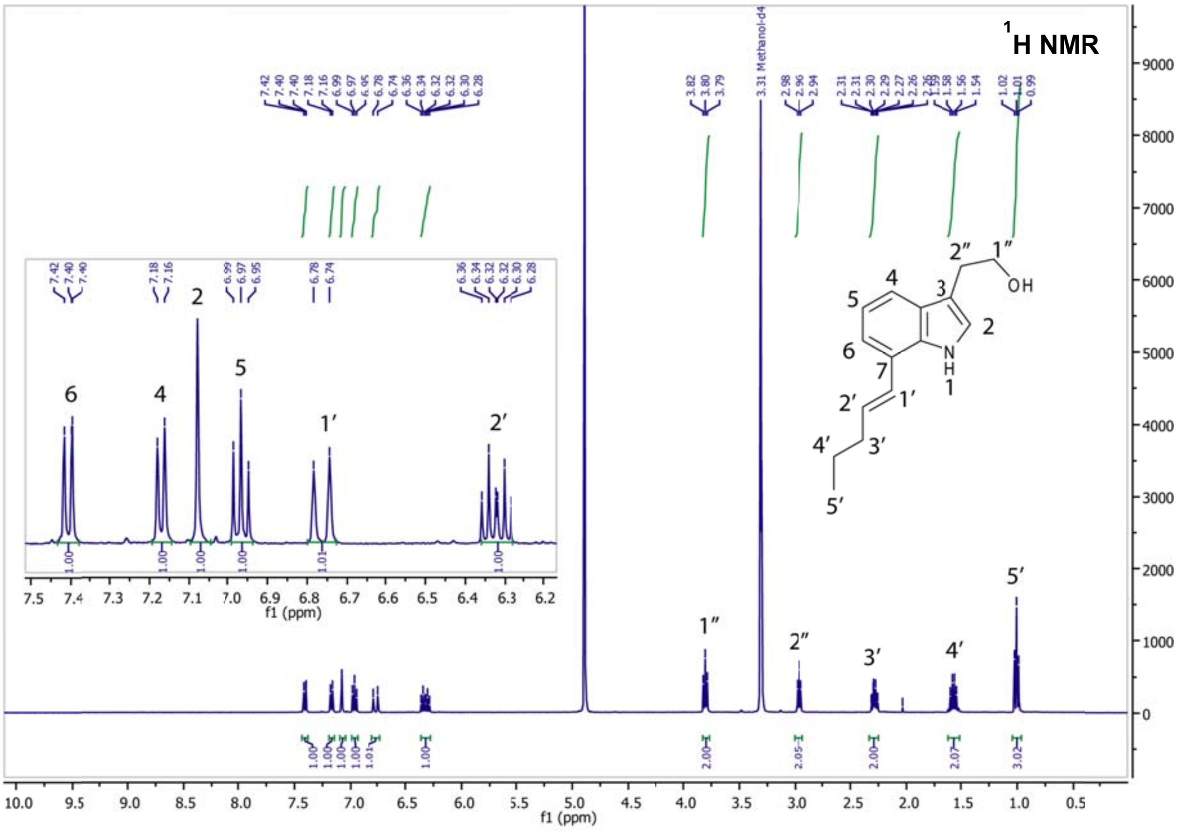

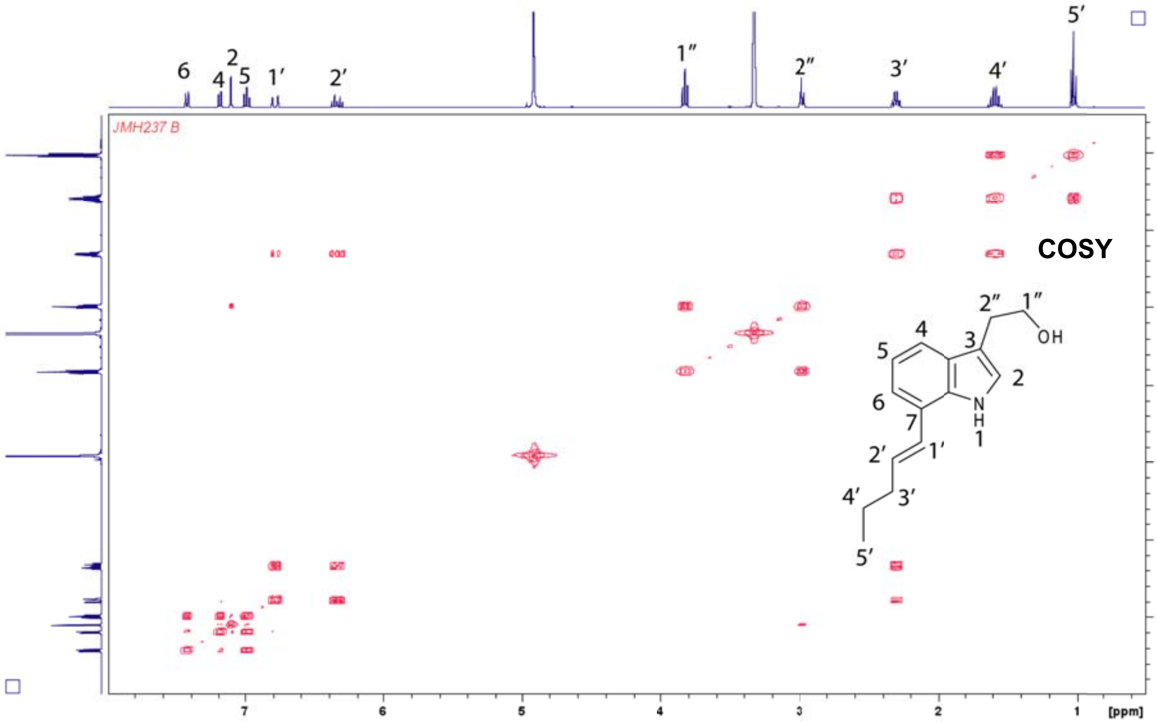

Supplementary Figure 34: ^{1}H NMR and COSY of 7-(pent-1-en-1-yl) tryptophol (**18**).

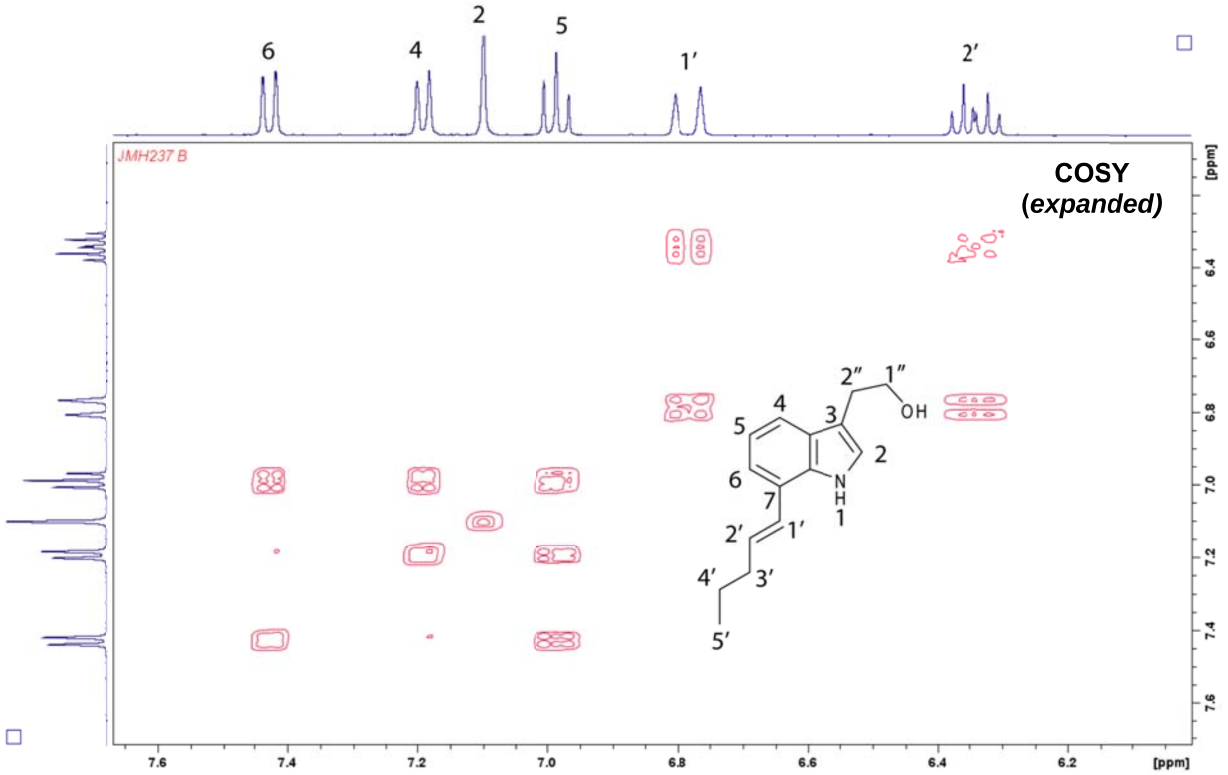

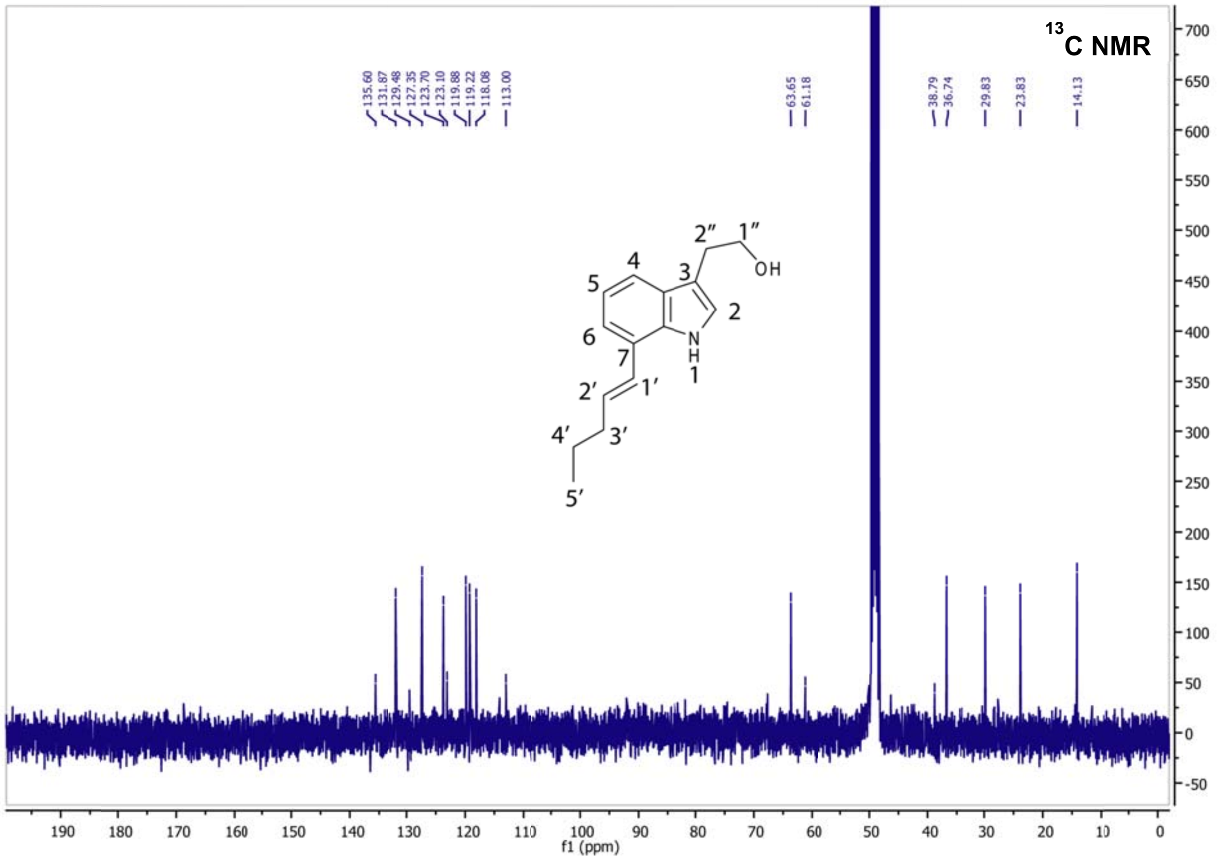

Supplementary Figure 35: Expanded COSY and ^{13}C NMR of 7-(pent-1-en-1-yl) tryptophol (**18**).

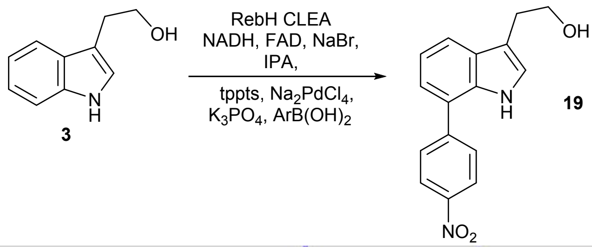

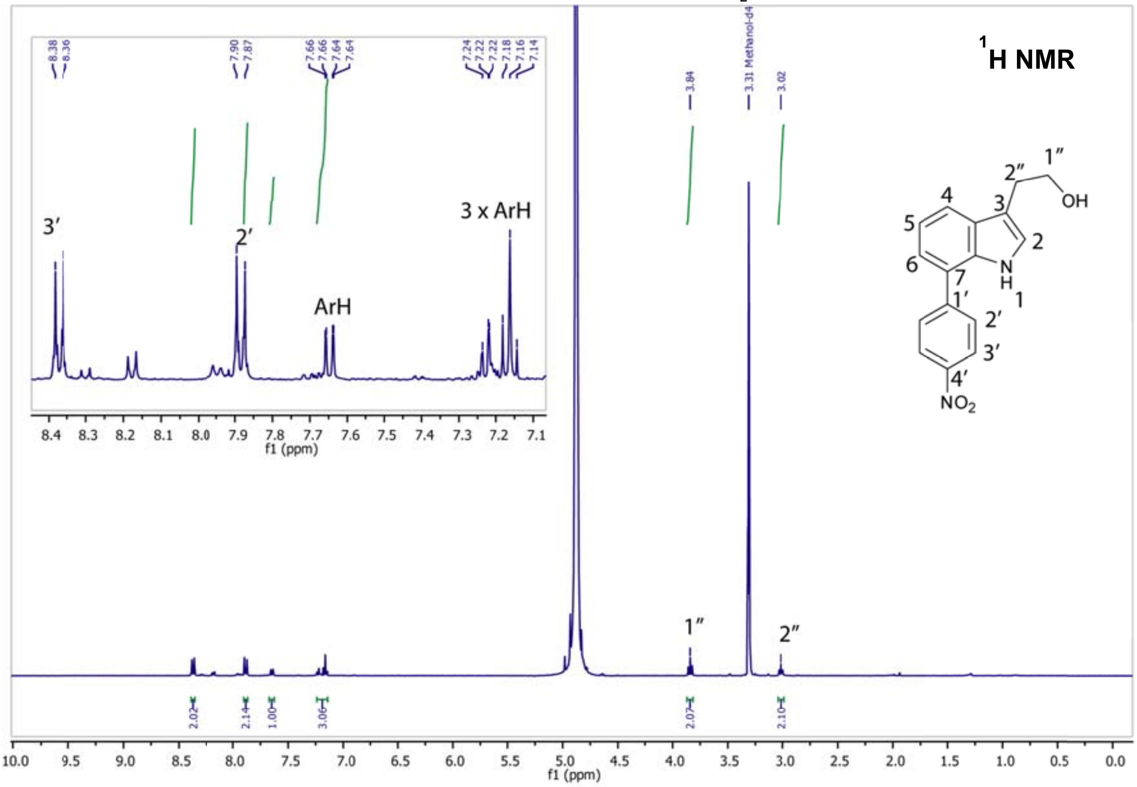

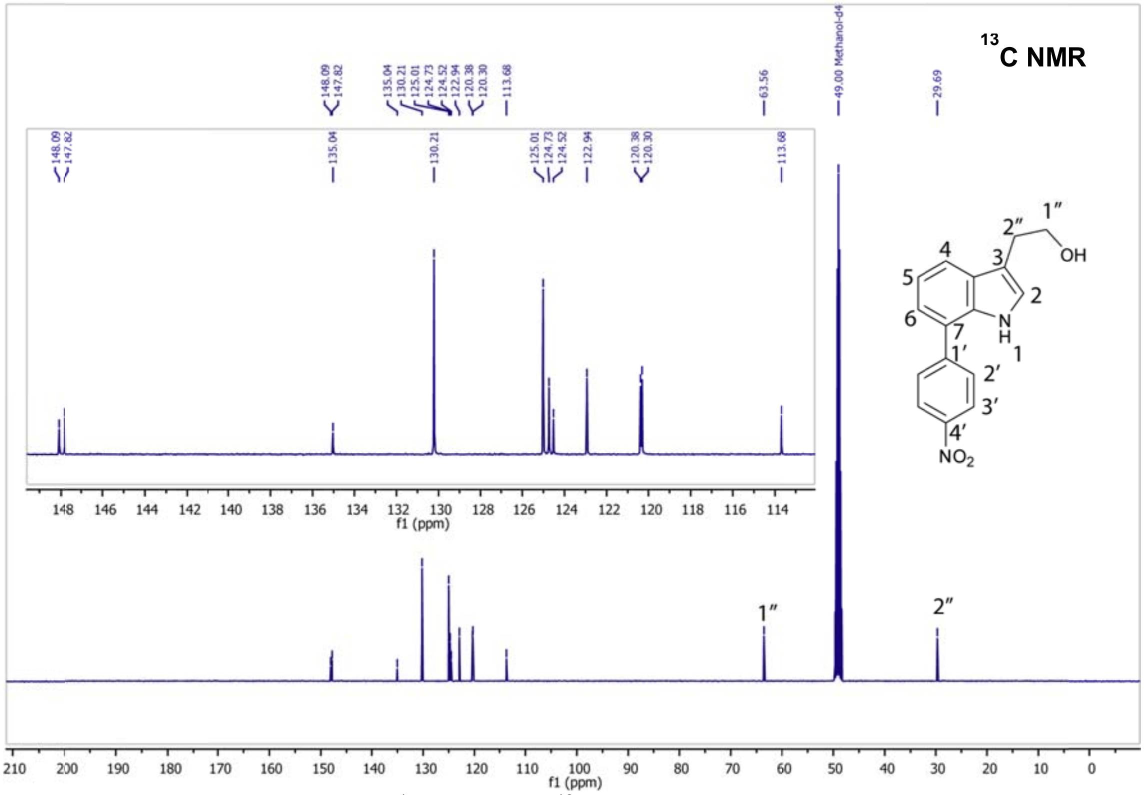

Supplementary Figure 36: ^{1}H NMR and ^{13}C NMR of 7-(4-nitrophenyl) tryptophol (**19**).

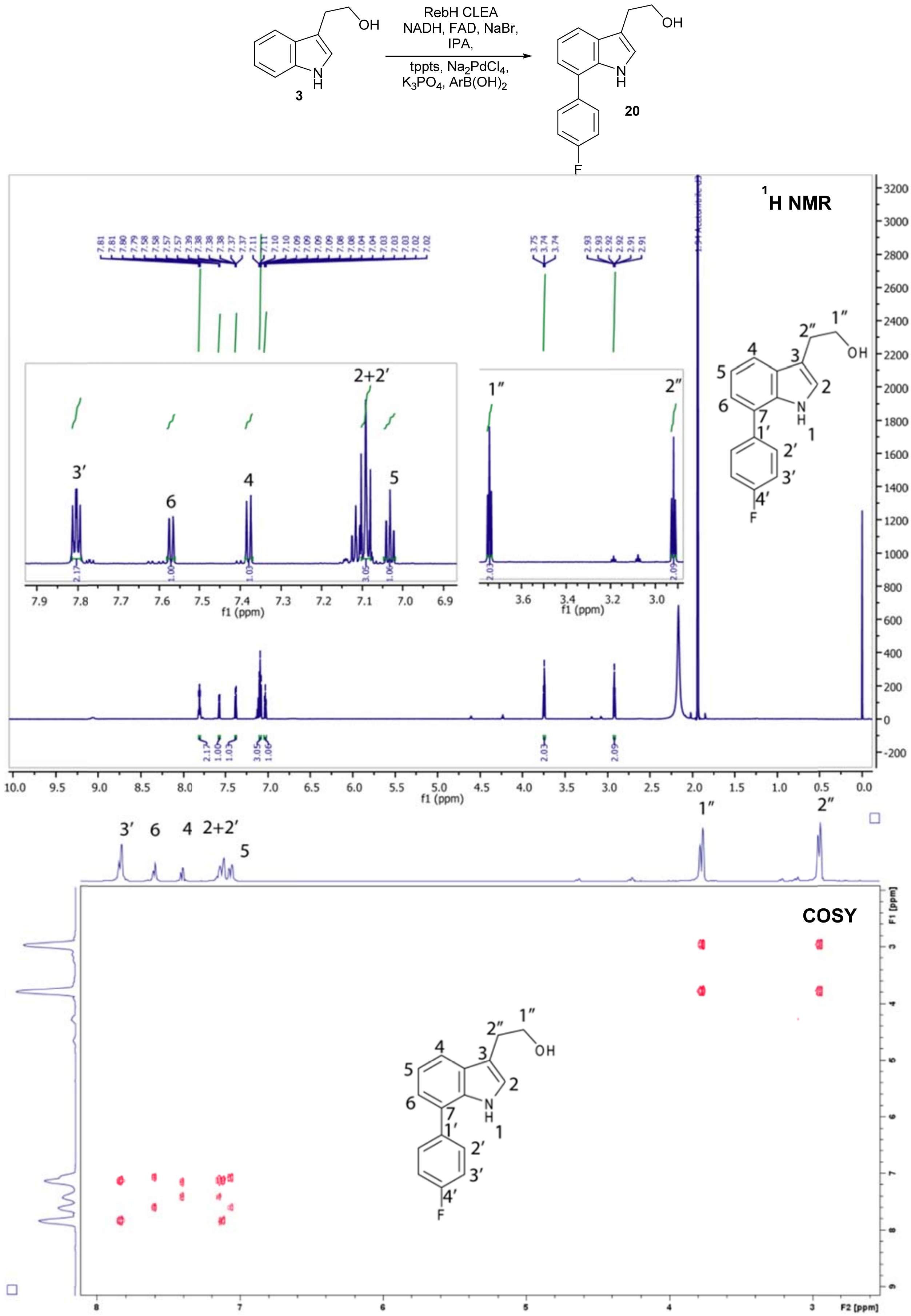

Supplementary Figure 37: ^{1}H NMR and COSY of 7-(4-fluorophenyl) tryptophol (**20**).

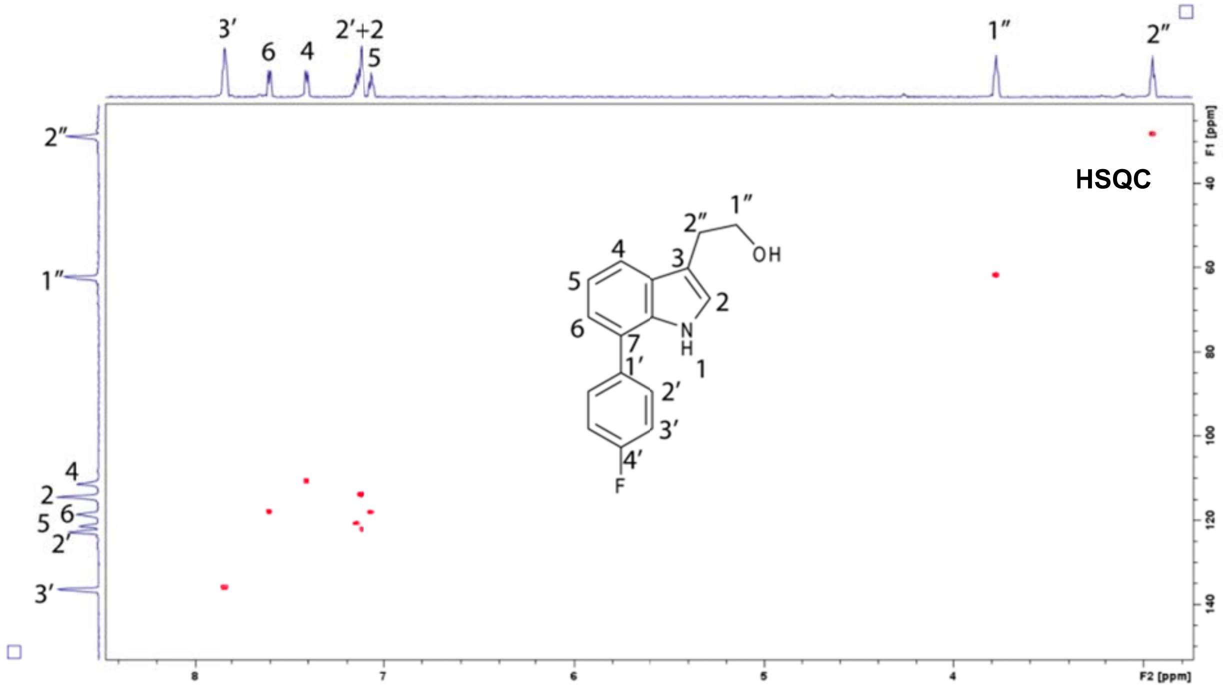

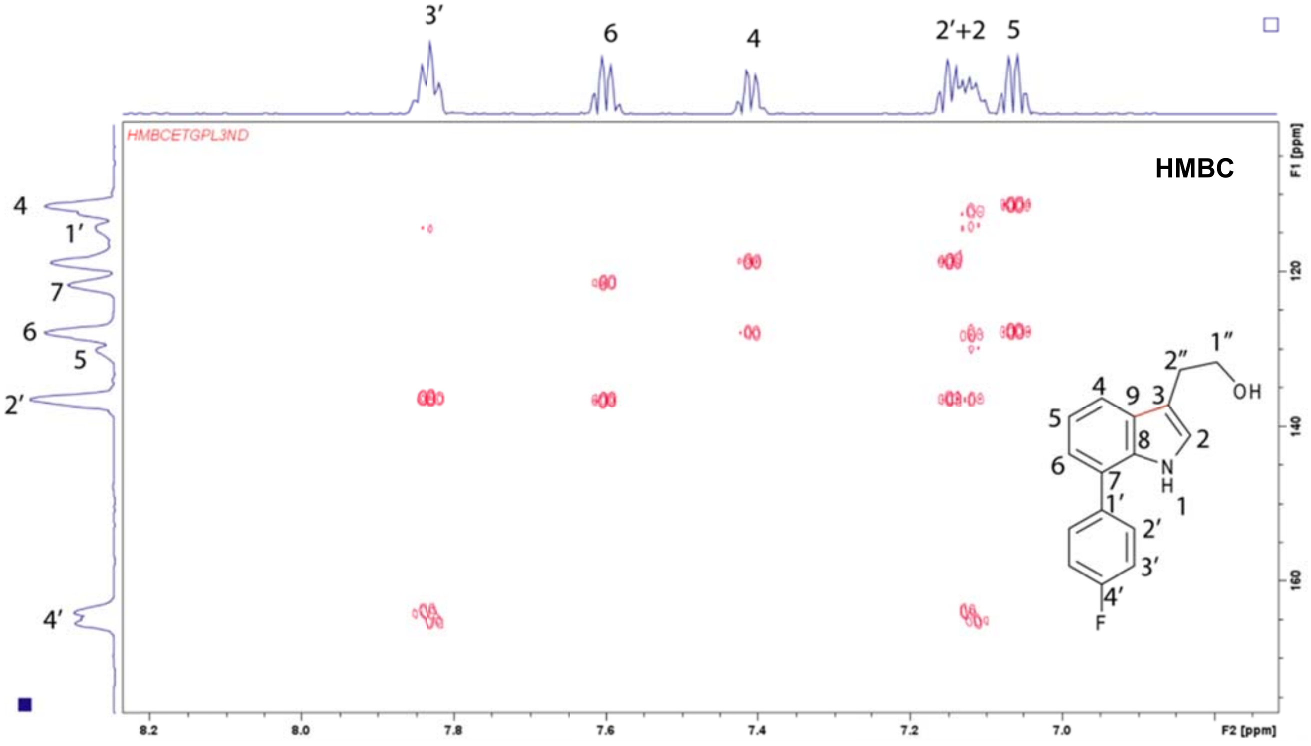

Supplementary Figure 38: HSQC and HMBC of 7-(4-fluorophenyl) tryptophol (**20**).

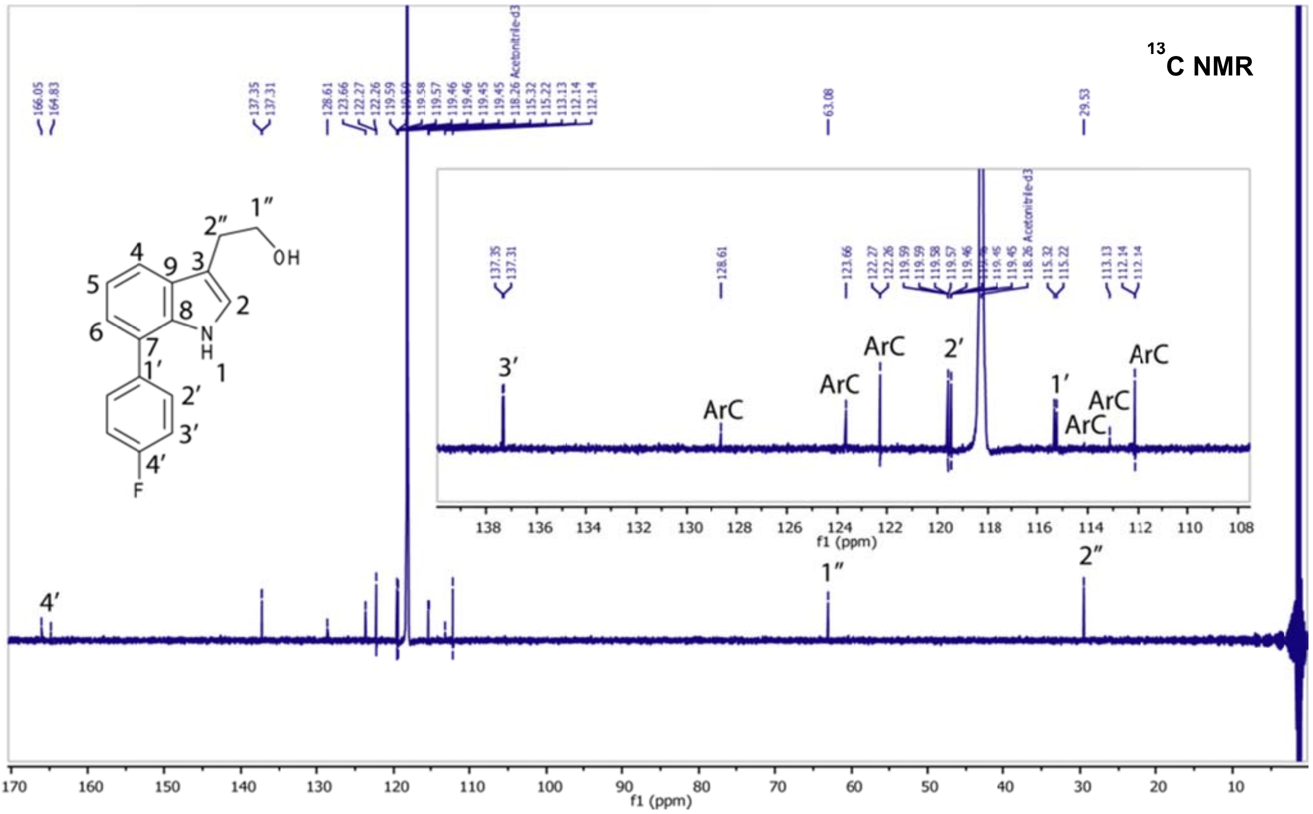

Supplementary Figure 39: ^{13}C NMR of 7-(4-fluorophenyl) tryptophol (**20**).

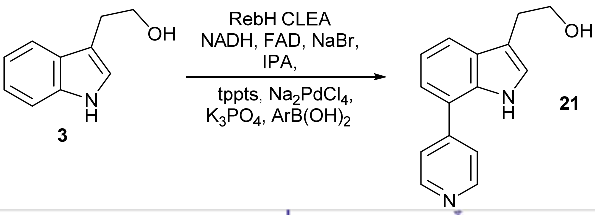

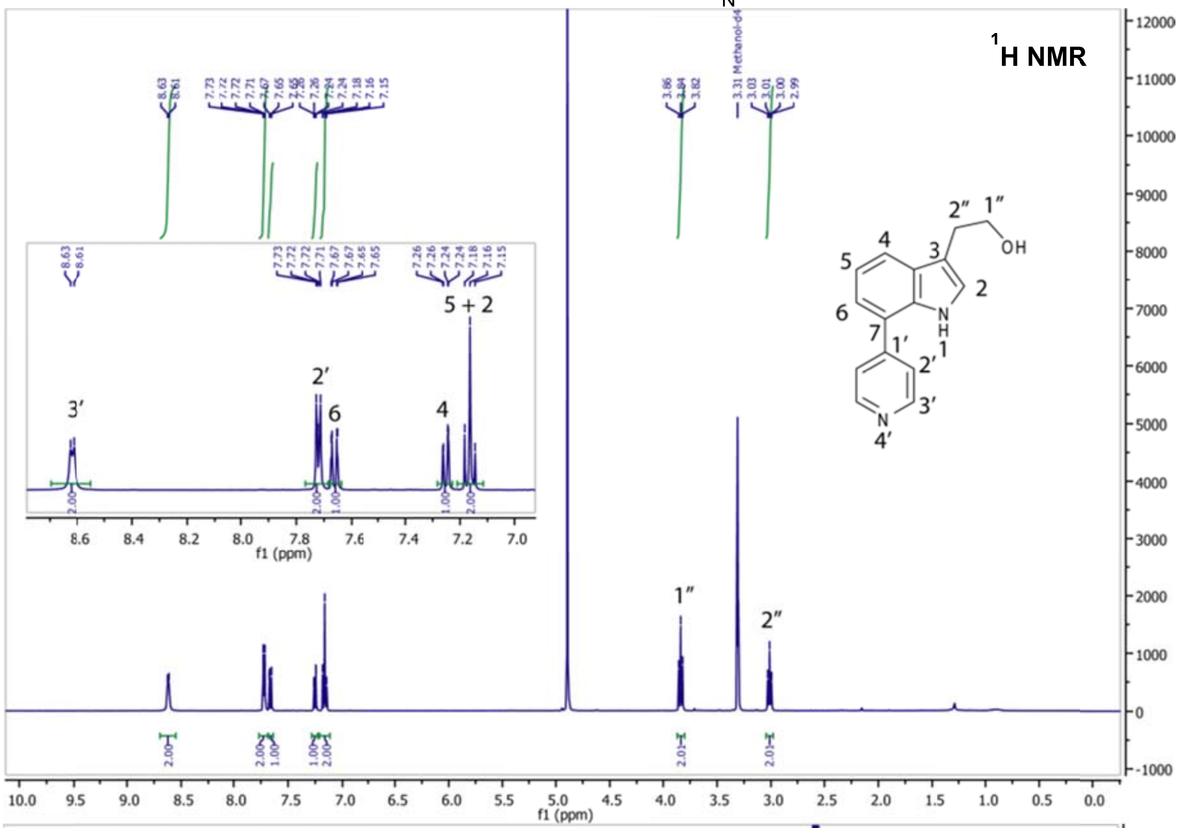

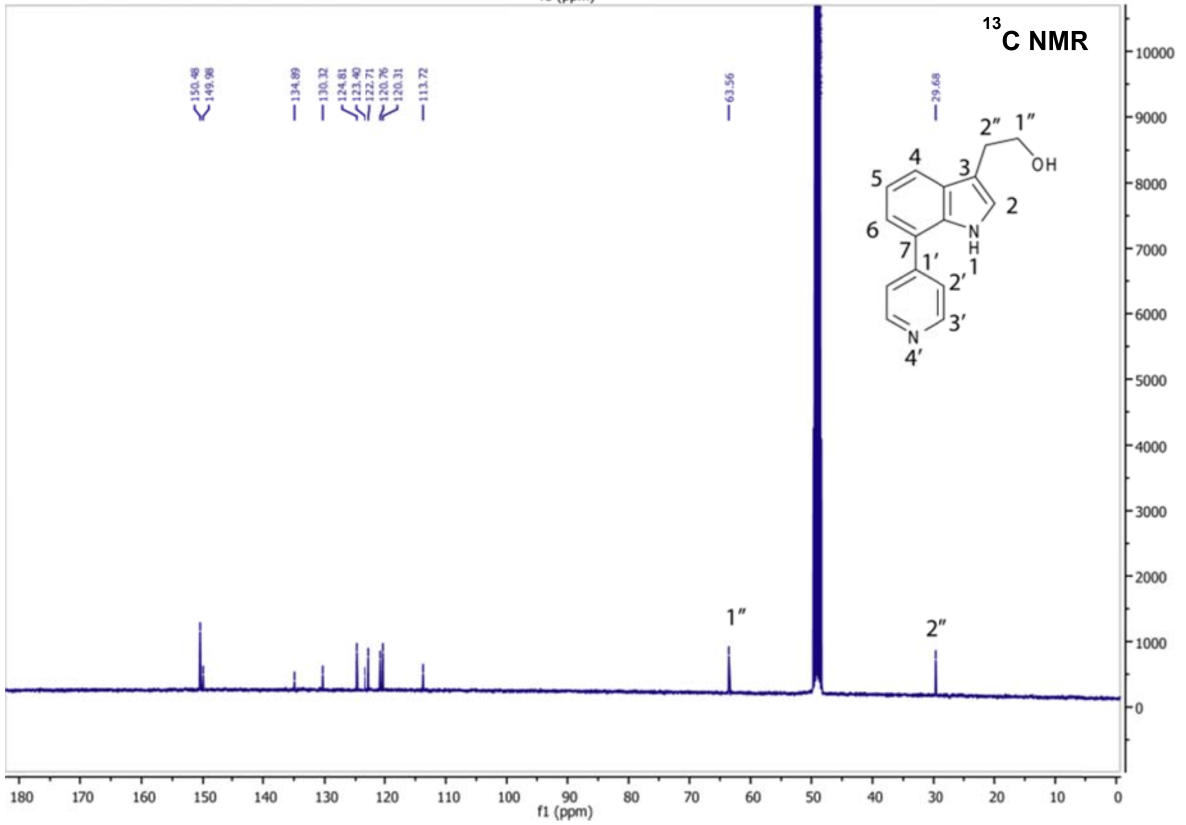

Supplementary Figure 40: ^{1}H NMR and ^{13}C NMR of 7-(pyrid-4-yl) tryptophol (**21**).

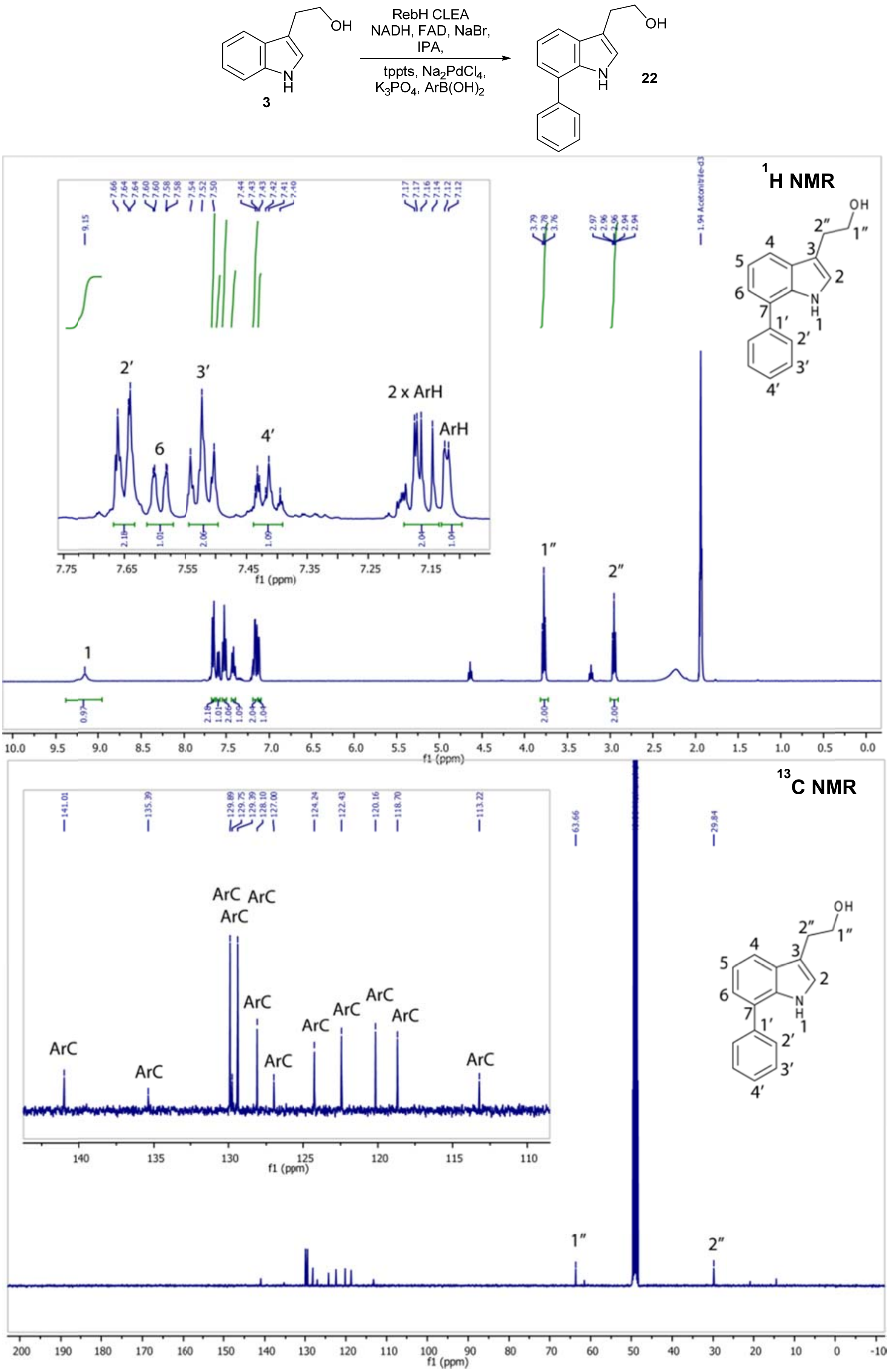

Supplementary Figure 41: ^{1}H NMR and ^{13}C NMR of 7-phenyl tryptophol (**22**).

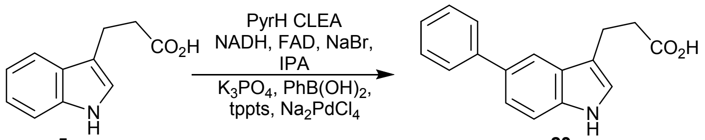

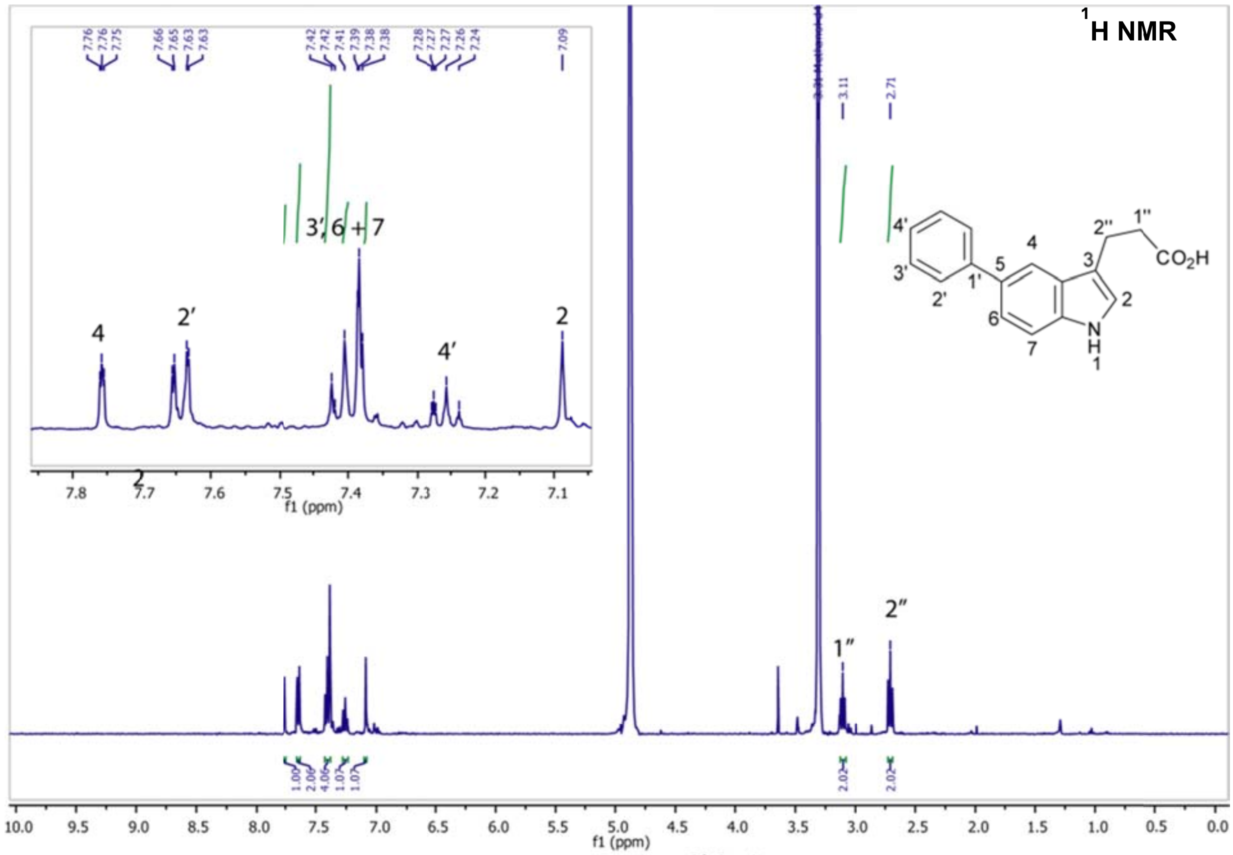

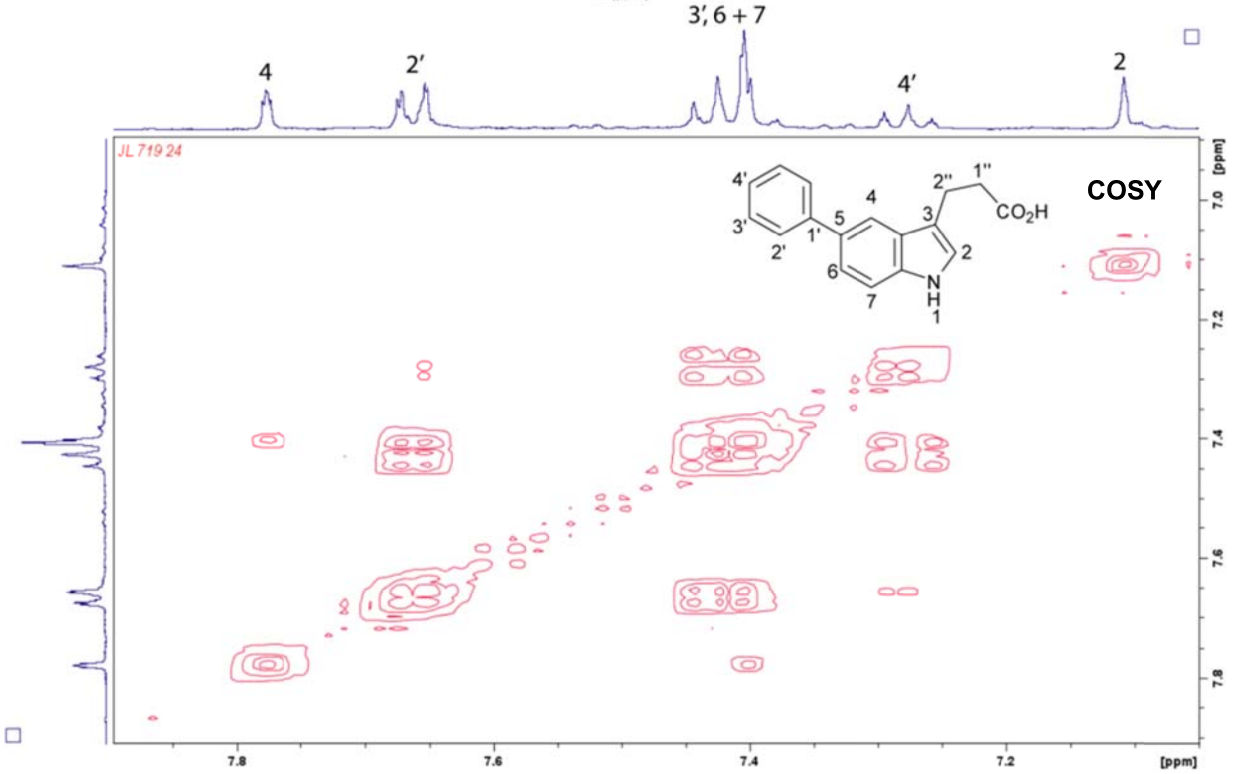

Supplementary Figure 42: ^{1}H NMR and COSY of 5-phenyl indole-3-propionic acid (**23**).

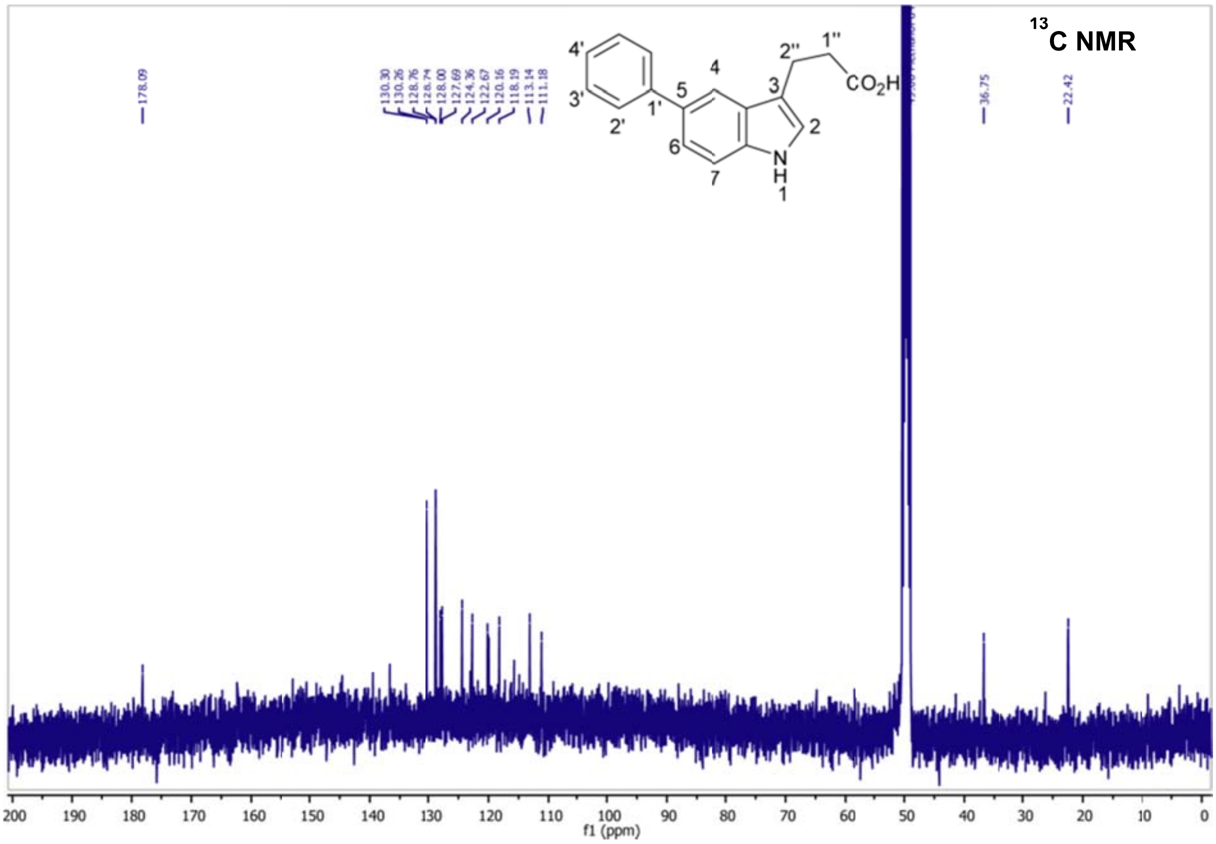

Supplementary Figure 43: ^{13}C NMR of 5-phenyl indole-3-propionic acid (**23**).

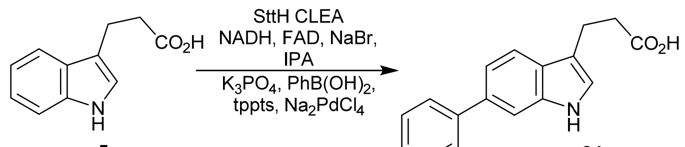

5 **24**

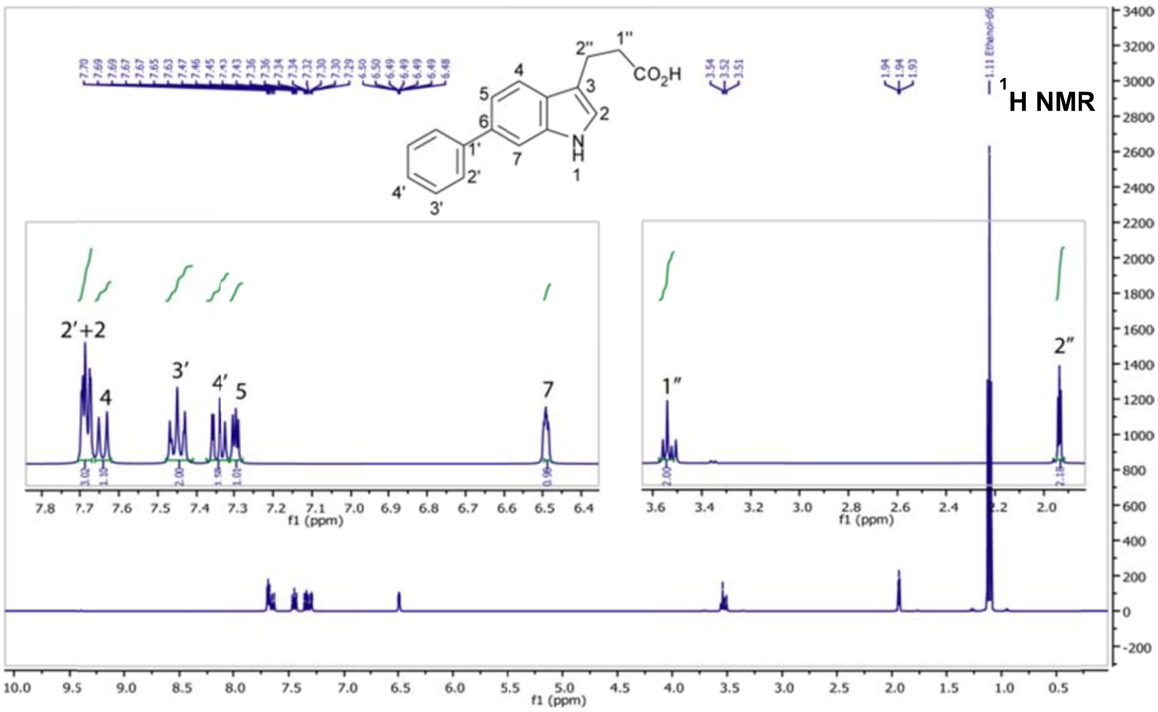

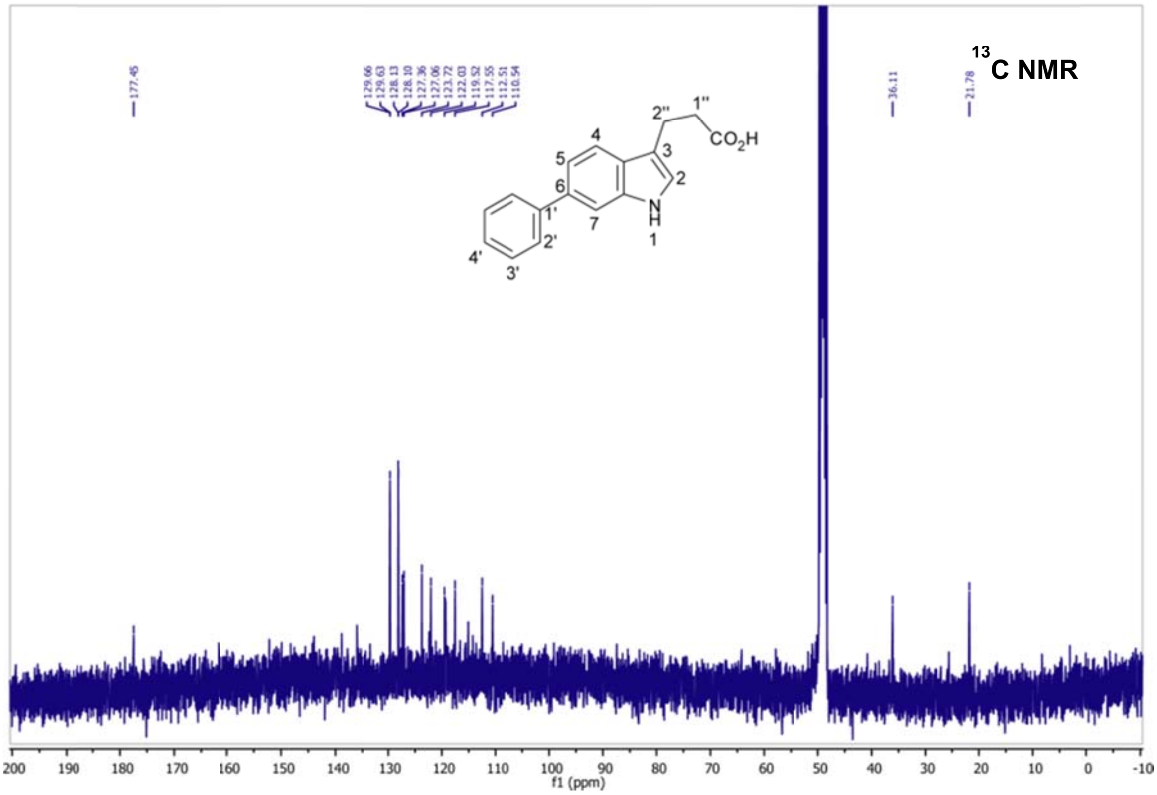

Supplementary Figure 44: ^{1}H NMR and ^{13}C NMR of 6-phenyl indole-3-propionic acid (**24**).

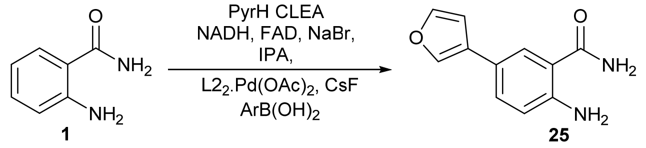

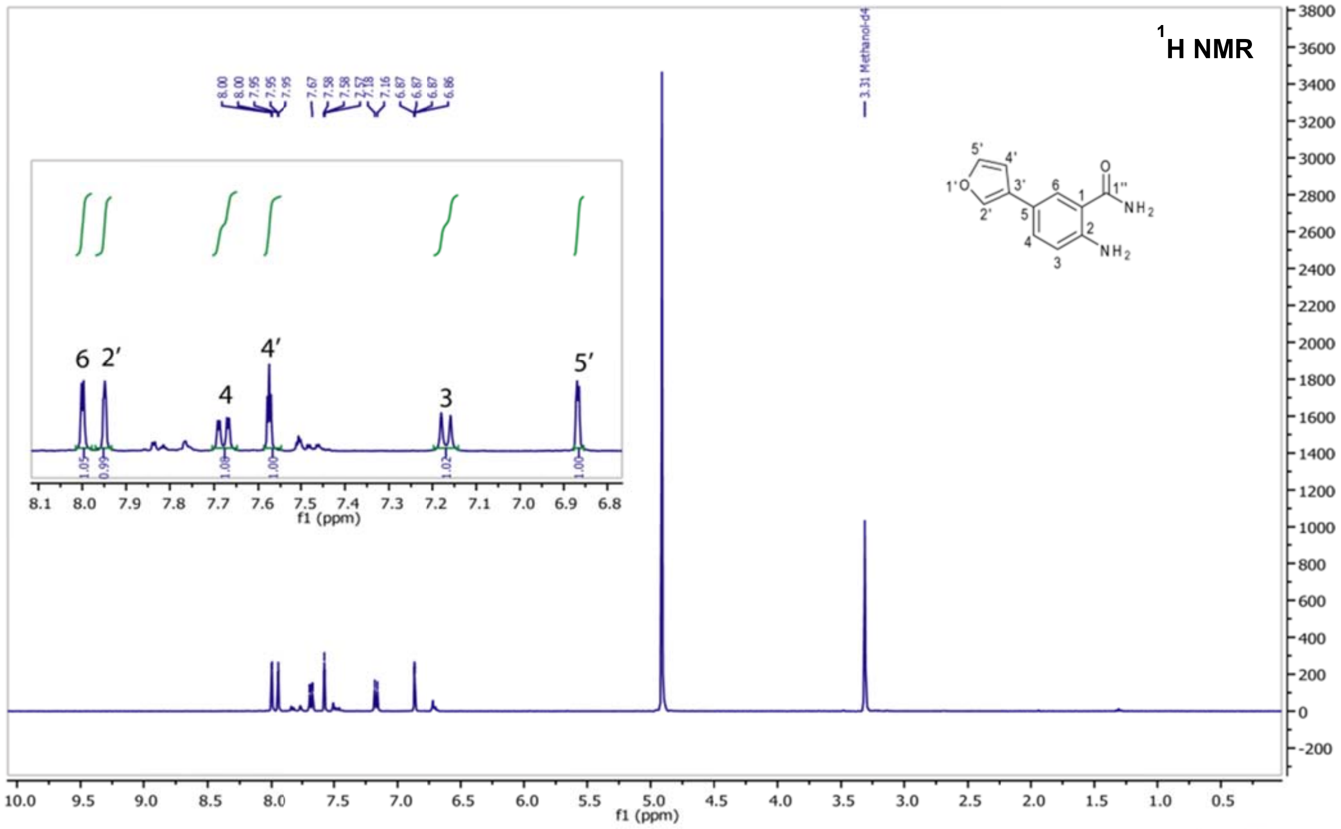

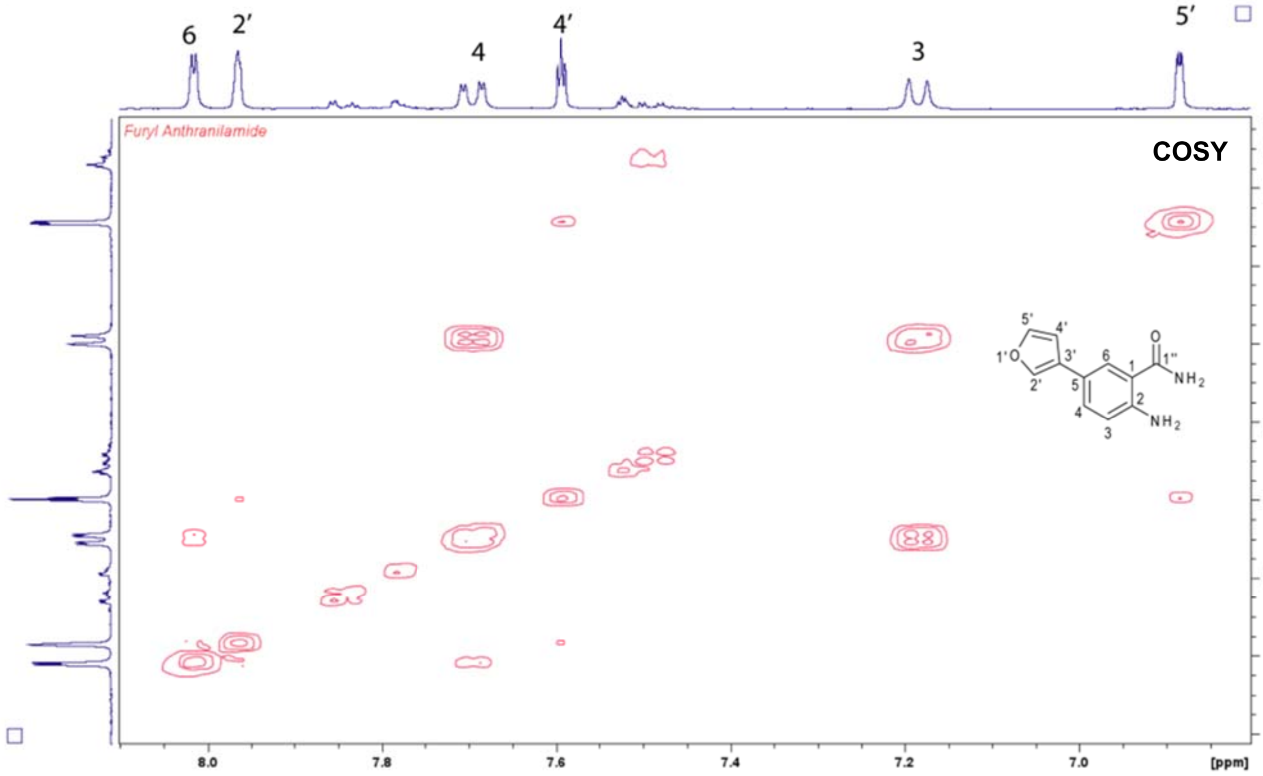

Supplementary Figure 45: ^{1}H NMR and COSY of 5-(furan-3-yl) anthranilamide (**25**).

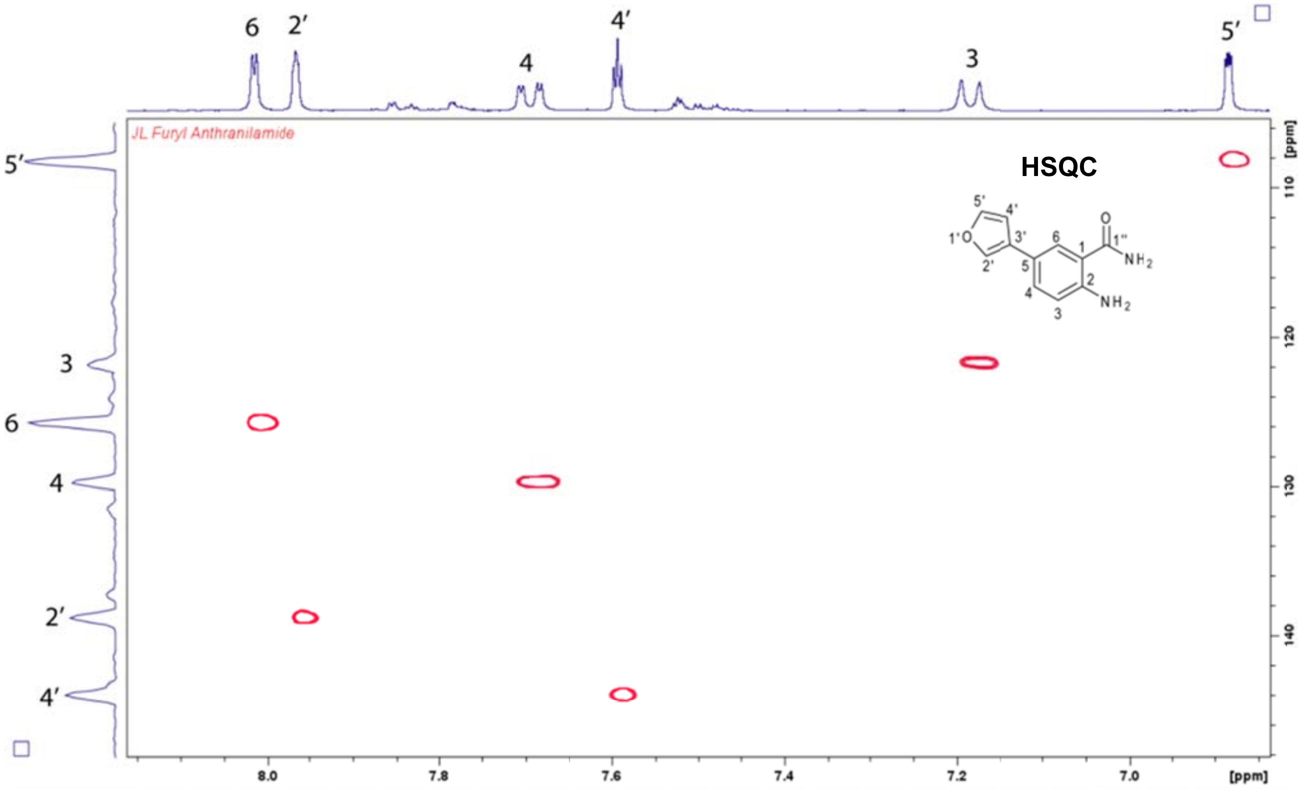

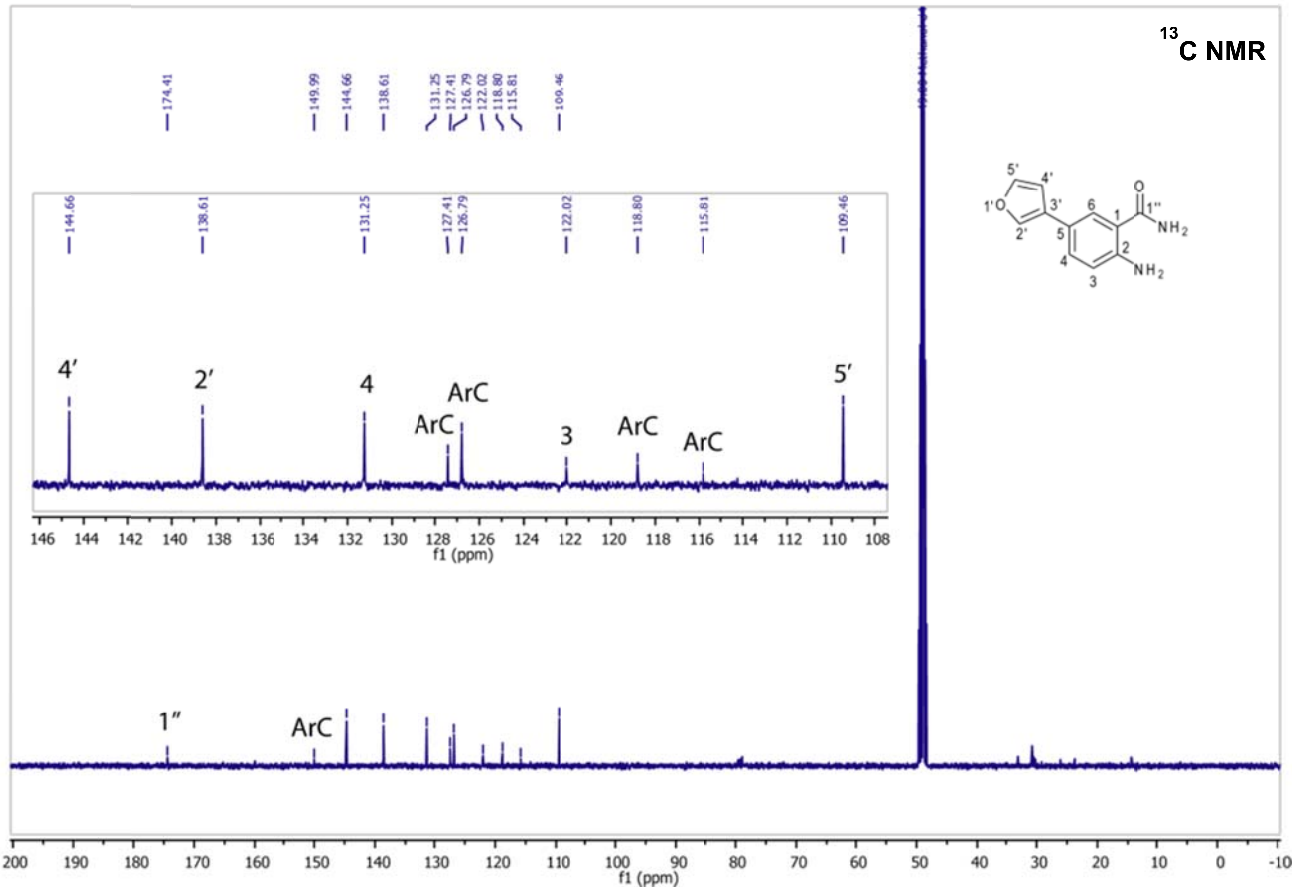

Supplementary Figure 46: HSQC and ^{13}C NMR of 5-(furan-3-yl) anthranilamide (**25**).

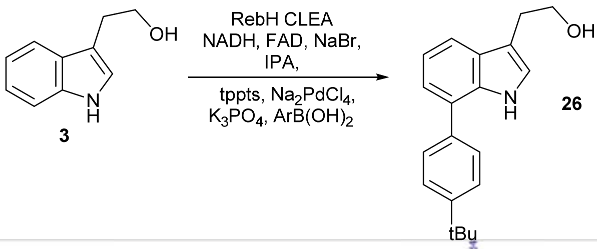

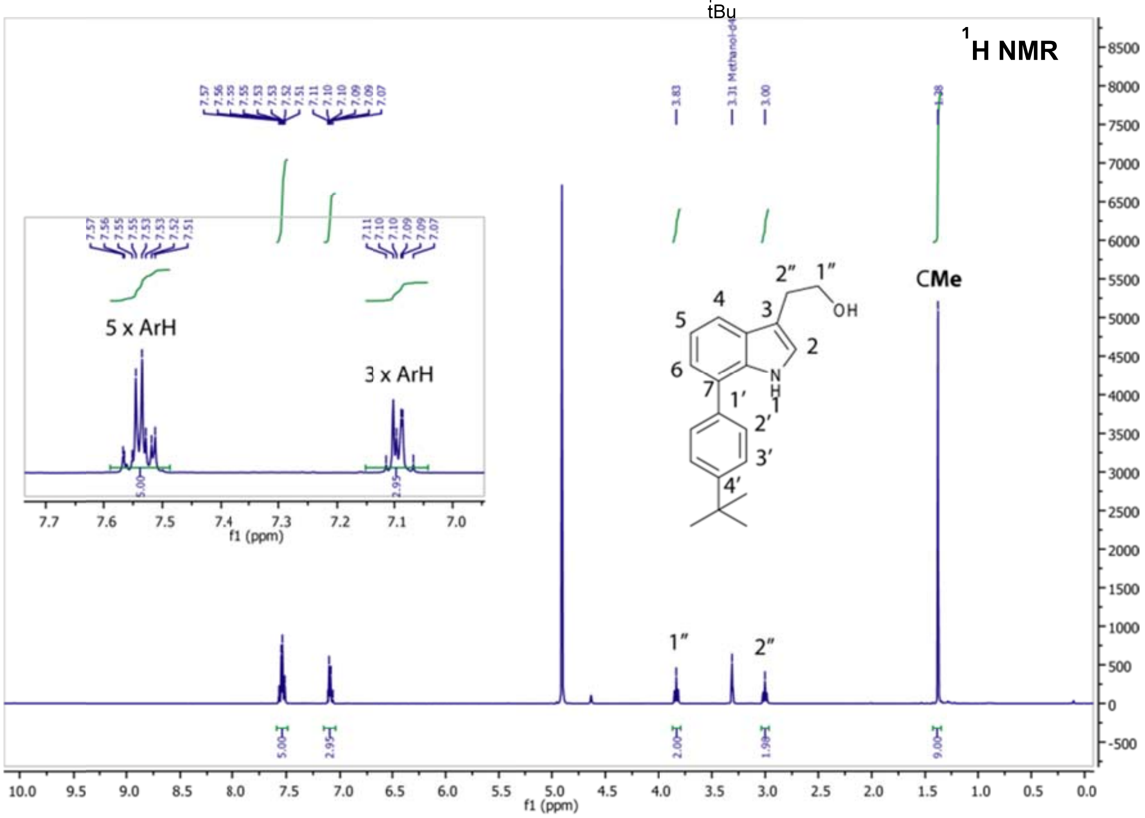

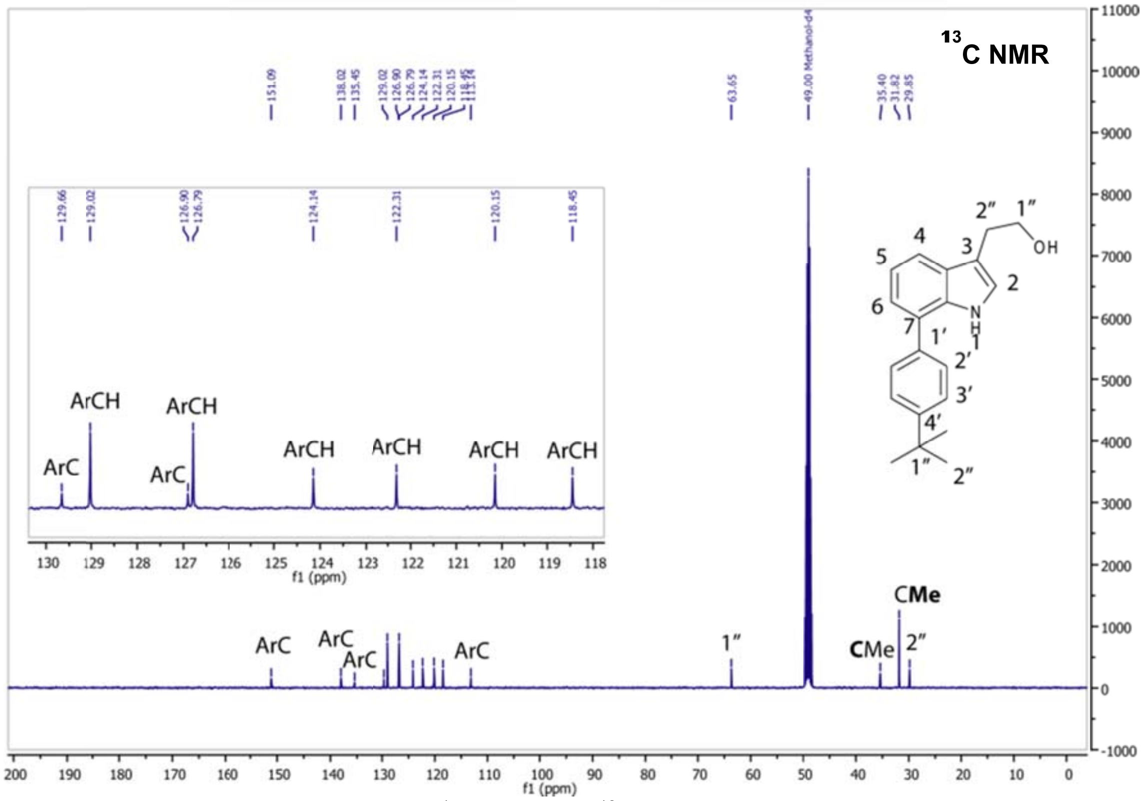

Supplementary Figure 47: ^{1}H NMR and ^{13}C NMR of 7-(4-terbutyl) tryptophol (**26**).

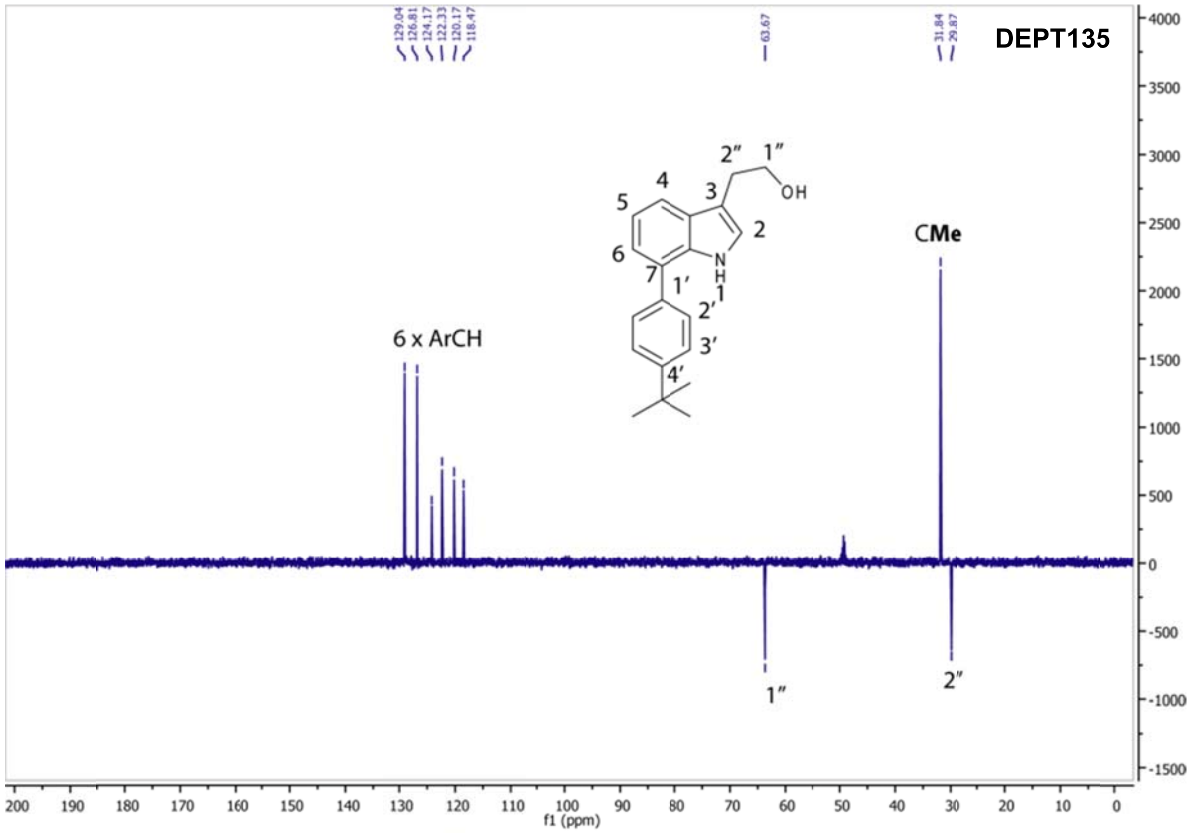

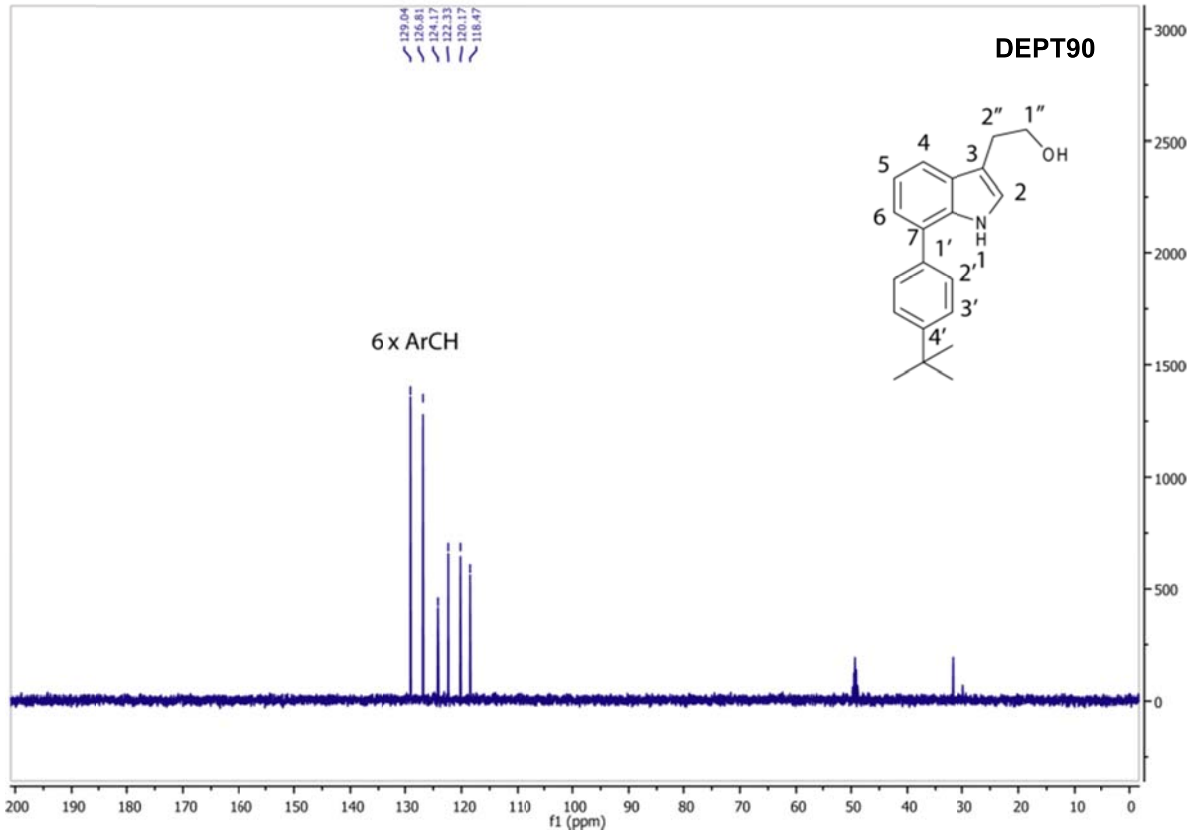

Supplementary Figure 48: DEPT135 and DEPT90 of 7-(4-terbutyl) tryptophol (**26**).

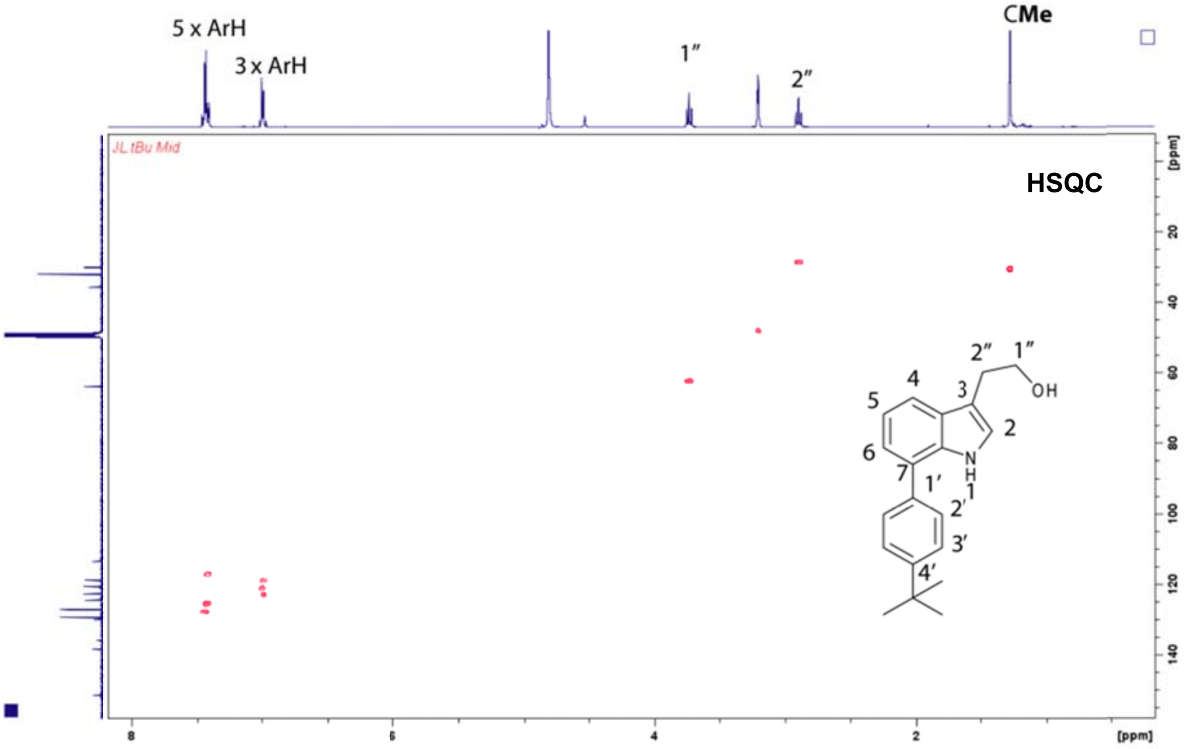

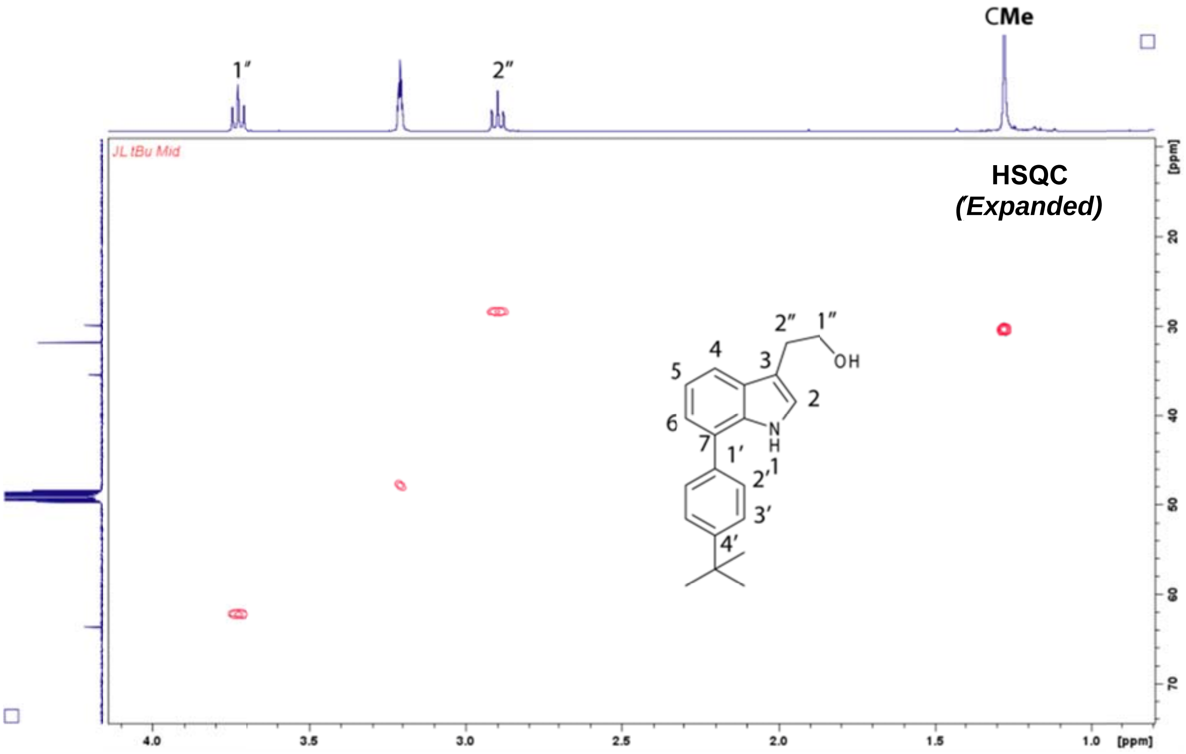

Supplementary Figure 49: HSQC and expanded HSQC of 7-(4-terbutyl) tryptophol (**26**).

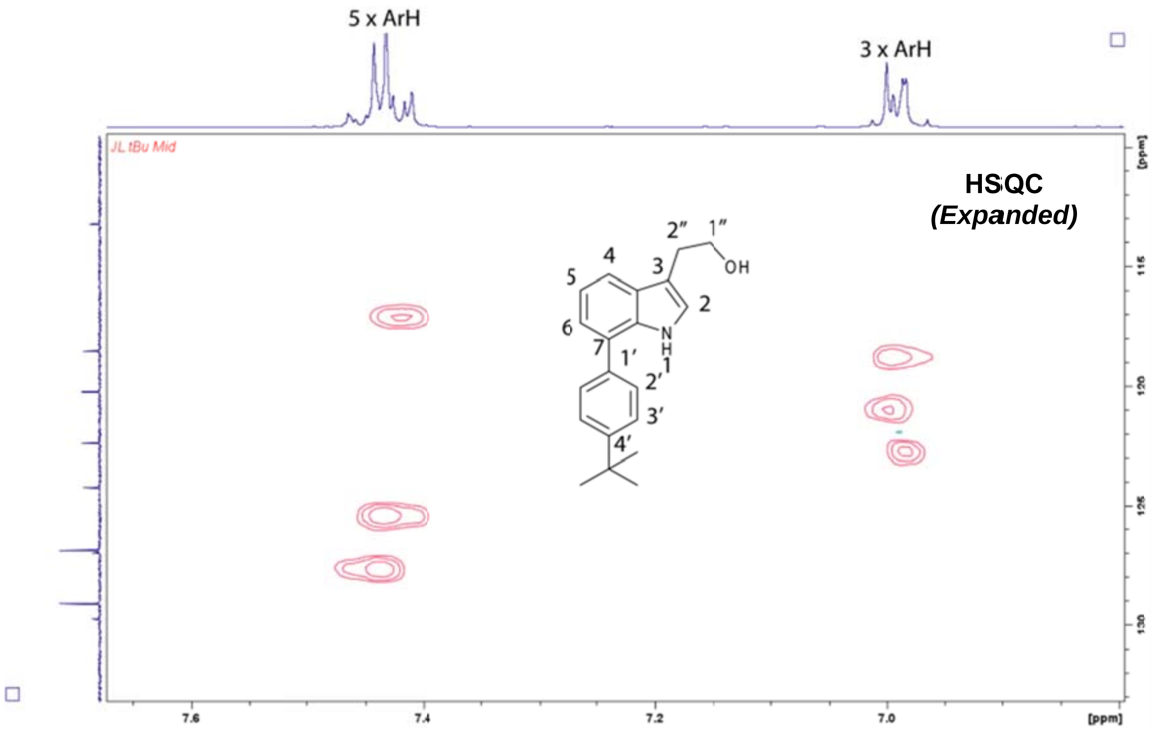

Supplementary Figure 50: Expanded HSQC of 7-(4-terbutyl) tryptophol (**26**).

Supplementary Methods

Protein Expression and Purification

The cloning of RebH and PyrH into pET 28a (+) and Fre into pET 45b (+) has been previously reported by our group.[1, 2] A construct containing GDH2 in pET 21b (+) was kindly provided by the Scrutton Group (University of Manchester). RebH and PyrH plasmids were transformed into chemically competent *E. coli* Arctic Express (DE3) for protein expression using kanamycin (50 µg/mL) and gentamycin (20 µg/mL) for selection whilst Fre and GDH plasmids were transformed into chemically competent *E. coli* BL21 (DE3) using ampicillin (50 µg/mL) for selection. Alcohol dehydrogenase from *Rhodococcus* sp. (ADH) was cloned into pET21b (+) and transformed into BL21(DE3) containing pGro7 (Takara) as previously described.[3]

A synthetic gene for the halogenase from *Streptomyces toxytricinis* (*SttH*) was codon optimised using GeneArt® and obtained from Invitrogen (UK). The *sttH* gene was sub-cloned into the pET28a (+) vector containing an N-terminal His-tag using the restriction enzymes *Nde*I and *NotI*. *E. coli* Arctic Express cells were subsequently transformed with the resulting *sttH*-containing vector for overexpression of the recombinant halogenase.

The *radH* gene from *Chaetomium chiversii* (NCBI Taxonomy ID: 155873) was synthesized (by GenScript, Piscataway, USA) and cloned into a pET 28b(+) protein expression vector (Invitrogen) using restriction sites *NdeI* and *XhoI*. The plasmid that contains the insert for *radH* gene was transformed into *E. coli* Rosetta 2 (DE3) cells (Novagen) for the overexpression of hexa histagged (both at N and C terminals) RadH halogenase. RadH from *C. chiversii* has not been isolated or expressed as a recombinant protein previously. This study is the first report of *in vitro* activity of RadH from this organism.

RebH, SttH and PyrH Expression. Single colonies of transformant were picked from the plates prepared above and used to inoculate 10 mL of LB medium containing kanamycin (50 µg/mL) and gentamycin (20 µg/mL). After incubation with shaking at 37 °C overnight, the resultant culture was diluted 1:100 into 100 mL of fresh LB containing kanamycin (50 µg/mL) and gentamycin (20 µg/mL). After a further incubation at 37 °C overnight, the culture was again diluted 1:100 into 10 x 440 mL of TB medium containing kanamycin (50 µg/mL) and grown at 37 °C and 180 rpm shaking until OD_{600} = 0.6 - 0.8 was reached. Cultures were then incubated at 4 °C for 30 minutes as cold shock prior to induction with IPTG (0.1 mM final) and incubated for a further 30 hrs. at 15 °C with shaking at 180 rpm. Cells were then harvested by centrifugation (4000 rpm, 4 °C, 20 min) and stored at – 20 °C prior to purification.

RadH Expression. Single colonies of transformant were picked from the plates prepared above and used to inoculate 10 mL of LB medium containing kanamycin (50 µg/mL) and chloramphenicol (30 µg/mL). After incubation with shaking at 37 °C overnight, the resultant culture was diluted 1:100 into 100 mL of fresh LB containing kanamycin (50 µg/mL) and chloramphenicol (30 µg/mL). After a further incubation at 37 °C overnight, the culture was again diluted 1:100 into 10 x 400 mL of 2YT medium containing kanamycin (50 µg/mL) and grown at 37 °C and 180 rpm shaking until OD_{600} = 0.4 - 0.6 was reached. Cultures were then induced with IPTG (0.1 mM final) and incubated for a further 30 hrs. at 18 °C and 180 rpm shaking. Cells were then harvested by centrifugation (4000 rpm, 4 °C, 20 min) and stored at – 20 °C prior to purification.

Fre and GDH Expression. Single colonies of transformant were picked from the plates prepared above and used to inoculate 10 mL of LB medium containing ampicillin (50 µg/mL). After incubation with shaking at 37 °C overnight, the resultant culture was diluted 1:100 into 100 mL of fresh LB containing ampicillin (50 µg/mL). After a further incubation at 37 °C overnight, the culture was again diluted 1:100 into 10 x 400 mL of LB medium containing ampicillin (50 µg/mL) and grown at 37 °C and 180 rpm shaking until OD_{600} = 0.4-0.6 was reached. Cultures were then induced with IPTG (1 mM final) and incubated overnight at 18 °C and 180 rpm shaking. Cells were then harvested by centrifugation (4000 rpm, 4 °C, 20 min) and stored at – 20 °C prior to purification.

ADH Expression. Alcohol dehydrogenase was expressed as previously reported by Frese *et. al*.[3]

Protein Purification Buffers. Buffers containing potassium phosphate buffer (pH 7.4 50 mM), NaCl (500 mM) and imidazole (10 mM, 80 mM or 300 mM) in distilled water were prepared and stored at 4 °C overnight before protein purification.

Protein Purification. Cell pellets were resuspended into 10 mM imidazole buffer containing 4 protease inhibitor tablets (*ca*. 60 mL total volume for cells from 4 L of culture broth). Cells were then lysed by sonication (10 min, 50 % pulse, 70 % power, 700 W) and the resultant lysate clarified by centrifugation (18, 000 rpm, 45 min, 4 °C). Ni-NTA (Qiagen) was equilibrated with 10 mM imidazole buffer prior to loading with clarified lysate under gravity flow. The resin was then washed with 80 mM imidazole buffer (3 column volumes) prior to elution with 300 mM imidazole buffer (5 column volumes). The eluted fraction was then buffer exchanged into 100 mM potassium phosphate buffer (pH = 7.2) using either dialysis or spin concentration. Protein concentration was then determined by using the A280 feature of a ThermoScientific 2000 Nanodrop. SDS-Page analysis showed that all proteins were obtained in high purity and reasonable yield.

ADH Purification.[3] ADH was purified using the method previously reported by Frese *et. al.*[3]

ADH and Fre Activity Determination. Activity units, U (µmol/min), were determined using the spectophotometric NAD^+/NADH reduction/oxidation method previously reported.[3]

CLEA Preparation. CLEAs were prepared based on previously reported methods.[4] The pET 28a (+) halogenase constructs were transformed into *E. coli* BL21 (DE3) containing pGro7 (Takara) using kanamycin (50 µg/mL) and chloramphenicol (20 µg/mL) for selection. Single colonies of transformant were then used to inoculate 10 mL of LB containing kanamycin (50 µg/mL) and chloramphenicol (20 µg/mL) and the resultant culture grown at 37 °C with shaking until OD_{600} = 0.6. This culture was then diluted 1:100 into 100 mL of fresh LB containing kanamycin (50 µg/mL) and chloramphenicol (20 µg/mL) and grown overnight at 37 °C with shaking. The overnight culture was then diluted 1:100 into 6 x 1 L of fresh LB containing kanamycin (50 µg/mL) and chloramphenicol (20 µg/mL). After a further incubation at 37 °C and shaking at 180 RPM until OD_{600} = 0.6, halogenase and chaperone expression was induced by addition of IPTG (0.1 mM overall) and L-arabinose (1 mg/mL). Incubation temperature was then dropped to 18 °C and cultures grown overnight. Cells were then collected by centrifugation (4000 RPM, 4 °C, 20 min) and the resultant cell pellets stored at – 20 °C before use. Cell pellets from total culture volume of 1.5 L were re-suspended into 30 mL of 100 mM sodium phosphate buffer (pH = 7.4) prior to lysis by sonication (12 min, 50 % pulse, 70 % power, 700 W). After clarification by centrifugation (10000 rpm, 4 °C, 30 min), purified Fre (2.5 U/mL) and ADH (1 U/mL) were added prior to mixing by inversion. Proteins were then precipitated by addition of ammonium sulphate (16.2 g, 95 % saturation) and agitated at 4 °C for 1 hr. Glutaraldehyde as a 50 % wt solution in water was then added (0.5 % w/v final concentration) prior to incubation with agitation at 4 °C for 2.5 hrs. The resultant aggregate was then collected by centrifugation (10000 rpm, 4 °C, 30 min) and washed with 40 mL of 100 mM sodium phosphate buffer (pH 7.4) three times. CLEA was then either re-suspended into reaction buffer or 100 mM sodium phosphate buffer (pH 7.4) for storage at 4 °C until use.

Analytical Scale Biotransformations

PyrH-Catalysed Bromination. To a solution containing anthranilamide (**1**) or 3-indolepropionic acid (**5**) (2.0 mM), NaBr (100 mM), glucose (20 mM), FAD (1.0 µM), PyrH (20 µM), Fre (2.0 µM) and GDH2 (12 µM) in 10 mM potassium phosphate buffer was added NADH (100 µM) to a total volume of 200 µL. After incubation at 20 °C with shaking at 300 rpm overnight, reactions were quenched by heating at 95 °C for 10 min. prior to centrifugation (13, 000 rpm, 10 min) and analysis of supernatant by analytical HPLC method 1.

SttH-Catalysed Bromination. To a solution containing 3-indolepropionic acid (**5**) (2.0 mM), NaBr (100 mM), glucose (20 mM), FAD (7.5 µM), SttH (10 µM), Fre (1.0 µM) and GDH2 (6.0 µM) in 10 mM potassium phosphate buffer was added NADH (100 µM) to a total volume of 200 µL. After incubation at 20 ^{o}C with shaking at 300 rpm overnight, reactions were quenched by heating at 95 ^{o}C for 10 min. prior to centrifugation (13, 000 rpm, 10 min) and analysis of supernatant by analytical HPLC method 2.

RebH-Catalysed Bromination. To a solution containing tryptophol (**3**) (2.0 mM), NaBr (100 mM), glucose (20 mM), FAD (100 µM), RebH (50 µM), Fre (5 µM) and GDH2 (10 µM) in 10 mM potassium phosphate buffer and 4% isopropanol was added NADH (100 µM) to a total volume of 200 µL. After incubation at 20 ^{o}C with shaking at 300 rpm overnight, reactions were quenched by addition of 200 µL of methanol. Protein was precipitated by heating at 95 ^{o}C for 10 min. prior to centrifugation (13, 000 rpm, 10 min) and analysis of supernatant by analytical HPLC method 2.

RadH-Catalysed Bromination. To a solution containing 6-hydroxyisoquinoline (**8**) (0.5 mM), NaBr (10 mM), FAD (1.0 µM), RadH (25 µM), Fre (4 µM) in 10 mM potassium phosphate buffer and 1% ethanol was added NADH (2.5 mM) to a total volume of 200 µL. After incubation at 30 ^{o}C with shaking at 300 rpm overnight, reactions were quenched by heating at 95 ^{o}C for 10 min. prior to centrifugation (13,000 rpm, 10 min) and analysis of supernatant by analytical HPLC method 3.

Preparative Scale Biotransformations

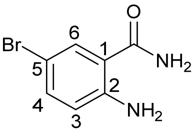

2

2-amino-5-bromo benzamide (2).[5] Biotransformation was prepared as per the general PyrH method detailed above to overall volume of 10 mL. After incubation at 20 ^{o}C in a loosely-capped flacon tube overnight, the reaction was boiled (95 ^{o}C, 10 min) prior to centrifugation (10, 000 rpm, 30 min, 4 ^{o}C) and lyophilisation. The resultant residue was then dissolved in methanol and purified by semi-preparative HPLC method 1 to afford the title compound as a white solid (3.9 mg, 91 %); ^{1}H NMR (400 MHz, MeOD) δ 7.53 (1H, d, *J* = 2.5 Hz, H6), 7.15 (1H, dd, *J* = 8.8, 2.5 Hz, H4), 6.72 (1H, d, *J* = 8.8 Hz, H3); *m/z* (ESI) 213 ([M^{79}Br-H]$^{-}$, 50 %), 215 ([M^{81}Br-H]$^{-}$, 50 %); HRMS *m/z* calcd for $C_7H_7ON_2{}^{79}BrNa$ 236.9634, found: 236.9635.

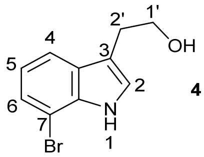

4

7-bromotryptophol (4).[6] Biotransformation was prepared as per the general RebH method detailed above to overall volume of 20 mL. After incubation at 20 ^{o}C in a loosely-capped flacon tube overnight, the reaction was boiled (95 ^{o}C, 10 min) prior to centrifugation (10, 000 rpm, 30 min, 4 ^{o}C) and lyophilisation. The resultant residue was then dissolved in methanol and purified by semi-preparative HPLC method 3 to afford the title compound as a white solid (4.4 mg, 92 %); ^{1}H NMR (400 MHz, $CDCl_3$) δ 7.57 (1H, d, *J* = 8.0 Hz, H6), 7.36 (1H, d, *J* = 8.0 Hz, H4), 7.17 (1H, d, *J* = 2.0 Hz, H2), 7.02 (1H, t, *J* = 8 Hz, H5), 3.91 (2H, t, *J* = 7.4 Hz, H1’), 3.02 (2H, t, *J* = 7.4 Hz, H2’); *m/z* (ESI) 239 ([M^{79}Br+H]$^{+}$, 50 %), 241 ([M^{81}Br+H]$^{+}$, 50 %); HRMS *m/z* cald for $C_{10}H_9{}^{79}BrNO$ ([M-H]$^{-}$) 237.9873, found: 237.9855.

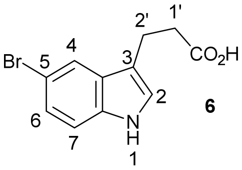

6

5-bromo-3-indolepropionic acid (6).[7] Biotransformation was prepared as per the general PyrH method detailed above to overall volume of 10 mL. After incubation at 20 ^{o}C in a loosely-capped flacon tube overnight, the reaction was boiled (95 ^{o}C, 10 min) prior to centrifugation (10000 rpm, 30 min, 4 ^{o}C) and lyophilisation. The resultant residue was then dissolved in methanol and purified by semi-preparative HPLC method 3 to afford the title compound as a white solid (4.6 mg, 87 %); ^{1}H NMR (400 MHz, MeOD) δ 7.68 (1H, d, *J* = 1.8 Hz, H4), 7.24 (1H, d, *J* = 8.6 Hz, H7), 7.16 (1H, dd, *J* = 8.6, 1.8 Hz, H6), 7.09 (1H, s, H2), 3.01 (2H, t, *J* = 7.6, 1'H), 2.61 (2H, t, *J* = 7.6 Hz, 2'H); *m/z* (ESI) 266 ([M^{79}Br-H]$^{-}$, 50 %), 268 ([M^{81}Br-H]$^{-}$, 50); HRMS *m/z* calcd for $C_{11}H_{11}{}^{79}BrNO_2$ 267.9968, found: 267.9964.

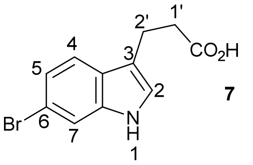

7

6-bromo-3-indolepropionic acid (7).[8] Biotransformation was prepared as per the general SttH method detailed above to overall volume of 10 mL. After incubation at 20 ^{o}C in a loosely-capped flacon tube overnight, the reaction was boiled (95 ^{o}C, 10 min) prior to centrifugation (10000 rpm, 30 min, 4 ^{o}C) and lyophilisation. The resultant residue was then dissolved in methanol and purified by semi-preparative HPLC method 4 to afford the title compound as a white solid (3.8 mg, 72 %); ^{1}H NMR (400 MHz, Acetone) δ 10.19 (1H, s, NH), 7.59 (1H, s, H7), 7.57 (1H, d, *J* = 8.4 Hz, H5), 7.22 (1H, s, H2), 7.17 (1H, d, *J* = 8.4 Hz, H4), 3.05 (2H, t, *J* = 7.6, H1'), 2.70 (2H, t, *J* = 7.6 Hz, 2'H); *m/z* (ESI) 266 ([M^{79}Br-H]$^{-}$, 50 %), 268 ([M^{81}Br-H]$^{-}$, 50); HRMS *m/z* calcd for $C_{11}H_{11}{}^{79}BrNO_2$ 267.9968, found: 267.9966. 5-bromo-3-indolepropionic acid (7) was also afforded as a minor by-product (0.6 mg, 12 %).

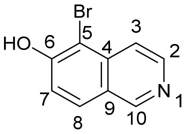

9

5-bromo-6-hydroxyisoquinoline (9).[9] Biotransformation was prepared as per the general RadH method detailed above to overall volume of 30 mL. After incubation at 30 ^{o}C in a loosely-capped flacon tube overnight, the reaction was boiled (95 ^{o}C, 10 min) prior to centrifugation (10000 rpm, 30 min, 4 ^{o}C) and lyophilisation. The resultant residue was then dissolved in methanol and water (1:1) before purification by semi-preparative HPLC method 2 to afford the title compound as a yellow solid (2.4 mg, 72 %); ^{1}H NMR (400 MHz, DMSO) δ 9.44 (1H, s, H10), 8.56 (1H, d, *J* = 6.4 Hz, H2), 8.25 (1H, d, *J* = 8.9 Hz, H7), 8.07 (1H, d, *J* = 6.4 Hz, H3), 7.57 (1H, d, *J* = 8.9 Hz, H8); *m/z* (ESI) 224 ([M^{79}Br+H]$^{+}$, 50 %), 226 ([M^{81}Br+H]$^{+}$, 50); HRMS *m/z* cald for $C_9H_7ON^{79}Br$ 223.9706, found: 223.9710.

General HPLC and LC-MS Methods

Analytical HPLC Method 1. 20 µL of supernatant injected onto Symmetry® C18 trapping column (5 µm, 180 µm x 20 mm) before separation using a Phenomenex Eclipse Plus® C18 analytical column (3.5 µ packing, 4.6 mm x 100 mm). Gradient starting conditions of 5% MeCN/H_2O (plus 0.05 % TFA) were held for 2 min. before development to 95 % MeCN/H_2O over 10 min. 95 % MeCN then held for 2 min. prior to re-equilibration to starting conditions over 3 min. Flow rates and column temperature were kept constant at 1 mL min^{-1} and 30 ^{o}C respectively. UV absorbance was detected at 254 nm, 280 nm and 310 nm throughout.

Analytical HPLC Method 2. 20 µL of supernatant injected onto Symmetry® C18 trapping column (5 µm, 180 µm x 20 mm) before separation using a Phenomenex Eclipse Plus® C18 analytical column (3.5 µ packing, 4.6 mm x 100 mm). Gradient starting conditions of 5% MeCN/H_2O (plus 0.05 % TFA) were held for 2 min. before development to 75 % MeCN/H_2O over 5 min. 75 % MeCN then held for 2 min. prior to washing at 95 % MeCN/H_2O for 2 min. and re-equilibration to starting conditions over 3 min. Flow rates and column temperature were kept constant at 1 mL min^{-1} and 30 ^{o}C respectively. UV absorbance was detected at 254 nm, 280 nm and 310 nm throughout.

Analytical HPLC Method 3. 20 µL of supernatant injected onto Symmetry® C18 trapping column (5 µm, 180 µm x 20 mm) before separation using a Phenomenex Eclipse Plus® C18 analytical column (3.5 µ packing, 4.6 mm x 100 mm). Gradient starting conditions of 5% MeCN/H_2O (plus 0.05 % TFA) were held for 2 min. before development to 95 % MeCN/H_2O over 7 min. 95 % MeCN/H_2O was then held for 2 min. and re-equilibration to starting conditions over 3 m. Flow rates and column temperature were kept constant at 1 mL min^{-1} and 30 ^{o}C respectively. UV absorbance was detected at 280 nm and 325 nm throughout.

Semi-Preparative HPLC Method 1. 1 mL of solution containing crude mixture dissolved in H_2O/MeCN was injected onto a Phenomenex Gemini® semi-preparative C18 HPLC column (5 µ packing, 250 x 10 mm). Starting conditions of 5 % MeCN/H_2O (plus 0.05 % TFA) were held for 3 min. prior to development to 95 % MeCN/H_2O over 23 min. 95 % MeCN/H_2O then held for 3 min. prior to re-equilibration of starting conditions over 3 min. Flow rates were kept constant at 5 mL min^{-1}. UV absorbance was detected at 280 nm and 310 nm throughout.

Semi-Preparative HPLC Method 2. 1 mL of solution containing crude mixture dissolved in MeCN/H_2O was injected onto a Phenomenex Gemini® semi-preparative C18 HPLC column (5 µ packing, 250 x 10 mm). Starting conditions of 5 % MeCN/H_2O (plus 0.05 % TFA) were held for 3 min. prior to development to 35 % MeCN/H_2O over 23 min. 95 % H_2O then held for 5 m. prior to re-equilibration of starting conditions over 3 min. Flow rates were kept constant at 5 mL min^{-1}. UV absorbance was detected at 280 nm and 325 nm throughout.

Semi-Preparative HPLC Method 3. 1 mL of solution containing crude mixture dissolved in MeCN/H_2O was injected onto a Phenomenex Gemini® semi-preparative C18 HPLC column (5 µ packing, 250 x 10 mm). Starting conditions of 5 % MeCN/H_2O (plus 0.05 % TFA) were held for 3 min. prior to development to 60 % MeCN/H_2O over 6 min. which was held for 5 min. followed by development to 5 % MeCN/H_2O over 3 min. 5 % MeCN/H_2O was then held for 3 min. prior to re-equilibration of starting conditions over 2 min. Flow rates were kept constant at 5 mL min^{-1}. UV absorbance was detected at 280 nm and 254 nm throughout.

Semi-Preparative HPLC Method 4. 1 mL of solution containing crude mixture dissolved in MeCN/H_2O was injected onto a Phenomenex Gemini® semi-preparative C18 HPLC column (5 µ packing, 250 x 10 mm). Starting conditions of 30 % MeCN/H_2O (plus 0.05 % TFA) were held for 20 min. prior to development to 95 % MeCN/H_2O over 5 min then re-equilibration of starting conditions over 3 min. Flow rates were kept constant at 5 mL min^{-1}. UV absorbance was detected at 254 nm and 280 nm throughout.

General LC-MS Method. 3 µL of supernatant was separated using the appropriate analytical HPLC method described above. Detection was performed using a Waters LCQ Orbitrap XL.

Synthesis of Standards and Ligands

Unless otherwise stated, all chemicals were purchased from Sigma-Aldrich, Fischer or Arcos organics and used without further purification. NMR analysis was performed on a Bruker 400 MHz or 800 MHz spectrometer. Mass spectrometry was performed on a Waters LCT Time-of-Flight Mass Spectrometer. IR spectroscopy was performed on a Bruker Alpha-P IR spectrometer. Melting points are unadjusted and were measured on a Gallenklamp MPD350 melting point apparatus.

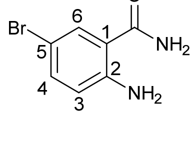

2

2-amino-5-bromobenzamide (2).[5] An oven dried flask containing 2-amino-5-bromobenzoic acid (1.0 mmol, 1.0 eq), 1,1'-carbonyl diimidazole (1.1 mmol, 1.0 eq) and stirrer bar was purged with nitrogen prior to addition of anhydrous THF (10 mL). The colourless solution was then stirred at room temperature for

6 hrs. before addition of saturated ammonia solution (10 mL) and further stirring overnight. THF was then removed under a stream of nitrogen and product extracted into EtOAc (3 x 10 mL). After removal of solvent *in vacuo* the crude was purified by flash chromatography (SiO_2; Et_2O) to afford pure product as a white solid (157 mg, 74 %); mp 182 – 185 °C; v_{max}/cm^{-1} 3394 (NH_2 str.), 3349 (NH_2 str.), 3287 ($CONH_2$ str.), 3156 ($CONH_2$ str.), 2956, 2921, 2852 (ArCH str.), 1738 ($CONH_2$), 1675, 1604, 1579 (C=C str.); ^{1}H NMR (400 MHz, DMSO) δ 7.84 (1H, br. s, $CONH_2$), 7.69 (1H, d, *J* = 2.4 Hz, H6), 7.25 (1H, dd, *J* = 8.8, 2.4 Hz, H4), 7.19 (1H, br. s, $CONH_2$), 6.72 (2H, br. s, NH_2), 6.65 (1H, d, *J* = 8.8 Hz, H3); ^{1}H NMR (400 MHz, MeOH) δ 7.53 (1H, d, *J* = 2.5 Hz, H6), 7.15 (1H, dd, *J* = 8.8, 2.5 Hz, H4), 6.72 (1H, d, *J* = 8.8 Hz, H3); ^{13}C NMR (101 MHz, DMSO) δ 169.96 (C=O), 149.40 (C2), 134.33 (C4), 130.77 (C6), 118.50 (C1), 115.15 (C3), 104.71 (C5); *m/z* (ESI) 213 ([M^{79}Br-H]$^-$, 50 %), 215 ([M^{81}Br-H]$^-$, 50); HRMS *m/z* cald for $C_7H_7ON_2Br_1Na_1$ 236.9634, found: 236.9635; λ_{max} (H_2O/CH_3CN) 228, 248 and 328 nm.

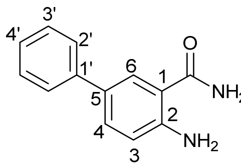

10

2-amino-5-phenylbenzamide (10).[10] A flask fitted with a stirrer bar was charged with 2-amino-5-bromobenzamide (**2**) (83.6 mg, 0.39 mmol, 1.0 eq), phenyl boronic acid (125 mg, 1.02 mmol, 2.6 eq) and potassium carbonate (500 mg, 3.6 mmol, 9.0 eq) was backfilled with nitrogen. Tetrakis(triphenylphosphine)palladium^0 (49 mg, 42 µmol, 10 mol %) was then added under positive pressure of nitrogen prior to addition of degassed toluene (7.5 mL), water (5 mL) and ethanol (2.5 mL) in succession. The orange solution was then heated at reflux with stirring overnight. After cooling, DCM (25 mL) and saturated ammonium chloride (25 mL) were added and the layers separated. The aqueous phase was extracted with further DCM (2 x 25 mL) and the combined organics washed with saturated sodium hydrogen carbonate (25 mL) and water (25 mL) prior to concentration *in vacuo* and purification by semi-preparative HPLC method 1. The title compound was afforded as an off-white solid (72.8 mg, 88 %); mp 159 – 161 °C; v_{max}/cm^{-1} 3399 (NH_2 str.), 3299 (NH_2 str.),3173 ($CONH_2$ str.), 3051, 3022 (ArCH str), 1676 (C=O str.), 1617, 1509 (C=C str.); ^{1}H NMR (400 MHz, DMSO) δ 7.96 (1H, br. s, $CONH_2$), 7.86 (1H, d, *J* = 2.1 Hz, H6), 7.65 (2H, dd, *J* = 8.3, 1.1 Hz, H2'), 7.50 (1H, dd, *J* = 8.5 Hz, H4), 7.40 (2H, t, *J* = 7.7 Hz, H3'), 7.25 (1H, t, *J* = 7.3 Hz, H4'), 7.14 (1H, br. s, $CONH_2$), 6.78 (1H, d, *J* = 8.6 Hz, H3), 6.70 (2H, br. s, NH_2); ^{13}C NMR (101 MHz, DMSO) δ 171.22 (C=O), 150.1 (C-N), 131.2 (ArC), 128.6 (ArCH), 126.9 (ArCH), 126.3 (ArCH), 126.0 (ArCH), 125.6 (ArCH), 117.1 (ArCH), 114.2 (ArC-C); *m/z* (ESI) 213 ([M+H]$^+$, 20 %), 196 ([M-NH_3]$^+$, 100), 168 ([M-$CONH_2$]$^+$); HRMS m/z cald for $C_{13}H_{10}NO$ [M-NH_2]$^+$ 196.0757, found: 196.0754; λ_{max} (H_2O/CH_3CN) 278 and 336 nm.

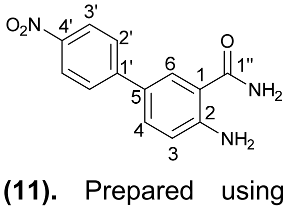

11

2-amino-5-(2-nitrophenyl)benzamide (11). Prepared using the above method from 2-amino-5-bromobenzamide (**2**) and 4-nitrophenyl boronic acid. Purification by flash chromatography (SiO_2; Et_2O) afforded the title compound as an orange solid (92 mg, 92 %); ^{1}H NMR (400 MHz, CD_3CN) δ 7.68 (2H, d, *J* = 9.0 Hz, H3'), 7.49 (1H, br s, $CONH_2$), 7.48 (1H, d, *J* = 2.2 Hz, H6), 7.39 (2H, d, *J* = 9.0 Hz, H2'), 7.11 (1H, dd, *J* = 8.8, 2.2 Hz, H4), 6.67 (1H, br s, $CONH_2$), 6.26 (1H, d, *J* = 8.8 Hz, H3); ^{13}C (101 MHz, DMSO) δ 170.9 (C1''), 151.1, 146.6, 146.6, 132.1 (C4), 130.6 (C5), 127.9 (C2'), 125.9 (C3'), 124.0, 123.2 (C6), 117.1, 113.8 (C3); *m/z* (ESI) 256 ([M-H]$^-$); HRMS m/z cald for $C_{13}H_{12}N_3O_3$ 258.0879, found: 258.0888.

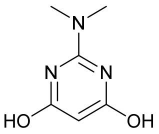

L2

2-(dimethylamino)pyrimidine-4,6-diol (L2).[11] To a stirred solution of sodium ethoxide (1.46 g, 21.4 mmol, 1.1 eq) in ethanol (11 mL) was added 1,1-dimethyl guanidine sulphate (5.1 g, 18.75 mmol, 1.0 eq). Diethyl malonate (3 mL, 18.75 mmol, 1.0 eq) was added to a second stirred solution of sodium ethoxide (2.42 g, 35.6 mmol, 1.9eq) in ethanol (19 mL) before addition of the solution of the dimethyl guanidine solution. The resultant solution was heated at reflux for 5 hrs. After removal of solvent *in vacuo*, water (10 mL) was added to form a colourless solution. Upon adjusting pH to pH 6 using acetic acid, a white emulsion formed. Collection of the white solid by vacuum filtration afforded the title compound as a white solid (1.89 g, 65 %); mp 292 ^{o}C dec.; v_{max}/cm^{-1} 3325 (br, OH str.), 2953 (ArCHstr); ^{1}H NMR (400 MHz, DMSO) δ 10.48 (2H, br. s, OH), 4.66 (1H, s, ArH), 3.00 (6H, s, CH_3); ^{13}C NMR (101 MHz, DMSO) δ 78.00 (ArCH), 75.81 (C-O), 39.31 (C-N), 36.77 (CH_3); *m/z* (ESI) 156 ([M+H]$^{+}$, 100 %), 178 ([M+Na]$^{+}$, 70); HRMS *m/z* cald for $C_6H_{10}N_3O_2$ 156.0773, found: 156.0776.

Catalyst complexes were then prepared as per previous reports.[11, 12]

Suzuki-Miyaura Optimisation Methods

Analytical Scale Suzuki-Miyaura Reactions (Phosphine Complexes). To a microwave vial was added 2-amino-5-bromo benzamide (**2**) (2.0 mM), palladium salt (2.5 mol %), phosphine (6.25 mol %), phenyl boronic acid (1.2 eq) and base (2.0 eq). The vial was then purged with nitrogen before being sealed. Degassed 10 mM potassium phosphate buffer (10 mL, pH 7.2) was then added before heating at 50 ^{o}C overnight. Upon cooling, the reaction was filtered and the resultant solution analysed by LC-MS and NMR.

Analytical Scale Integrated Halogenase-Suzuki-Miyaura Reactions (Pyrimidine and Guanidine Complexes). Halogenase biotransformations were conducted as per the relevant general method above, before being filtered through a 10 kDa MWCO filter (Vivaspin 10). 200 µL aliquots were then transferred to glass vials prior to addition of base and boronic acid. Vials were then crimped closed using lids with rubber septa, prior to degassing with a stream of nitrogen for 5 min. Ligand and palladium were then added as stock solutions in degassed water prior to heating at 80 ^{o}C for 24 hrs. After cooling, reactions were partitioned into Et_2O, CH_2Cl_2 or EtOAc (3 x 200 µL) and dried at 40 ^{o}C. Crude residues were then re-dissolved in MeOH (200 µL) and analysed by analytical HPLC and LC-MS as per the general methods.

General Integrated Halogenase-Suzuki Arylation Methods

Regioselective Arylation using Pure Protein.

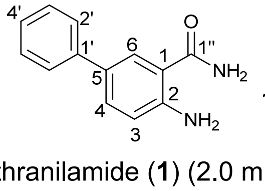

10

2-amino-5-phenyl benzamide (10).[10] Anthranilamide (**1**) (2.0 mM), NaBr (50 mM), D-glucose (40 mM), FAD (1 µM), Fre (2 µM), GDH2 (12 µM), PyrH (20 µM) and NADH (100 µM) were added to potassium phosphate buffer (10 mM, pH 7.2) to a total volume of 30 mL and incubated with shaking at room temperature overnight before filtration through a 10 kDa MWCO filter (Vivaspin 20). K_3PO_4 (15.2 mg, 0.07 mmol, 1.2 eq), and

boronic acid (0.9 mmol, 30 eq) were then added to the filtrate. Following stirring at 50 ^{o}C to dissolve the boronic acid the solution was freeze-thaw degassed and back-filled with nitrogen prior to addition of tppts (2 mM) and Na_2PdCl_4 (1 mM) as deoxygenated stock solutions in water. The resultant solution was then heated at 80 ^{o}C with stirring for 24 hrs. After cooling, reaction was basified with 4N NaOH and extracted into EtOAc (3x 30 mL) before removal of solvent *in vacuo*. Purification by semi-preparative HPLC method 1 afforded the title compound as a colourless solid (10.0 mg, 79 %). v_{max}/cm^{-1} 3433 (N-H str.), 3379 (N-H str.), 3303 (N-H str.), 3177 (N-H str.), 3033 (C-H str.), 2957 (C-H str.), 1640 (N-H str.), 1620 (C=C str.), 1567 (C=O str.); ^{1}H NMR (800 MHz, MeOH) δ 7.81 (1H, d, *J* = 2.2 Hz, H6), 7.59 (2H, d, *J* = 7.8 Hz, H2'), 7.50 (1H, dd, *J* = 8.5, 2.2 Hz, H4), 7.38 (2H, t, *J* = 7.6 Hz, H3'), 7.24 (1H, t, *J* = 7.6 Hz, H4'), 6.83 (1H, d, *J* = 8.5 Hz, H3); ^{13}C NMR (101 MHz, MeOD) δ 174.6 (C1''), 150.5 (C2), 141.9 (C1), 132.2 (C4), 130.3 (C5), 129.7 (C3'), 128.0 (C2), 127.3 (C4'), 127.1 (C6), 118.8 (C3), 116.0 (C1'); *m/z* (ESI) 235 ($[M+Na]^+$, 100 %), 213 ($[M+H]^+$, 45), 196 ($[M-NH_3]^+$, 50); HRMS *m/z* cald for $C_{13}H_{12}N_2ONa$ 235.0857, found: 235.0847; λ_{max} (H_2O/CH_3CN) 278 and 336 nm.

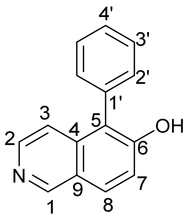

15

5-phenyl-6-hydroxy isoquinoline (15). Synthesised according to general method A as detailed in the experimental section of the main text using phenyl boronic acid. Purification using semi-preparative HPLC method 2 afforded the title compound as an off-white solid (2.9 mg, 66 %); ^{1}H NMR (500 MHz, DMSO) δ 9.51 (1H, s, H1), 8.32 (2H, m, 2 x ArH), 7.64 (1H, d, *J* = 9.0 Hz, ArH), 7.55 (2H, t, *J* = 10.0 Hz, H3'), 7.48 (2H, m, 2 x ArH), 7.35 (2H, d, *J* = 10 Hz, H2'); ^{13}C NMR (101 MHz, DMSO) δ 161.2, 146.1, 139.1, 134.0, 133.3, 132.4, 132.3, 130.7, 128.7, 127.9, 127.3, 122.7, 121.6, 121.2, 120.1; *m/z* (ESI) 222.0 ($[M+H]^+$); HRMS *m/z* cald for $C_{15}H_{12}NO$ 222.0913, found: 222.0894; λ_{max} (H_2O/CH_3CN) 226, 254, 322 nm.

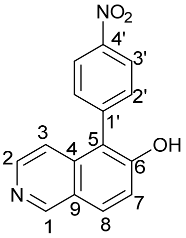

16

4'-nitro-5-phenyl-6-hydroxy isoquinoline (16). Synthesised according to general method A as detailed in the experimental section of the main text using 4-nitrophenyl boronic acid. Purification by semi-preparative HPLC method 2 afforded the title compound as a yellow solid (3.4 mg, 64 %); ^{1}H NMR (800 MHz, CD_3CN) δ 9.34 (1H, s, H1), 8.40 (2H, d, *J* = 9.1, H3'), 8.34 (1H, d, *J* = 9.1, H2), 8.22 (1H, d, *J* = 6.8 Hz, H7), 7.75 (1H, d, *J* = 9.1 Hz, H3), 7.64 (2H, d, *J* = 9.1 Hz, H2'), 7.61 (1H, d, *J* = 6.8 Hz, H8); ^{13}C NMR (201 MHz, CD_3CN) δ 162.2 (C6), 148.8 (ArC), 146.9 (ArC), 146.8 (ArC), 141.6 (ArC), 140.3 (ArC), 133.8 (ArC), 133.6 (ArC), 133.2 (C3'), 124.8 (C2'), 123.9 (ArC), 123.3 (C5), 121.1 (C3), 120.6 (C8); *m/z* (ESI) 265.1 ($[M-H]^-$); HRMS *m/z* cald for $C_{15}H_{11}N_2O_3$ 267.0770, found: 267.0764; λ_{max} (H_2O/CH_3CN) 228, 252, 304, 330 nm.

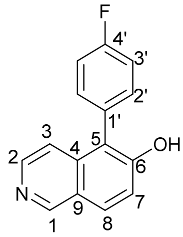

17

4'-fluoro-5-phenyl-6-hydroxy isoquinoline (17). Synthesised according to general method A as detailed in the experimental section of the main text using 4-fluorophenyl boronic acid. Purification by semi-preparative

HPLC method 2 afforded the title compound as a white solid (3.7 mg, 78 %). ^{1}H NMR (400 MHz, CD_3CN) δ 9.33 (1H, s, H1), 8.31 (1H, d, *J* = 9.1 Hz, H7), 8.19 (1H, d, *J* = 6.9 Hz, H2), 7.74 (1H, d, *J* = 9.1 Hz, H8), 7.65 (1H, d, *J* = 6.9 Hz, H3), 7.41 – 7.27 (4H, m, H2' & H3'); ^{13}C NMR (101 MHz, CD_3CN) δ 163.7 (d, $^1J_{CF}$ = 245.4 Hz, C4'), 162.6 (C6), 146.1 (C1), 141.2 (ArC), 133.9 (d, $^3J_{CF}$ = 8.4 Hz, C2'), 133.4 (ArCH), 132.2 (ArCH), 130.0 (d, $^4J_{CF}$ = 3.2 Hz, C1'), 124.3 (ArCH), 123.2 (ArC), 121.8 (ArCH), 116.8 (d, $^3J_{CF}$ = 21.8 Hz, C3'); ^{19}F NMR (470 MHz, CD_3CN) -115.5; *m/z* (ESI) 240.4 ([M+H]$^+$); HRMS *m/z* calc for $C_{15}H_{11}FNO$ 240.0819, found: 240.0814; λ_{max} (H_2O/CH_3CN) 225, 258, 334 nm.

Arylation of benzamides and indoles using CLEAs.

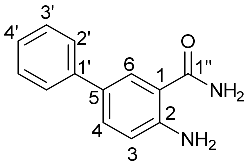

10

2-amino-5-phenyl benzamide (10).[10] Synthesised from anthranilamide (**1**) using PyrH CLEAs, K_3PO_4 and phenyl boronic acid according to general method B as detailed in the experimental section of the main text. After cooling, reaction was basified using 4N NaOH and partitioned into EtOAc. Combined organics were then concentrated *in vacuo* and purified by semi-preparative HPLC method 1 to afford the title compound as a white solid (16.0 mg, 84 %). The reaction was also conducted on a 1.0 mmol scale using anthranilamide (**1**) (5.0 mM), PyrH CLEAs from 3 L in a total reaction volume of 200 mL. The biotransformation was conducted over 8 days, with addition of isopropanol (5% v/v) every 48 hrs. After removal of CLEA using centrifugation (10000 rpm, 30 min, 4 ^{o}C), phenyl boronic acid and K_3PO_4 were added to the supernatant prior to deoxygenation under a stream of nitrogen. Tppts (0.2 mM) and Na_2PdCl_4 (0.1 mM) were then added prior to heating at 80 ^{o}C for 24 hrs. After cooling and workup as described in general method B of the experimental section of the main text, purification by flash chromatography (SiO_2; Et_2O) afforded the title compound as a white solid (110 mg, 52 %). Spectra were consistent with those reported herein.

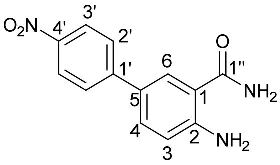

11

2-amino-4'-nitro-5-phenyl benzamide (11). Synthesised from anthranilamide (**1**) using PyrH CLEAs, K_3PO_4 and 4-nitrophenyl boronic acid according to general method B as detailed in the experimental section of the main text. After cooling, reaction was basified using 4N NaOH and partitioned into EtOAc. Combined organics were then concentrated *in vacuo* and purified by semi-preparative HPLC method 1 to afford the title compound as a yellow solid (18.8 mg, 81 %); ^{1}H NMR (400 MHz, CD_3CN) δ 8.25 (2H, m, H3'), 7.89 – 7.79 (3H, m, H2'+ H6), 7.62 (1H, dd, *J* = 8.6, 2.3 Hz, H4), 6.84 (1H, d, *J* = 8.6 Hz, H3); ^{13}C NMR (126 MHz, CD_3CN) δ 172.08 (C1''), 151.84 (ArC), 147.83 (ArC), 147.13 (Arc), 132.23 (ArCH), 128.75 (ArCH), 127.25 (ArCH), 126.04 (ArC), 125.05 (ArC), 118.32 (ArCH), 114.99 (ArC); *m/z* (ESI) 256 ([M-H]$^-$, 100 %); HRMS *m/z* cald for $C_{13}H_{11}O_3N_3$ 257.0752, found: 257.0771; λ_{max} (EtOH) 325 nm.

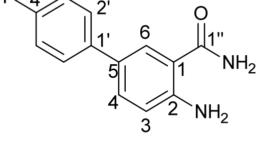

12

2-amino-4'-fluoro-5-phenyl benzamide (12). Synthesised from anthranilamide (**1**) using PyrH CLEAs, K_3PO_4 and 4-fluorophenyl boronic acid according to general method B as detailed in the experimental section of the main text. After cooling, reaction was basified using 4N NaOH and partitioned into EtOAc. Combined organics were then concentrated *in vacuo* and purified by semi-preparative HPLC method 1 to afford the title compound as an off-white solid (15.1 mg, 73 %); ^{1}H NMR (400 MHz, CD_3CN) δ 7.72 (1H, d, *J* = 2.2 Hz, H6), 7.62 (2H, m, H3'), 7.52 (1H, dd, *J* = 8.4, 2.2 Hz, H4), 7.17 (2H, m, H2'), 6.86 (1H, d, *J* = 8.5

Hz, H3); ^{13}C NMR (101 MHz, MeOD) δ 173.6 (C1''), 163.5 (d, $^{1}J_{CF}$ = 244.3 Hz, C4'), 146.3, 137.8, 132.1, 132.1, 129.1 (d, $^{3}J_{CF}$ = 8.0 Hz, C2'), 128.1, 120.5, 118.5, 116.4 (d, $^{3}J_{CF}$ = 21.6 Hz, C3'); ^{19}F NMR (376 MHz, CD_3CN) δ -118.38; *m/z* (ESI) 321.0 ($[M+H]^+$, 40 %), 253.0 ($[M+Na]^+$, 100); HRMS *m/z* cald for $C_{13}H_{11}N_2OFNa$ ($[M+Na]^+$) 253.0753, found: 253.0763.

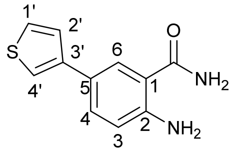

13

2-amino-5-(thiophen-3-yl) benzamide (13). Synthesised from anthranilamide (**1**) using PyrH CLEAs, K_3PO_4 and thiophen-3-ylboronic acid according to general method B as detailed in the experimental section of the main text. After cooling the reaction was partitioned into EtOAc and combined organics concentrated under a stream of nitrogen and purified by semi-preparative HPLC method 1 to afford the title compound as a yellow solid (13 mg, 71 %); ^{1}H NMR (400 MHz, CD_3CN) δ 7.27 (1H, d, *J* = 2.0 Hz, H6), 7.06 (1H, dd, *J* = 8.4, 2.0 Hz, H4), 6.97 (3H, m, H1', H2' & H4'), 6.30 (1H, d, *J* = 8.4 Hz, H3). ^{13}C NMR (101 MHz, CD_3CN) δ 160.6 (C=O), 159.0 (ArC), 131.6 (C4), 127.4 (ArCH), 127.3 (ArC), 126.9 (ArCH), 126.9 (ArCH), 124.8 (ArC), 119.1 (ArCH), 117.3 (C6); *m/z* (ESI) 202.0 ($[M-NH_2]^+$, 100 %), 219.0 ($[M+H]^+$, 5), 241.0 ($[M+Na]^+$, 20); HRMS *m/z* cald for $C_{11}H_8NOS$ ($[M-NH_2]^+$) 202.0321, found: 202.0323; λ_{max} (H_2O/MeCN) 220, 276, 348 nm.

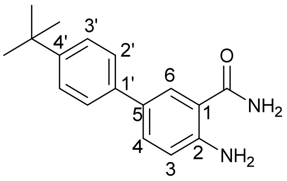

14

2-amino-4'-(tert-butyl)-5-phenyl benzamide (14). Synthesised from anthranilamide (**1**) using PyrH CLEAs, K_3PO_4 and 4-(tertbutyl) phenyl boronic acid according to general method B as detailed in the experimental section of the main text. After cooling, reaction was basified using 4N NaOH and partitioned into EtOAc. Combined organics were then concentrated *in vacuo* and purified by semi-preparative HPLC method 1 to afford the title compound as a white solid (14 mg, 65 %); ^{1}H NMR (400 MHz, DMSO) δ 7.97 (1H, br s, $CON\underline{H_2}$), 7.84 (1H, d, *J* = 2.0 Hz, H6), 7.56 (2H, d, *J* = 8.0 Hz, H2'), 7.49 (1H, dd, *J* = 8.4, 2.0 Hz, H4), 7.41 (2H, d, *J* = 8.0 Hz, H3'), 7.18 (1H, br s, $CON\underline{H_2}$), 6.81 (1H, d, *J* = 8.4 Hz, H3), 1.30 (9H, s, $C\underline{Me_3}$); ^{13}C NMR (201 MHz, DMSO) δ 171.1 (C=O), 158.2 (ArC), 158.1 (ArC), 148.5 (ArC), 137.2 (ArC), 130.0 (C4), 126.7 (ArCH), 125.5 (ArCH), 125.4 (ArCH), 117.5 (C3), 34.1 ($\underline{C}Me_3$), 31.2 ($C\underline{Me_3}$); *m/z* (ESI) 252.1 ($[M-NH_2]^+$, 100 %) 269.1 ($[M+H]^+$, 30); HRMS *m/z* cald for $C_{17}H_{18}NO$ ($[M-NH_2]^+$) 252.1383, found: 252.1385; λ_{max} (H_2O/MeCN) 210, 283, 347. nm.

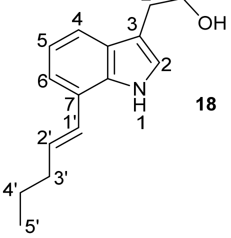

18

7-(pent-1-en-1-yl) tryptophol (18). Synthesised from tryptophol (**3**) using RebH CLEAs, K_3PO_4 and E-pent-1-en-1-yl boronic acid according to general method B as detailed in the experimental section of the main text. After cooling, reaction was partitioned into CH_2Cl_2 (3 x 30 mL) and combined organics concentrated *in* vacuo. Purification by semi-preparative HPLC method 3 afforded the title compound as a white solid (15 mg, 75 %); ^{1}H NMR (400 MHz, MeOD) δ 7.41 (1H, dd, *J* = 7.4, 0.8 Hz, H6), 7.17 (1H, d, *J* = 7.4 Hz, H4), 7.08 (1H, s, H2), 6.97 (1H, t, *J* = 7.6 Hz, H5), 6.76 (1H, d, *J* = 16 Hz, H1'), 6.32 (1H, dt, *J* = 16, 7.2 Hz, H2'), 3.80 (2H, t, *J* = 7.2 Hz, H1''), 2.96 (2H, t, *J* = 7.2 Hz, H2''), 2.28 (2H, qd, *J* = 7.2, 1.6 Hz, H3'), 1.54 (2H, sextet, *J* = 7.6 Hz, H4'), 1.01 (3H, t, *J* = 7.6 Hz, H5'); ^{13}C NMR (101 MHz, MeOD) δ 135.6 (ArC), 131.9 (ArC), 129.5 (ArC), 127.4 (ArC), 123.7 (ArC), 123.1 (ArC), 119.9 (ArC), 119.2 (ArC), 118.1 (ArC), 113.0 (ArC), 63.7, 61.2,

38.8, 36.7, 29.8, 23.8, 14.1; *m/z* (ESI) 230.1 ($[M+H]^+$); HRMS *m/z* cald for $C_{15}H_{20}NO$ 230.1539, found: 230.1467; λ_{max} (H_2O/CH_3CN) 238, 310 nm.

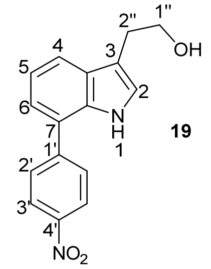

19

4'-nitro-7-phenyl tryptophol (19). Synthesised from tryptophol (**3**) using RebH CLEAs, K_3PO_4 and 4-nitrophenyl boronic acid according to general method B as detailed in the experimental section of the main text. After cooling, reaction was partitioned into CH_2Cl_2 (3 x 30 mL) before concentration *in vacuo*. Purification by semi-preparative HPLC method 3 afforded the title compound as a yellow solid (19.1 mg, 75 %); ^{1}H NMR (400 MHz, MeOD) δ 8.41 – 8.36 (2H, m, H3'), 7.90 – 7.87 (2H, m, H2'), 7.65 (1H, dd, *J* = 7.8, 1.1 Hz, ArH), 7.24 – 7.14 (3H, m, 3ArH), 3.84 (2H, t, *J* = 7.0 Hz, H1''), 3.02 (2H, t, *J* = 7.0 Hz, H2''); ^{13}C NMR (101 MHz, MeOD) δ 148.1, 147.8, 135.0, 130.2, 125.0, 124.7, 124.5, 122.9, 120.3, 120.3, 113.6, 63.5 (C1''), 29.69 (C2''); *m/z* (ESI) 281.1 ($[M-H]^-$); HRMS *m/z* cald for $C_{16}H_{13}N_2O_2$ ($[M-H]^-$) 281.0932, found: 281.0915; λ_{max} (H_2O/CH_3CN) 261, 352 nm.

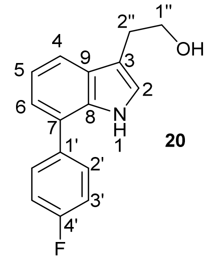

20

4'-fluoro-7-phenyl tryptophol (20). Synthesised from tryptophol (**3**) using RebH CLEAs, K_3PO_4 and 4-fluorophenyl boronic acid according to general method B as detailed in the experimental section of the main text. After cooling, reaction was partitioned into CH_2Cl_2 (3 x 30 mL) and combined organics concentrated *in* vacuo. Purification by semi-preparative HPLC method 3 afforded the title compound as a white solid (16.3 mg, 71 %); ^{1}H NMR (800 MHz, CD_3CN) δ 9.06 (1H, s, NH), 7.81 (2H, m, H3'), 7.57 (1H, dd, *J* = 8.0, 1.0 Hz, H6), 7.38 (1H, d, *J* = 8.0, H4), 7.11 – 7.07 (3H, M, H2+2'), 7.03 (1H, ddd, *J* = 8.0, 7.0, 1.0 Hz, H5), 3.74 (2H, t, *J* = 7.0 Hz, H1''), 2.92 (2H, t, *J* = 7.0, H2''); ^{13}C NMR (201 MHz, CD_3CN) δ 165.4 (d, $^1J_{CF}$ = 246.6 Hz, 4'C), 137.3 (d, $^4J_{CF}$ = 8.1 Hz, 1'C), 128.6 (ArC), 125.9 (C4), 123.7 (C2), 122.3 (C6), 119.5 (d, $^2J_{CF}$ = 25.2 Hz, C3'), 118.4 (ArC), 115.3 (d, $^3J_{CF}$ = 20.1 Hz, C2'), 114.1 (ArC), 113.1 (ArC), 112.1 (ArC), 63.1 (C1''), 29.5 (C2''); *m/z* (ESI) 254.3 ($[M-H]^-$); HRMS *m/z* cald for $C_{16}H_{13}FNO$ ($[M-H]^-$) 254.0987, found: 254.0992; λ_{max} (H_2O/CH_3CN) 242 nm.

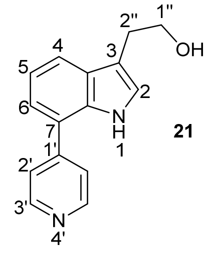

21

7-(pyrid-4-yl) tryptophol (21). Synthesised from tryptophol (**3**) using RebH CLEAs, K_3PO_4 and pyrid-4-yl boronic acid according to general method B as detailed in the experimental section of the main text. After cooling, reaction was partitioned into CH_2Cl_2 (3 x 30 mL) and combined organics concentrated *in* vacuo.

Purification by semi-preparative HPLC method 3 afforded the title compound as an off-white solid (14.6 mg, 68 %); ^{1}H NMR (400 MHz, MeOD) δ 8.62 (2H, d, *J* = 8.0 Hz, H3'), 7.72 (2H, m, H2'), 7.66 (1H, dd, *J* = 8.0, 1.0 Hz, H6), 7.25 (dd, *J* = 8.0, 1.0 Hz, H4), 7.18 – 7.15 (2H, m, H5 & H2), 3.83 (2H, dd, *J* = 7.2 Hz, H1''), 3.01 (2H, dd, *J* = 7.2 Hz, H2''); ^{13}C NMR (101 MHz, MeOD) δ 150.5 (ArC), 150.0 (ArC), 134.9 (ArC), 130.3 (ArC), 124.8 (ArC), 122.7 (ArC), 120.8 (ArC), 120.3 (ArC), 113.7 (ArC), 63.6 (C1''), 29.7 (C2''); *m/z* (ESI) 239.1 ([M+H]$^+$); HRMS *m/z* cald for $C_{15}H_{15}N_2O$ 239.1179, found: 239.1167; λ_{max} (H_2O/CH_3CN) 226, 250, 376 nm.

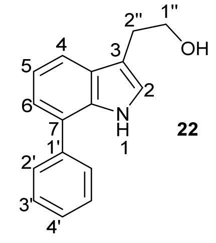

22

7-phenyl tryptophol (22). Synthesised from tryptophol (**3**) using RebH CLEAs, K_3PO_4 and phenyl boronic acid according to general method B as detailed in the experimental section of the the main text. After cooling, reaction was partitioned into CH_2Cl_2 (3 x 30 mL) before concentration *in vacuo*. Purification by semi-preparative HPLC method 3 afforded the title compound as a white solid (11.5 mg, 54 %); ^{1}H NMR (400 MHz, CD_3CN) δ 9.15 (1H, s, H1), 7.65 (2H, m, H2'), 7.59 (1H, d, *J* = 8 Hz, H6), 7.52 (2H, m, H3'), 7.42 (1H, m, H4'), 7.19 – 7.13 (2H, m, 2 x ArH), 7.12 (1H, m, H2), 3.78 (2H, t, *J* = 7.0 Hz, H1''), 2.96 (2H, m, H2''); ^{13}C NMR (101 MHz, MeOD) δ 141.0, 135.4, 129.9, 129.8, 129.4 128.1, 127.0, 124.2, 122.4, 120.2, 118.7, 113.2, 63.7 (C1''), 29.8 (C2''); *m/z* (ESI) 238.1 ([M+H]$^+$); HRMS *m/z* cald for $C_{16}H_{16}NO$ ([M+H]$^+$) 238.1226, found: 238.1224; λ_{max} (H_2O/CH_3CN) 261, 352 nm.

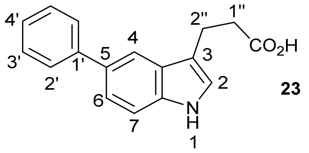

23

5-phenyl indole-3-propionic acid (23). Synthesised from 3-indole propionic acid (**5**) using PyrH CLEAs, K_2CO_3 and phenyl boronic acid and K_2CO_3 according to general method B as detailed in the experimental section of the main text. After cooling, the reaction was adjusted to pH 1 using 1M HCl and extracted into EtOAc (3 x 30 mL). Solvent was then removed *in vacuo* prior to purification by semi-preparative HPLC method 3. The title compound was isolated as a white solid (16.2 mg, 68 %); ^{1}H NMR (400 MHz, MeOD) δ 7.76 (1H, m, H4), 7.65 (2H, m, H2'), 7.42 – 7.38 (4H, 4 x ArH), 7.26 (1H, m, H4'), 7.09 (1H, s, H2), 3.11 (2H, t, *J* = 7.5 Hz, H1''), 2.70 (2H, td, *J* = 7.6, 2.6 Hz, H2''); ^{13}C NMR (101 MHz, MeOD) δ 178.1 (CO), 130.3, 130.2, 128.8, 128.7, 128.0, 127.6, 124.3, 122.6, 120.1, 118.1, 113.1, 111.1, 36.7 (C1''), 22.4 (C2''); *m/z* (ESI) 266.2 ([M+H]$^+$, 100); HRMS *m/z* cald for $C_{17}H_{14}NO_2$ ([M-H]$^-$) 264.1030, found: 264.1032; λ_{max} (H_2O/CH_3CN) 286, 324 nm.

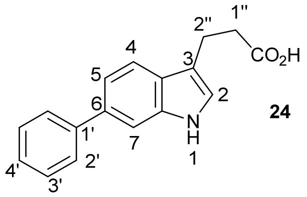

24

6-phenyl indole-3-propionic acid (24). A 30 mL bromination of 3-indole propionic acid (**5**) using SttH CLEAs was assembled as per general method B in the experimental section of the main text. After incubation at room temperature overnight, the biocatalyst was removed using centrifugation (10000 rpm, 4

^{o}C, 30 min) before re-suspension into fresh reaction buffer containing 3-indole propionic acid (**5**) and incubation at room temperature overnight. This process was repeated one more time before addition of phenyl boronic acid and K_2CO_3 to the combined supernatants and freeze-thaw degassing and nitrogen backfill. Tppts (20 mol %) and Na_2PdCl_4 (10 mol %) were then added as deoxygenated solutions in water and the reaction heated to 80 ^{o}C overnight. After cooling, the reaction was adjusted to pH 1 and extracted into EtOAc (3 x 30 mL). Solvent was then removed *in vacuo* and the crude purified by semi-preparative HPLC method 3 to afford the title compound was an off-white solid (53.7 mg, 75 %); ^{1}H NMR (400 MHz, CD_3CD_2OD) δ 7.70-7.69 (3H, m, H2'+2), 7.64 (1H, d, *J* = 8.3 Hz, H4), 7.45 (2H, t, *J* = 8 Hz, H3'), 7.34 (1H, t, *J* = 8 Hz, H4'), 7.30 (1H, m, H5), 6.49 (1H, m, H7), 4.36 (2H, d, *J* = 6.9 Hz, H1''), 2.76 (2H, t, *J* = 6.9 Hz, 2''); ^{13}C NMR (101 MHz, MeOD) δ 177.4 (CO), 129.6, 129.6, 128.1, 128.1, 127.4, 127.0, 123.7, 122.0, 119.5, 117.5, 112.5, 110.5, 36.1 (C1''), 21.7 (C2''); *m/z* (ESI) 264.1 ([M-H]$^{-}$); HRMS *m/z* cald for $C_{17}H_{14}NO_2$ ([M-H]$^{-}$) 264.1030, found: 264.1009; λ_{max} (H_2O/CH_3CN) 256, 312 nm.

General Method for Integrated Arylation with Pure Protein Recycling. A PyrH biotransformation of anthranilamide (**1**) (2.0 mM) was as set up to total volume of 15 mL as per the general method detailed above and incubated at room temperature. After 3 hrs, reaction was concentrated to 500 µL using a 10 kD MWCO filter (Vivaspin 10) and 100 µL of filtrate taken for analysis by HPLC. The concentrate was then diluted with potassium phosphate buffer (10 mM, pH 7.2) before addition of further anthranilamide (2.0 mM), NADH (100 µM), FAD (1 µM), NaBr (100 mM) and glucose (20 mM) to total volume of 15 mL. The process was repeated after 3 hrs and the reaction left at room temperature overnight. The combined filtrates were then subjected to the Suzuki coupling conditions detailed above and purified by semi-preparative HPLC method 1 to afford 5-phenyl anthranilamide at a white solid (10.3 mg, 54 %). Spectra were consistent with those previously reported herein.

PDMS Compartmentalisation

PDMS Thimble Preparation. Thimbles were prepared using a modification on the processes used by Bowden and Gröger.[13, 14] Elastomer and curing agent of a Sylgard 184 kit (Sigma Aldrich) were combined and mixed thoroughly with a spatula before degassing under static vacuum for 1 hr. Plasma cleaned vials (15 mins.) were then placed under static vacuum with 1 mL of trichloro(1H,1H,2H,2H-perfluorooctyl)silane as silanizer for 2 hrs. Vials were dipped into the Sylgard mixture prior to partial curing upside down on a plastic petri dish at 70 ^{o}C for 1 hr. Vials were then dipped for a second time and baked overnight at 70 ^{o}C for cross-linking. After using a razor blade to cut around the top of the vial and free them from the petri dish, vials were soaked in hexanes for 30 min. for swelling. After separating the vial from the PDMS layer, thimbles were removed from hexane and soaked in dichloromethane twice for 2 hrs. each time. Vials were then dried at 70 ^{o}C overnight before use.

PDMS Flux Determination. To the thimbles prepared above was added 1 mL of potassium phosphate buffer (10 mM, pH = 7.2). The thimble was then placed in a beaker containing either 5-bromo anthranilamide (2.5 mM), glucose (20 mM), NADH (2 mM) or FAD (22 µM) in potassium phosphate buffer (10 mM, pH = 7.2) to total volume of 10 mL. 100 µL aliquots from both inside and outside the thimble were then taken at the timepoints stated (Supplementary Figure 12). In the case of **L2**$_2$.Pd(OAc)$_2$, catalyst complex was added to the inside of the thimble (10 mM) and timepoints taken as stated (Supplementary Figure 8), plus one after being left overnight. Timepoint samples were then transferred into a UV-clear 96 well plate and UV absorbance measured. Absorbance was then compared against a calibration curve of each reagent and the concentration inside and outside the thimble at each timepoint determined (see Supplementary Figure 8).

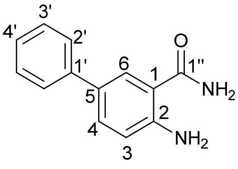

10

5-phenyl anthranilamide (10).[10] Synthesised from anthranilamide (**1**) using purified PyrH, phenyl boronic acid (10 mM), CsF (20 mM) and **L2**.Pd(OAc)$_2$ (0.2 mM) according to general method C as detailed in the experimental section of the main text to afford the title compound as an off-white solid (9.4 mg, 74 %). Spectra are consistent with those presented herein.

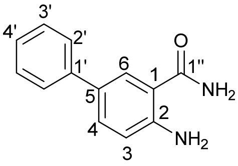

10

5-phenyl anthranilamide (10).[10] Synthesised from anthranilamide (**1**) using PyrH CLEAs, phenyl boronic acid (25 mM), CsF (50 mM) and **L2**$_2$.Pd(OAc)$_2$ (0.1 mM) according to general method D as detailed in the experimental section of the main text to afford the title compound as an off-white solid (11.3 mg, 59 %). Spectra are consistent with those presented herein.

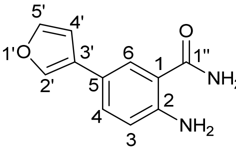

25

2-amino-5-(furan-3-yl) benzamide (25). Prepared from anthranilamide (**1**) using PyrH CLEAs, furan-3-boronic acid (25 mM), CsF (50 mM) and **L2**$_2$.Pd(OAc)$_2$ (0.5 mM) according to general method D as detailed in the experimental section of the main text to afford the title compound as an orange solid (19.2 mg, 64 %); ^{1}H NMR (400 MHz, MeOD) δ 8.00 (1H, d, *J* = 2.0 Hz, H6), 7.95 (1H, s, H2'), 7.68 (1H, dd, *J* = 8.4, 2.0 Hz, H4), 7.58 (1H, m, H4'), 7.17 (1H, d, *J* = 8.4 Hz, H3), 6.87 (1H, m, H5'); ^{13}C NMR (101 MHz, MeOD) δ 174.4 (C1''), 149.9 (ArC), 144.6 (C4'), 138.6 (C2'), 131.2 (C4), 127.4 (ArC), 126.7 (ArC), 122.0 (C3), 118.8 (ArC), 115.8 (ArC), 109.4 (C5');*m/z* (ESI) 186.1 ([M-NH$_3$]$^+$, 80 %), ([M+H]$^+$, 60 %), ([M+Na]$^+$, 100); HRMS *m/z* calcd for $C_{11}H_{10}O_2N_2$ 202.0742, found: 202.0740; λ_{max} (H_2O/CH_3CN) 229, 292 nm.

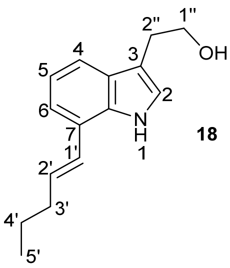

18

7-(pent-1-en-1-yl) tryptophol (18). Prepared from tryptophol (**3**) using RebH CLEAs, and E-pent-1-en-1-yl boronic acid (25 mM), CsF (50 mM) and **L2**$_2$.Pd(OAc)$_2$ (0.5 mM) according to general method D as detailed in the experimental section of the main text to afford the title compound as a white solid (19 mg, 61 %). Spectra are consistent with those reported herein.

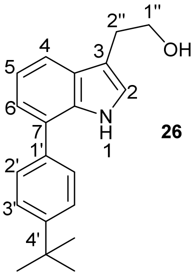

26

4’-tertbutyl-7-phenyl tryptophol (26). Prepared from tryptophol (**3**) using RebH CLEAs, and 4-tertbutyl phenyl boronic acid (25 mM), CsF (50 mM) and **L2**$_2$.Pd(OAc)$_2$ (0.5 mM) according to general method D as detailed in the experimental section of the main text to afford the title compound as a white solid (26.8 mg, 61 %). ^{1}H NMR (400 MHz, MeOD) δ 7.59 – 7.49 (5H, m, ArH), 7.15 – 7.04 (3H, m, ArH), 3.83 (2H, t, *J* = 7.4 Hz, H1”), 3.01 (d, *J* = 7.4 Hz, H2”), 1.38 (9H, s, C$\underline{Me}_3$); ^{13}C NMR (101 MHz, MeOD) δ 151.09 (ArC), 138.02 (ArC), 135.45 (ArC), 129.7 (ArC), 129.0 (Ar$\underline{C}$H), 126.9 (ArC), 126.8 (Ar$\underline{C}$H), 124.1 (Ar$\underline{C}$H), 122.3 (Ar$\underline{C}$H), 120.2 (Ar$\underline{C}$H), 118.5 (Ar$\underline{C}$H), 113.1 (ArC), 63.7 (C1”), 35.4 ($\underline{C}$Me$_3$), 31.8 (C$\underline{Me}_3$), 29.9 (C2”); *m/z* (ESI) 294.2 ([M+H]$^+$); HRMS calcd for $C_{20}H_{24}NO$ 294.1852, found: 294.1911; λ_{max} (H_2O/CH_3CN) 224, 285 nm.

Supplementary References

1. Hosford, J., Shepherd, S.A., Micklefield, J. & Wong, L.S. A High-Throughput Assay for Arylamine Halogenation Based on a Peroxidase-Mediated Quinone–Amine Coupling with Applications in the Screening of Enzymatic Halogenations. *Chem Eur J* **20**, 16759-16763 (2014).
2. Shepherd, S.A. et al. Extending the biocatalytic scope of regiocomplementary flavin-dependent halogenase enzymes. *Chem Sci* **6**, 3454-3460 (2015).
3. Frese, M., Guzowska, P.H., Voß, H. & Sewald, N. Regioselective Enzymatic Halogenation of Substituted Tryptophan Derivatives using the FAD-Dependent Halogenase RebH. *Chem Cat Chem* **6**, 1270-1276 (2014).
4. Frese, M. & Sewald, N. Enzymatic Halogenation of Tryptophan on a Gram Scale. *Angew Chem Int Ed* **54**, 298-301 (2015).
5. Cheng, X., Vellalath, S., Goddard, R. & List, B. Direct Catalytic Asymmetric Synthesis of Cyclic Aminals from Aldehydes. *J Am Chem Soc* **130**, 15786-15787 (2008).
6. Adachi, H. et al. Structure−Activity Relationships of 2,N6,5‘-Substituted Adenosine Derivatives with Potent Activity at the A2B Adenosine Receptor. *J Med Chem* **50**, 1810-1827 (2007).
7. Pereira, R. et al. Indole-Derived Psammaplin A Analogues as Epigenetic Modulators with Multiple Inhibitory Activities. *J Med Chem* **55**, 9467-9491 (2012).
8. Beugelmans, R., Roussi, G., González Zamora, E. & Carbonnelle, A.-C. Synthetic studies towards western and eastern macropolypeptide subunits of kistamycin. *Tetrahedron* **55**, 5089-5112 (1999).
9. Plettenburg, O. et al. Isoquinoline Derrivatives ad Inhibitors of RHO-Kinase. WO2007000240 (2007).
10. Vaughan, J. & Christopher, J. O-Hydroxy and O-Amino Benzamide Derivatives as IKK2 Inhibitors. WO2007025575 (2007).
11. Chalker, J.M., Wood, C.S.C. & Davis, B.G. A Convenient Catalyst for Aqueous and Protein Suzuki−Miyaura Cross-Coupling. *J Am Chem Soc* **131**, 16346-16347 (2009).
12. Gao, Z., Gouverneur, V. & Davis, B.G. Enhanced Aqueous Suzuki-Miyaura Coupling Allows Site-Specific Polypeptide 18F-Labeling. *J Am Chem Soc* **135**, 13612-13615 (2013).
13. Runge, M.B., Mwangi, M.T., Miller, A.L., Perring, M. & Bowden, N.B. Cascade Reactions Using LiAlH4 and Grignard Reagents in the Presence of Water. *Angew Chem Int Ed* **47**, 935-939 (2008).
14. Sato, H., Hummel, W. & Gröger, H. Cooperative Catalysis of Noncompatible Catalysts through Compartmentalization: Wacker Oxidation and Enzymatic Reduction in a One-Pot Process in Aqueous Media. *Angew Chem Int Ed* **54**, 4488-4492 (2015).
